# riceExplorer: Uncovering the Hidden Potential of a National Genomic Resource Against a Global Database

**DOI:** 10.3389/fpls.2022.781153

**Published:** 2022-04-29

**Authors:** Clive T. Darwell, Samart Wanchana, Vinitchan Ruanjaichon, Meechai Siangliw, Burin Thunnom, Wanchana Aesomnuk, Theerayut Toojinda

**Affiliations:** National Center for Genetic Engineering and Biotechnology (BIOTEC), Khlong Luang, Thailand

**Keywords:** crop breeding, deleterious variants, bioinformatics, genomics, elite cultivars, non-focal varieties

## Abstract

Agricultural crop breeding programs, particularly at the national level, typically consist of a core panel of elite breeding cultivars alongside a number of local landrace varieties (or other endemic cultivars) that provide additional sources of phenotypic and genomic variation or contribute as experimental materials (e.g., in GWAS studies). Three issues commonly arise. First, focusing primarily on core development accessions may mean that the potential contributions of landraces or other secondary accessions may be overlooked. Second, elite cultivars may accumulate deleterious alleles away from nontarget loci due to the strong effects of artificial selection. Finally, a tendency to focus solely on SNP-based methods may cause incomplete or erroneous identification of functional variants. In practice, integration of local breeding programs with findings from global database projects may be challenging. First, local GWAS experiments may only indicate useful functional variants according to the diversity of the experimental panel, while other potentially useful loci—identifiable at a global level—may remain undiscovered. Second, large-scale experiments such as GWAS may prove prohibitively costly or logistically challenging for some agencies. Here, we present a fully automated bioinformatics pipeline (riceExplorer) that can easily integrate local breeding program sequence data with international database resources, without relying on any phenotypic experimental procedure. It identifies associated functional haplotypes that may prove more robust in determining the genotypic determinants of desirable crop phenotypes. In brief, riceExplorer evaluates a global crop database (IRRI 3000 Rice Genomes) to identify haplotypes that are associated with extreme phenotypic variation at the global level and recorded in the database. It then examines which potentially useful variants are present in the local crop panel, before distinguishing between those that are already incorporated into the elite breeding accessions and those only found among secondary varieties (e.g., landraces). Results highlight the effectiveness of our pipeline, identifying potentially useful functional haplotypes across the genome that are absent from elite cultivars and found among landraces and other secondary varieties in our breeding program. riceExplorer can automatically conduct a full genome analysis and produces annotated graphical output of chromosomal maps, potential global diversity sources, and summary tables.

## Introduction

Most modern agricultural crop breeding programs feature a core panel of elite plant accessions (hereafter termed *elite cultivars*; ECs) that serve as the focal target for improved phenotypes (Tokatlidis, 2015). Primarily, a breeding program’s goals are to produce higher yield varieties that are also resilient to a range of biotic and abiotic stressors commonly encountered across the geographical extent of the program (typically national boundaries). Alongside endemic ECs, a number of local landrace varieties, endemic cultivars, and other non-commercial varieties (hereafter termed *non-focal varieties*; NFVs) provide additional sources of regionally apposite phenotypic and genomic adaptive variation and contribute as experimental material that provide statistical power during investigative evaluation (e.g., in genome-wide association studies; GWAS) (Tokatlidis and Vlachostergios, 2016). A key issue is that detailed cataloging that facilitates comprehensive understanding of the genomic potential of NFVs may be neglected as breeding programs develop with a focus on elite accessions (Azeez et al., 2018). For example, the value of rare variants may be overlooked, and their functional significance buried among results in large-scale experiments (Zuk et al., 2014). Furthermore, modern breeding methodologies generally exert strong positive selection meaning that ECs typically feature reduced levels of genomic variation and elevated levels of accumulated deleterious mutants compared to both NFVs and wild relatives (Moyers et al., 2018).

These genomic “costs of domestication” may originate from a number of sources. Lu et al. (2006) noted elevated levels of non-synonymous substitutions relative to wild rice lineages suggested to have hitchhiked along with the targets of artificial selection. Furthermore, domestication inevitably increases levels of inbreeding or equivalent processes *via* induced switching from outbreeding mating systems (Kovach et al., 2007), reductions in effective population size (N*_e_*), increases in linkage disequilibrium (LD; see: Hartfield and Otto, 2011), or *via* artificial selection. In general, these processes increase levels of homozygosity and the likelihood of deleterious action at affected loci (e.g., Lindblad-Toh et al., 2005). Inbreeding depression *via* concomitant increases in homozygosity and LD (Hufford et al., 2012), alongside reduced N*_e_*, renders selection less efficient at purging moderately deleterious mutations and novel beneficial mutations are more likely lost to genetic drift.

In general, deleterious effects may disproportionately accumulate across the genomes of ECs. Moreover, the large and ever-growing numbers of identified genes associated with various key traits (Wing et al., 2018) suggests that influential markers lie scattered across the genome, including those that have yet to be functionally identified. Thus, although many affected loci may not mediate direct functional control over key agronomic traits or targets of selection, there may be a general degradation of the genetic background. Moreover, some affected loci may have pleiotropic effects on specific targets of selection or other incidental, functional traits that are indirectly beneficial to domesticated crops (Darmency, 2013; Paaby and Rockman, 2013). In order to monitor the progress of these dynamics and to understand where useful genomic variation may have been (a) eroded, and (b), where recuperative variation may reside among a breeding program’s resources, it would be useful to have an automated cataloging system.

A further consideration is that breeding programs may be somewhat insular by nature. Experimental investigation uses available materials which may overwhelmingly comprise of endemic varieties with a smaller proportion of material contributed by external sources (in the case of national programs, these may be from collaborative relationships with neighboring countries or from international consortia). For example, in a GWAS, genomic variants exhibiting significant correlations with investigated phenotypes will be identified in the context of the accession panel being investigated (Korinsak et al., 2021). Other potentially useful variants will be overlooked. Moreover, trying to overcome this issue by performing ever larger GWAS experiments may prove logistically challenging (in terms of sourcing experimental resources, time, or infrastructure) or financially inviable due to labor and equipment costs.

Furthermore, while GWAS methodologies have become a commonplace tool in identifying key functional variants they suffer from some inherent drawbacks. These include low-informativeness of SNP markers (Collard et al., 2005), the influence of rare variants associated with extreme phenotypes (Wray et al., 2013), and the confounding influence of linkage disequilibrium (Platt et al., 2010; Korte and Farlow, 2013). Additionally, they do not account for epistatic interactions that may account for functional effectiveness (Clark, 2004; Bardel et al., 2005). Thus, haplotype mining facilities offer potentially augmentative technologies in genomics-assisted breeding (Bhat et al., 2021). Haplotype identification has been shown to benefit from both increased informativeness (Hamblin and Jannink, 2011), and also incorporates epistatic relationships within identified genomic regions. Thus, functional haplotype identification has been proposed as an important tool in genomics-assisted breeding that can improve genomic prediction capabilities (Liu et al., 2019; Robertsen et al., 2019; Yu et al., 2019; Bhat et al., 2021).

In Thailand, rice is the primary staple food crop, and the country has a well-developed rice improvement research infrastructure spanning several large institutions. Thailand has fully embraced the omics age and also has ongoing genetic modification/editing programs in operation involving rice and other crops (Napasintuwong, 2019). In recent GWAS studies performed on Thai rice, study materials comprise landrace or other local NFVs alongside around 35 elite lines that are the primary focus of rice improvement strategies at the national and export market levels (Sattayachiti et al., 2020; Korinsak et al., 2021). Significant SNP markers have been identified that tally with those identified in previous studies based on different panels while further SNPs have been identified which may eventually lead to the identification of novel genes involved in biotic and abiotic stress responses.

However, it is unknown whether the genomic potential of the Thai rice resource (TRR) is maximally capable of achieving optimal agricultural performance required to continue to feed the national population, increase its value to local farmers, and increase its commercial output by capitalizing on its status as the planet’s number one rice exporter (Warr, 2008). As the TRR includes hundreds of NFVs, it is difficult to know whether potential genomic treasure troves are being overlooked or whether it is necessary or otherwise desirable to seek additional non-native cultivars in rice development breeding programs.

With respect to this, we developed a bioinformatics pipeline that is able to evaluate haplotype diversity across the TRR in the context of a global rice resource. The International Rice Research Institute (IRRI) is the curator of the ongoing *3,000 Rice Genomes* project (3KRG) (Li et al., 2014). This publicly accessible resource has successfully sequenced more than 3,000 rice accessions from all rice producing regions of the world. In addition, this project features numerous other data, including phenotypic measurements for numerous traits (often for several thousand of the cultivars) in the project. We focused on the critically important phenotype, grain length (GL), an obvious correlate of crop yield. Forty-one key genes have been identified associated with grain size (Li et al., 2018), while at least 189 genes influence yield (Wing et al., 2018), among which several have been found to harbor significantly advantageous haplotypes across the 3KRG (Abbai et al., 2019). We developed a pipeline that first searches a subset of representative accessions from the IRRI database for functional haplotypes within all known annotated rice genes (Kawahara et al., 2013) that are statistically associated with large (and small) GL phenotypes. Subsequently, the pipeline searches within the TRR for these identified haplotypes, specifically comparing associated haplotypes that are found among and between NFVs and ECs. Thus, the pipeline builds a catalog of potentially interesting markers among TRR accessions, with the aim of identifying valuable genomic regions among overlooked accessions that may prove useful in future breeding strategies in developing high yield, stress tolerant cultivars.

Finally, to be of optimal utility for crop breeding programs, it is necessary that a user may interpret catalogued functional haplotype information contextually by evaluating findings with respect to diversity relationships among accessions across investigated panels. Moreover, relatedness reconstruction may permit further analyses such as predictive genetics (Reyes-Herrera et al., 2020). For domesticated rice, it has been established that *Oryza sativa* is likely an amalgam of two distinct lineages hailing from two wild *Oryza* species that largely delineate distinct *indica* and *japonica* variety types (Stein et al., 2018). Thus, disparate genomic architectures may dictate that any identified variants of interest may function differentially according to their biological source. This has relevance to the TRR as the overwhelming majority of rice varieties cultivated in Thailand are of the *indica* ecotype. To facilitate informed evaluation regarding this, the riceExplorer pipeline automatically performs several diversity evaluation analyses. These include an annotated linkage disequilibrium (LD) map across all chromosomes that graphically indicates potential LD relationships of both identified haplotypes of interest juxtaposed against positions of key genes previously identified as functionally impacting the evaluated phenotype, construction of a neighbor-joining phylogenetic tree, structure-type plots evaluating population genomic relationships, and whole-genome and individual chromosome site-frequency spectrum plots. Finally, riceExplorer generates several data files formatted for use with various publicly available software permitting subsequent analyses of diversity relationships.

Our riceExplorer pipeline (available on GitHub) is based on previously published, free-to-use software. It is piped together *via* the Bash programming language with elements of custom-made Python programs that format files between different software applications and perform both quantitative and database search analyses. Thus, it renders an easy-to-understand outputted record along with numerous graphical figures that can be applied to any agricultural focal species (not only rice) with appropriate genotype–phenotype data. Aside from initial stages that require the downloading of publicly available sequence data, our pipeline is straightforward to use, being able to run as a single utility. Importantly, our pipeline represents a financially prudent method by which the user may search their own genomic resources for potentially useful functional variants. This is possible because a full evaluation of a locally held sequenced accession panel can be searched for associated haplotypes without any monetary or logistical investment into large-scale experimental projects requiring phenotypic evaluation. Fundamentally, riceExplorer provides a descriptive database resource that can inform breeding program strategies that may range from traditional breeding methods to modern state-of-the-art genomics-assisted breeding technologies.

## Materials and Methods

### The Thai Rice Resource (TRR)

The TRR comprises hundreds of rice germplasm accessions that have undergone whole-genome sequencing. For this study, we used 279 available sequenced accessions (Supplementary Table S1). Thirty five are elite cultivars (EC) of which several decades of breeding program resources have been devoted to develop lines that provide high yield, good cooking quality, and pest resistance to Thai endemic biotic stressors, and are suitable for use within the main environmental regions within Thailand’s rice-growing areas. A further 190 are local landraces and other varieties developed by the Thai Rice Department. Whole-genome resequencing data were generated using Illumina HiSeq 2,500 System at Novogene (Beijing, China) under the whole-genome resequencing project at the Rice Gene Discovery, National Center for Genetic Engineering and Biotechnology, Thailand (unpublished data). The SNPs were called using the standard GATK pipeline called against the Nipponbare reference genome (Kawahara et al., 2013) to produce SNP calls in the gvcf format. The remaining samples are of external origin sourced from various foreign research agencies (e.g., IRRI).

### Primary Evaluation of Known Grain Length Gene Diversity Within the TRR

It order to initially assess Thai rice resource (TRR) panel genomic diversity with respect to grain length, we used the nucleotide diversity (π; Nei and Li, 1979) metric to compare between the TRR and the IRRI *3000 Rice Genomes* (3KRG) database among the 41 grain length (GL) genes identified by Li et al. (2018) Supplementary Table S2. We sampled all variants within the 41 GL genes among samples on the IRRI database, according to start–stop positions from the Michigan State University Rice Genome Annotation Project (http://rice.uga.edu/). As π is sensitive to sample size, we used rarefied sampling to control for this confounding factor and reported π directly from the 279 sample TRR panel against the distribution of 100 estimates of π from 279 randomly selected accessions from 2,103 accessed IRRI samples.

### Summary of Pipeline Functionality and Implementation Across 55,000 Annotated Rice Gene Regions

The riceExplorer pipeline comprises an initial, semi-automated first step followed by a fully automated analysis when run in a Linux environment (https://github.com/ctdarwell/riceExplorer contains downloadable scripts and comprehensive details about pipeline functionality and implementation). It can be implemented on any agricultural focal species with appropriate genotype–phenotype data whose annotated gene database is incorporated in the snpEff software suite (Cingolani et al., 2012).

The first step is to decide an appropriate reference library for initial inquiry. For our TRR, the IRRI 3KRG project (Li et al., 2014) is appropriate, although the pipeline can be applied to any global (or other) database that provides both whole-genome sequenced material (gvcf format) and associated phenotypic values for individual accessions. Despite the availability of more than 3,000 sequenced accessions, 3KRG recommends (for obvious reasons of tractability and computing constraints) that a core set of 72 accession that best represent global rice diversity is primarily used for analyses. However, despite this recommendation, the 72-accession core panel does not have a complete set of associated phenotypic assays that are also curated under 3KRG (see: https://snp-seek.irri.org/_variety.zul). Of the 72 core accessions, only 49 have data for grain length (GL), the trait which we focus on in this paper.

For this initial step, we have therefore developed the *sampleSelector.py* script (available on the GitHub pages). As input, it takes the full available GL data from the 3KRG pages (here, accession name and phenotypic assay of 2,103 samples). In order to maximize phenotypic diversity and genetic representation among the reference library, the program identifies the held accessions that have maximal and minimum phenotypic values both within all designated regions (here: South Asia, East Asia, Southeast Asia, Africa, Indo-Pacific, Europe and the Middle East combined, the Americas, and Australasia) and rice variety types (designated as: *indica*, *japonica*, *aus*, *aromatic*, *admix*). Consequently, for our analysis, *sampleSelector.py* indicated a further 151 varieties’ gvcf files to download from 3KRG (Supplementary Table S3), alongside the 49 samples from the recommended core accessions that have associated GL data (i.e., 200 files; e.g., https://3kricegenome.s3.amazonaws.com/Nipponbare/IRIS_XXX-XXXXX.snp.vcf.gz). From this, a data file featuring accession name and associated GL phenotype can be compiled for input to the main pipeline.

The main pipeline requires five key data elements: a downloaded reference library (in gvcf format), associated phenotypic measurements of the reference library, a sequenced focal library (i.e., of breeding program data, e.g., TRR; gvcf format), an associated population file describing sample variety types, and a list of genomic regions of interest. For this last item, we use the Michigan State University rice annotation project list of *ca*. 55,000 annotated genes (http://rice.plantbiology.msu.edu/pub/data/Eukaryotic_Projects/o_sativa/annotation_dbs/pseudomolecules/version_7.0/; as both our reference and focal sequence libraries’ SNP calls derive from the Nipponbare rice reference genome). In addition, the pipeline can accept a list of annotated genes known to have primary function associated with the focal trait, in order to annotate the final graphical output. For this, we included the 41 genes identified by Li et al. (Li et al., 2018) as know grain length genes.

**Figure 1:**
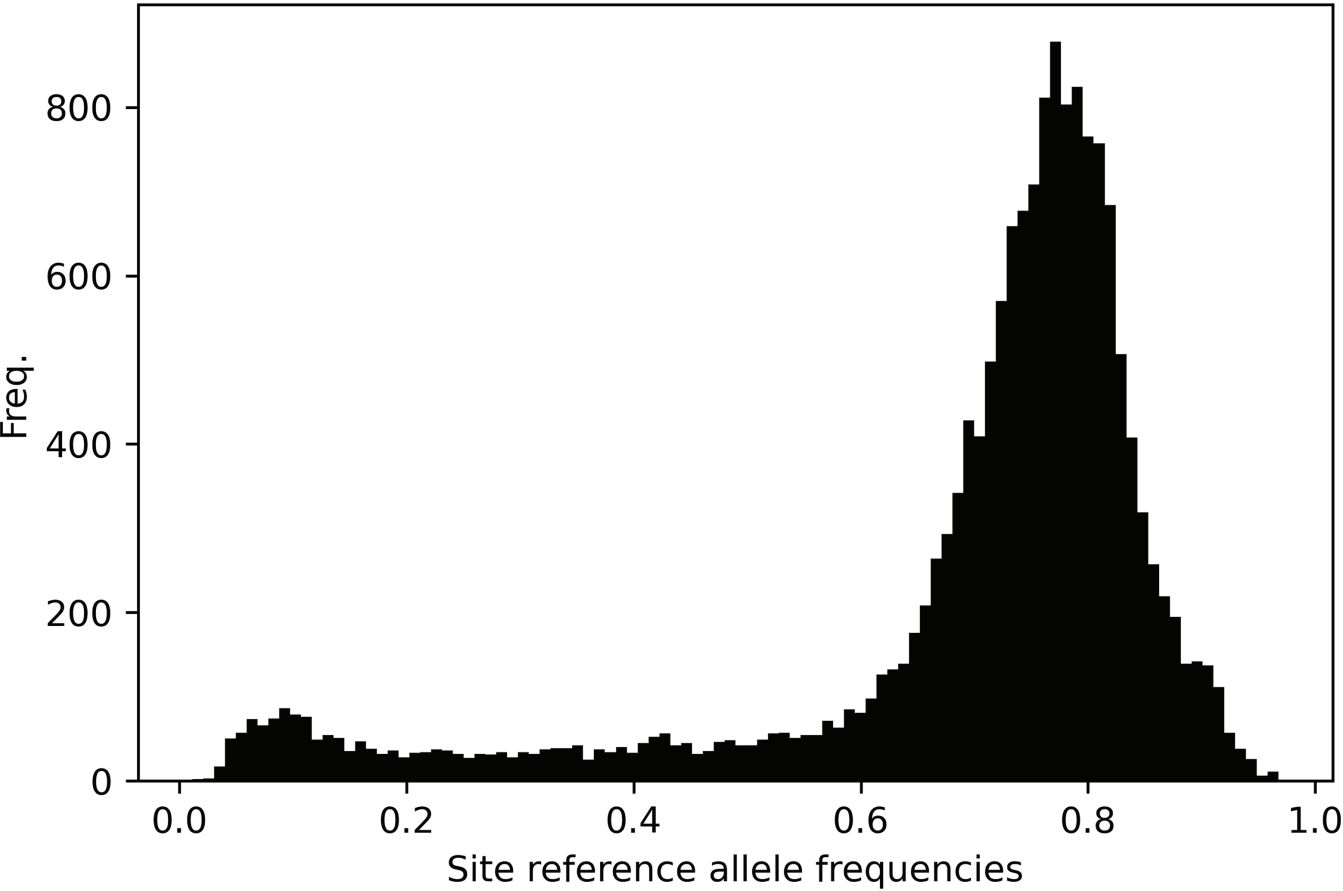
Flowchart representation of workflow performed by the riceExplorer bioinformatics pipeline. Orange filled boxes represent data inputs; black bordered boxes represent publicly available, previously published software integrated into the workflow; blue bordered boxes represent custom-made Python programs developed for this pipeline; red bordered diamonds represent program/workflow outputs; red-filled box indicates final outputs *via* scripts developed to automate figure production; dashed green arrows indicate continued flow from bottom to top.

Pipeline implementation is as follows. First, the bcftools software suite (Danecek et al., 2021) is used to call all variants from our grain length reference library (i.e., 3KRG). Next, a Python script (*vcf4snpeff.py*) reformats the bcftools output ready for the next step. For all generated files containing SNP information, we use the snpEff software suite (Cingolani et al., 2012) to evaluate the functional impact of all identified SNP calls. Our pipeline then uses the bash ‘grep’ command to select only SNPs called as moderate or high by snpEff—these SNPs being identified as having a likely functional impact on any biological function of the organism. Figure 1 outlines the basic pipeline workflow.

The next pipeline process uses outputted snpEff information indicating predicted functional impacts of all evaluated SNPs to build *functional haplotypes* for each examined annotated gene. For each gene, a Python script (*hapXphenoPredictorSNPs.py*) reconstructs the functional haplotype occurring in each accession. For each accession, the program builds the haplotype according to whether the reference or alternative allele is present at each identified functional SNP location. For example, if an annotated gene region contains four functional SNPs, different functional haplotypes are considered that contain any combination (i.e., reference or alternative) of those four SNPs (and featuring a minimum of one functional alternative allele relative to the Nipponbare reference genome) within that gene for each accession. For each reconstructed haplotype, the mean associated phenotype is calculated across all 3KRG individual accessions carrying that haplotype. This is done because the large number of possible haplotype alleles (that have no scalable magnitudes) means it is not possible to conduct a meaningful analysis against individual phenotype scores (*cf*. GWAS which exploits bi-allelic SNP collapsibility to binary values to implement regression analyses). If the mean associated phenotype for those accessions (and the haplotype is found across a minimum of five accessions) is greater or less than (both are recorded in case the optimal phenotype is at lower values) the user-stipulated percentile range from the distribution of the phenotypic data, that haplotype is recorded as being associated with extreme phenotypes (here our interest lies with *large grain associated haplotypes*; hereafter LGHs) within the reference library panel. For our analysis, we chose a percentile value of 0.866 which represents 1.5 standard deviations from the mean (NB while the user may select a percentile value of 0.975 to represent full significance, it represents an unreasonable value because the pipeline evaluates mean values across accessions for each identified haplotype—thus, it would be highly unlikely to yield such extreme outlier haplotypes).

The final pipeline step then searches the focal (i.e., breeding program) sequence data (using bcftools) to: (i) identify presence/absence of the identified haplotypes, and (ii) evaluate whether that haplotype is present/absent among elite cultivars (ECs), or more pertinently, non-focal varieties (NFVs) in the breeding program panel using the *potential_rgd_sourcesLoop.py* Python script. Finally, the pipeline outputs a number of summary tables and annotated graphical outputs according to evaluated chromosomes in the analysis.

### Putative Function of Identified Genes

To assess the most commonly identified gene functions identified by our pipeline, we converted all identified MSU annotated genes into their GOSlim assignments (http://rice.uga.edu/downloads_gad.shtml). To easily visualize common functional types, we removed common technical terms from the GOSlim putative function assessments and performed word cloud analyses based on number of hits using the Python library “*wordcloud*” (https://pypi.org/project/wordcloud/). Full results are also tabulated.

### Auxiliary Analyses of Relationships Among Accessions

In order that the user may evaluate the potential value of identified LGHs, the riceExplorer pipeline performs a number of subsequent analyses to evaluate diversity relationships between accessions and linkage disequilibrium patterns within chromosomes across both the reference and focal sequencing libraries. Additionally, the pipeline also outputs formatted files that may be used as input for more robust phylogenetic and demographic analyses. Alongside publicly available software, an additional 12 custom Python scripts are included with the GitHub pages for these analyses (described below).

### SNP Selection

To minimize computation time, the pipeline first identifies SNPs with high coverage across accessions. We assume that the employed reference library is of high quality and therefore coverage across the focal library is evaluated. A single SNP with the highest coverage that passes several quality criteria (minimum coverage across accessions >80%; minimum allele frequency < 5%; maximum of two alleles per site; variants recorded as low quality are rejected) is selected from each examined gene region. SNPs across accessions are then called using Bcftools. vcf files are compiled for individual chromosomes and then also merged for a whole genome summary. Across the genome from our investigated libraries, a total of 16,596 SNPs were recovered that passed the quality control criteria.

### Linkage Disequilibrium Relationships

First, the R packages LDheatmap (Shin et al., 2006) and snpStats (Clayton and Clayton, 2012) are used to calculate linkage disequilibrium (LD) between recovered SNPs for each of the generated individual chromosome vcf files. As *indica* is by far the favored rice variety type in Thailand and our focal panel consists of 257 out 279 cultivars, LD was only calculated among *indica* accessions to (a) provide appropriate analyses, and (b) to avoid incorporating distinct and potentially confusing LD relationships likely to be found among different variety types. From the resultant matrices, a custom Python script then plots the LD heat maps. However, this script also incorporates the LGH data generated from the primary pipeline in order to annotate the LD plots so that identified LGHs are positionally marked according to the chromosomal location. Graphical outputs are generated for all chromosomes with individual plots generated according to whether LGHs are high or low extreme values or whether they have been identified within elite cultivars or non-focal varieties, and their positions relative to previously identified genes of interest.

### Phylogenetic Relationships

First the pipeline constructs a neighbor-joining tree. The whole genome vcf is converted into Fasta format retaining the reference/alternative allele base calls. Next, custom Python scripts calculate pairwise Kimura-2-parameter (K2P) distances (Kimura, 1980) between all accessions. This matrix is then converted into a dendrogram using Ward’s clustering method (Ward, 1963) and subsequently converted into Nexus tree format for visualization in external tree-viewing software. A graphical output of the dendrogram and an accompanying data file is also generated indicating Ward’s assessment of cluster number.

### Population Genomic Analyses

riceExplorer employs the sNMF software package (Frichot et al., 2014) to infer individual admixture coefficients and generate population structure graphical output from the previously generated whole genome vcf. After initial analyses, a custom Python script employs the cross-entropy evaluation method of Evanno et al. (2005) which is employed to establish the most likely number of distinct genetic demes (populations; K) present in the panel. For this, a clear value of K can be intimated if a single cross-entropy scores is notably lower than for other estimates of K. Finally, a further script generates a cluster plot for the evaluated K value. IRRI metadata assessment indicates four cultivar variety types: *aromatic*, *aus*, *indica*, *japonica*, as well as a variety identified as *admix*.

### Site-Frequency Spectrum Plots and Additional Outputs

riceExplorer also employs custom Python scripts to output site-frequency spectrum plots both within chromosomes, using individual chromosome vcf files, and a plot of frequency distributions across the entire genome. Additionally, custom Python scripts are also used to generate input files formatted for use with the Snapp (Bryant et al., 2012) and diy abc (Cornuet et al., 2008) programs in order to perform more robust phylogenetic and demographic analyses.

## Results

### Genomic Diversity Across Key Grain Length Genes

**Figure 2:**
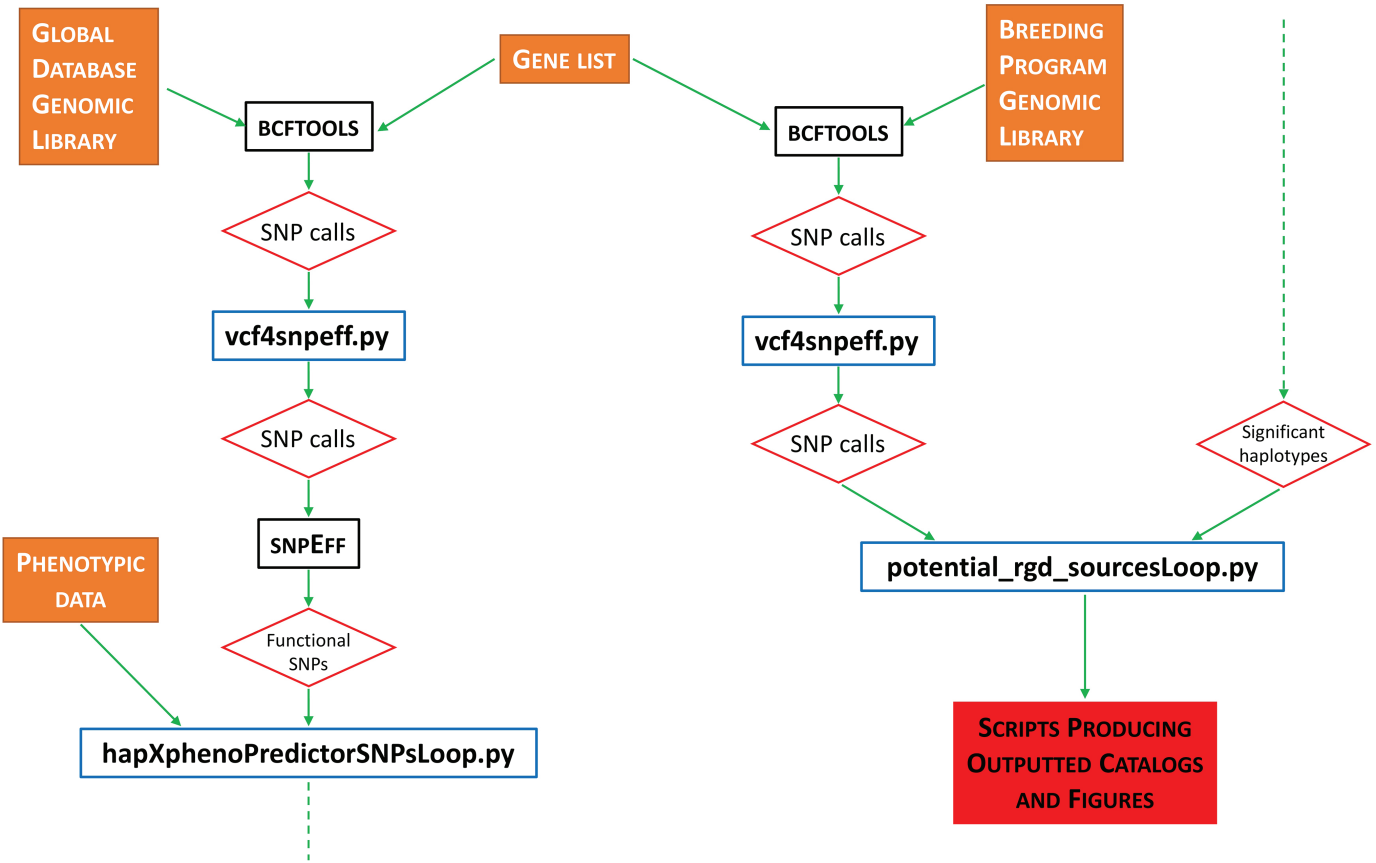
Boxplots comparing nucleotide diversity (π) at 41 genes across the rice genome known to exert functional influence on rice grain size. Red dots indicate π across 279 Thai accessions, while boxplots indicate distributions of 100 subsampled (n = 279) estimates of π across 3KRG samples. Thai accessions have significantly reduced π at 28 gene regions compared with global diversity at these loci, while four genes show significantly elevated π among Thai accessions compared with the global database. See Supplementary Table S1 for full information regarding GL genes.

Twenty-eight out of the 41 genes previously identified (Li et al., 2018) as having direct functional influence on rice grain size show reduced nucleotide diversity across the Thai rice resource (TRR) germplasm panel when compared with the *IRRI 3000 Rice Genomes* (3KRG) global database (Figure 2). Additionally, four genes show increased nucleotide diversity in Thailand when compared with the *IRRI* samples. This indicates that the TRR has limited potential to improve grain size at the majority of key grain length loci.

### Identification of Large Grain Length Haplotypes (LGHs) Across the TRR

After investigating the 3KRG database to identify extreme grain size-associated haplotypes, our riceExplorer bioinformatics pipeline identified 8,983,562 SNPs of which 906,899 across 28,303 annotated genes were functionally impactful. At the 1.5 standard deviation threshold, these generated large grain size-associated haplotypes (LGHs) at 295 MSU annotated genes across all accession types (Table 1). Among Thai landraces and Rice Department varieties (i.e., non-focal varieties; NFVs), 268 LGHs were identified, of which 47 are unique to these accession types. Moreover, 114 LGHs are found among NFVs that are not found among core TRR elite breeding line accessions (ECs). Chromosomes 1–4 and 6–8 feature the most LGHs, while chromosomes 9–12 are notably sparse. Additionally, the 35 EC accessions provided 159 annotated LGH genes (4.54 per accession), while 190 NFV accessions yielding 295 genes equates to 1.41 per accession.

**Table 1:** Large grain size associated haplotypes (LGHs) distributions across the chromosomes of the Thai rice panel. Column names indicate: “Total”—LGHs identified across all accessions; “nNFV”—number identified among NFVs; “nNFV unique”—number identified uniquely among NFVs; “nNFV unique (EC)”—number identified among NFVs but not among ECs.

Chromosome	Total	nNFV	nNFV unique	nNFV unique (EC)
Chromosome 1	31	25	4	14
Chromosome 2	27	25	5	11
Chromosome 3	46	43	6	16
Chromosome 4	36	34	6	13
Chromosome 5	17	15	0	5
Chromosome 6	22	19	3	8
Chromosome 7	42	38	14	23
Chromosome 8	31	30	2	9
Chromosome 9	9	7	0	3
Chromosome 10	7	7	2	3
Chromosome 11	13	12	2	6
Chromosome 12	14	13	3	3
Total	295	268	47	114

**Figure 3:**
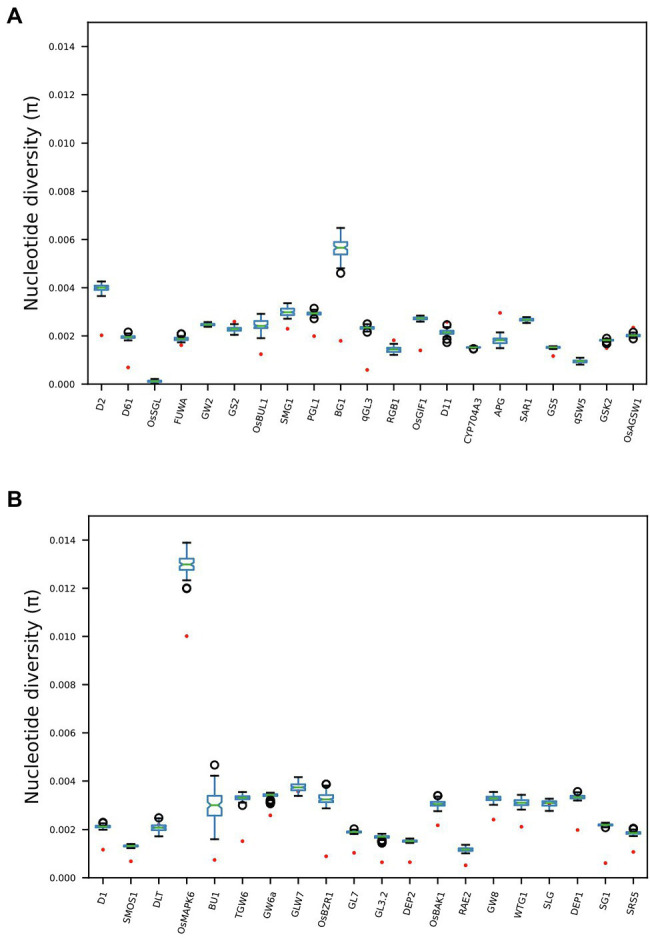
Map of chromosome 7 showing identified haplotypes associated with large and small grain sizes identified in a global database found across our TRR panel. RD varieties and landraces (NFVs)—blue chromosome; RGD targeted breeding accessions (ECs) – green chromosome. Haplotypes associated with large grain size are indicated above each chromosome whilst haplotypes associated with small grain size are indicated below. The known grain size gene positions for *LOC_Os07g32170*, *LOC_Os07g39220*, LOC_Os07g41200*, LOC_Os07g41240* and LOC_Os07g42410 are indicated with orange squares (* adjacent genes identified by the same square). Gene annotation names in blue text are found among both NFV and EC accessions.

For each chromosome, our riceExplorer pipeline outputs graphical maps indicating the location of both LGHs and small grain size-associated haplotypes across the chromosomal regions of both NFV and EC accessions (Supplementary Figure S1). Inspection of these outputs indicate repeated occasions where NFV chromosomal regions display clusters of LGHs in which the corresponding EC regions exhibit a dearth or absence of such loci (e.g., chromosomes 1–3, 6 and 7). For example, the chromosomal region between 12.65–26.47 Mb on chromosome 7 features 27 LGHs (Figure 3). Notably, these are found on a chromosomal region encompassing the five known grain length genes on this chromosome (Li et al., 2018). EC accessions have only six LGHs on this region.

### Potential Origins of LGHs Across the TRR

**Figure 4:**
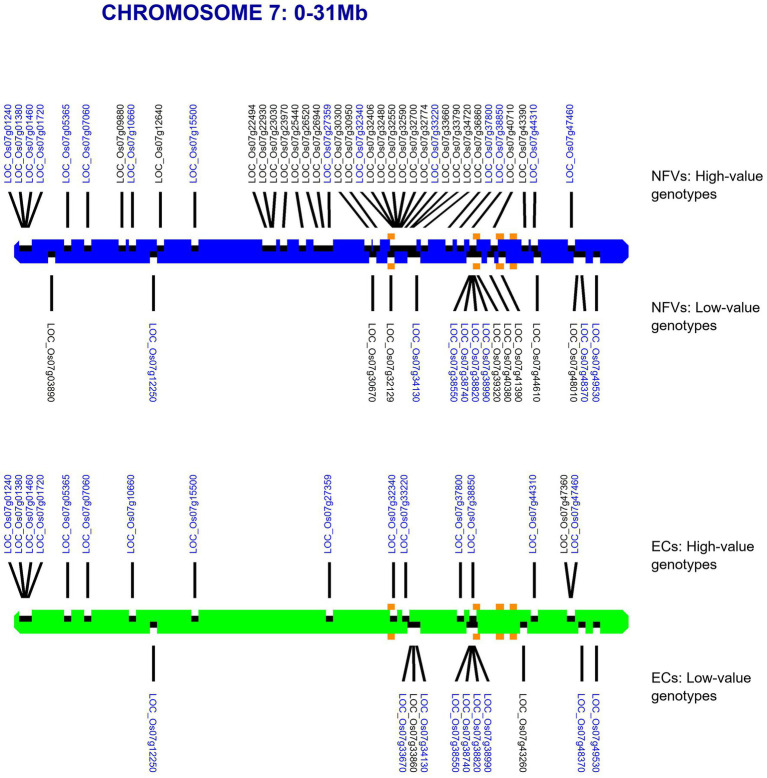
Likely origins of identified large grain size haplotypes on Chromosome 7. Proportions indicate geographic origins of accessions in the 3KRG database, according to rice variety group, of novel identified haplotypes (i.e., LGHs identified in 3KRG varieties). Numbers adjacent to segments indicate number of haplotypes per accession (i.e., to control sampling bias) from that region. NB Sundaland indicates the Indo-Pacific region; sub-continent indicates South Asia region.

**Figure 5:**
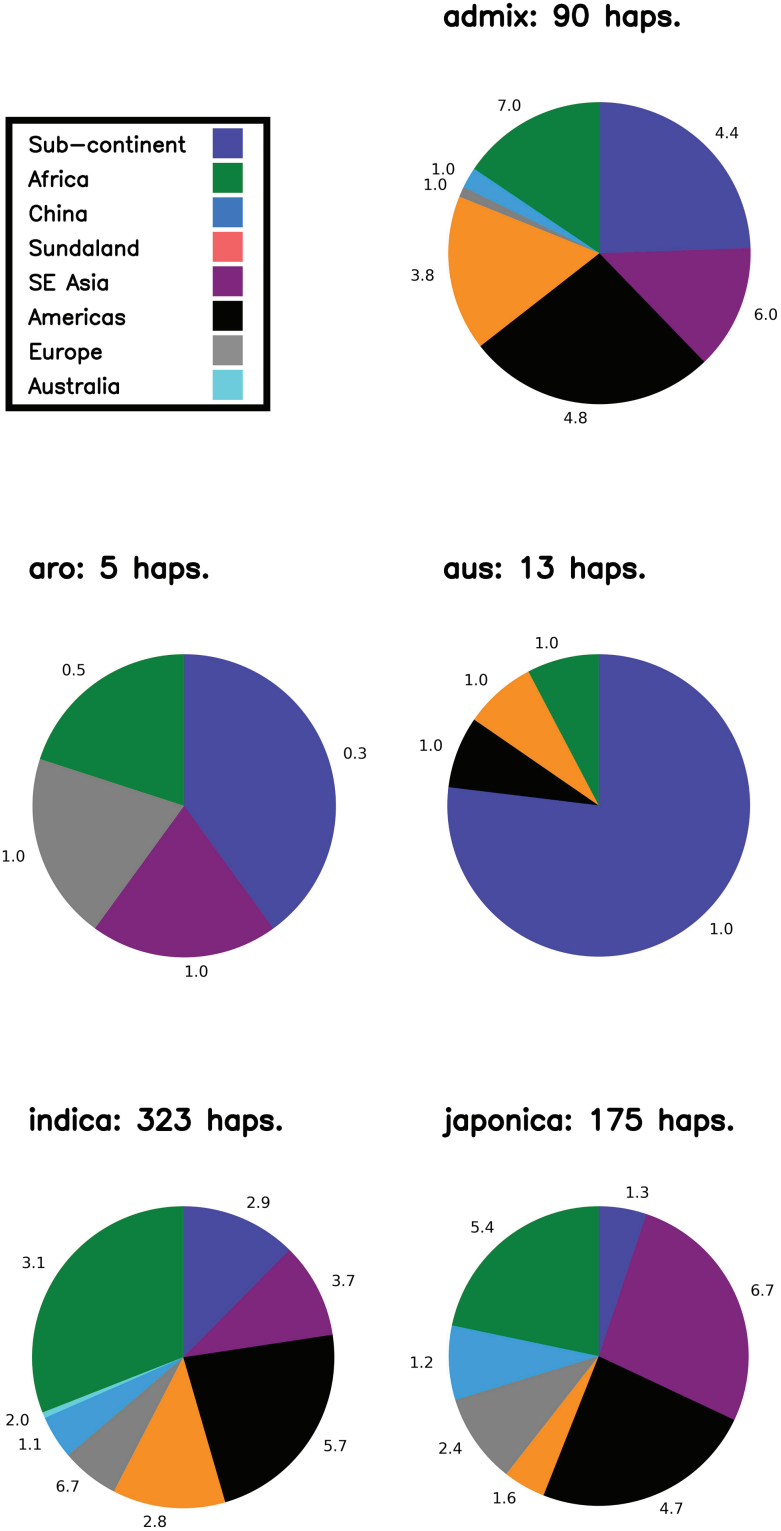
Word cloud indicating most prominent terms from MSU GOSlim Assignment putative functions of LGHs among Thai NFVs. After removing technical terms, the most prominent gene functionality types include cytochrome P450 domains, auxin proteins, phosphate dehydrogenases, glutamate synthesis proteins, and chalcone synthases. See Supplementary Table S3 for full list.

In order to: (i) understand the distribution of LGHs found within NFVs: (ii) further understand the origins of genomic diversity among Thai rice; and (iii) catalog genomic diversity among Thai rice, our bioinformatics pipeline outputs a summary figure that breaks down potential geographic haplotype origins according to rice variety group (i.e., *indica*, *japonica*, *aromatic*, *aus*, *admixed*; Figure 4) across each chromosome. First, focusing on LGHs across chromosome 7, the majority of haplotype records (n = 323) are found among *indica* varieties within the 3KRG database, with a large contribution of samples from African and the Americas, contributed at 3.1 and 5.7 haplotypes per accession, respectively. However, controlling for sample bias, and despite contributing around 10% of haplotypes, European samples contribute the most (6.7) haplotypes per sampled accession on chromosome 7. The Americas and Southeast Asian samples contributed the most to *japonica* LGHs (around 50%) at 6.7 and 4.7 haplotypes per sampled accession, respectively. The Americas and the Subcontinent contribute most to admix LGHs are (4.8 and 4.4 haplotypes per sampled accession, respectively), while African samples contributed most (7.0) per accession. Few LGHs were identified on *aus* and *aro* 3KRG samples. This pattern on chromosome 7 of mostly *indica* LGH contributions is typical across all other chromosomes (Supplementary Figure S2). Finally, a cursory word cloud inspection of MSU gene annotation descriptions for all LGH loci indicates a subset of prominent gene functionality types that riceExplorer identified (Figure 5; Supplementary Table S4).

### Investigation of Individual Accessions

**Figure 6:**
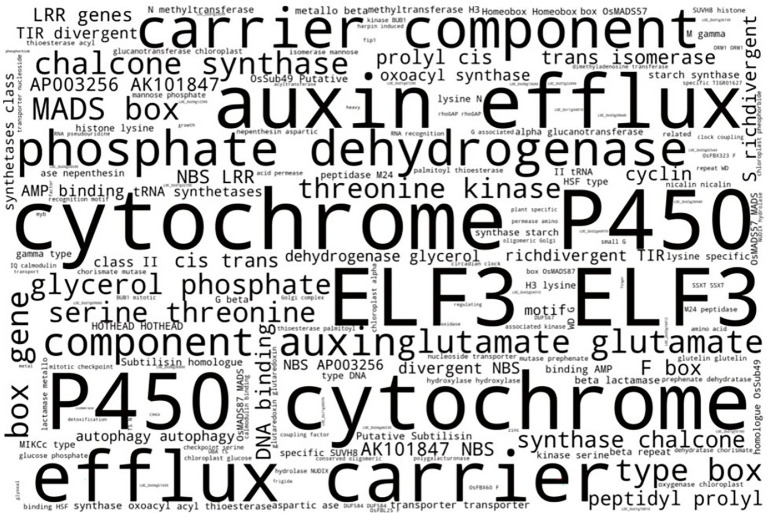
Map of chromosome 7 (0–31.0 Mb) showing all identified LGHs across elite cultivar accessions and LGHs identified on Thai landrace *Ai-Tai* (W00223). Orange square markers show LGHs found on the landrace accession Ai-Tai, while gene positions for known grain length genes *LOC_Os07g32170*, *LOC_Os07g39220*, *LOC_Os07g41200*, *LOC_Os07g41240, and LOC_Os07g42410* are indicated with blue-shaded squares. *Ai-Tai* features a cluster of 14 LGHs all between chromosomal region 17.9–24.4 Mb.

Our pipeline analyses indicate that particular Thai NFV cultivars feature high numbers of LGHs across all 12 rice chromosomes (Table 2). For example, the Rice Department accession *Ai-Tai* (code W00223) has 15 LGHs on chromosome 7 (Figure 6) as well 25 LGHs across other chromosomes. It warrants particular attention because its LGHs are clustered around 17.9–24.4, surrounding the region containing five known grain length genes and which possesses a sparse record of LGHs among EC accessions. It is also noteworthy that Thai EC accessions contain seven small grain length associated haplotypes within the 20.1–23.4 Mb region. Our software can output figures for specified individual samples in order to view their potential contribution to agricultural breeding programs.

**Table 2:** Table of individual accessions featuring more than ten large grain size-associated haplotypes (LGHs) found among Thai NFV accessions but not ECs across all 12 rice genome chromosomes. See Supplementary Table S5 for full list.

Thai accession	GS No.	Type	nLGHs
W00269	24841	Landrace	37
W00167	14142	Landrace	33
W00132	24601	RD variety	29
W00223	8100	Landrace	29
W00237	24613	Landrace	26
W00258	23725	Landrace	16
W00306	8113	Landrace	13
W00082	23179	Landrace	12
W00319	14331	Landrace	12
W00159	–	Landrace	10

### Linkage Disequilibrium Relationships

**Figure 7:**
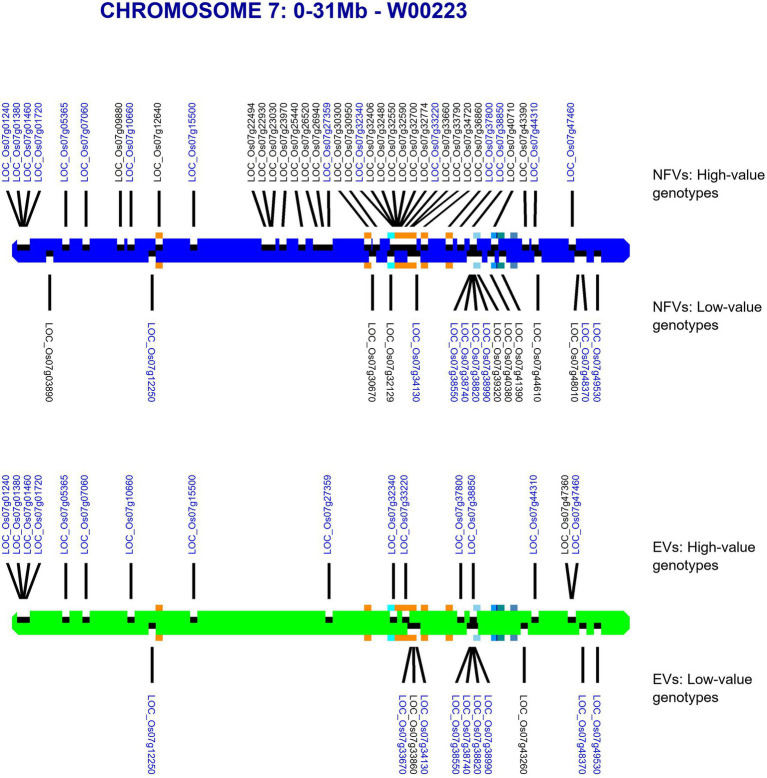
Annotated linkage disequilibrium map of chromosome 7 (0–28 Mb). MSU gene annotations of LGHs identified by riceExplorer are positionally marked (red) alongside genes with known influence on grain length (black) that were initially imported to the program by the user.

Figure 7 indicates linkage disequilibrium (LD) relationships across chromosome 7 (Supplementary Figure S3 for details regarding other chromosomes). Notably, there are regions of high linkage (12.65–20.22 and 25.98–28.37 Mb) surrounding the clusters of LGHs among NFVs identified by riceExplorer (Figure 3), as well as the four previously identified genes associated with grain length (Li et al., 2018). Further, these highlighted regions of high LD are shown to have a relatively low number of LGHs among ECs. Similarly interesting regions are apparent on chromosomes 1, 3 and 6 (Supplementary Figures S3A,C,F). riceExplorer further outputs tables referencing all pairwise LD estimates between both identified LGHs and any inputted previously known genes. For example, on chromosome 7, the LGH loci, LOC_Os07g23970 and LOC_Os07g22930 display linkage greater than 0.8 with locus LOC_Os07g22494.

### Phylogenetic Relationships Among Accessions

From the combined libraries, 454 samples passed quality control criteria and are included in subsequent analyses. A neighbor-joining tree generated using the K2P distance metric indicates that virtually all *indica* variety types, from both the reference (IRRI) and focal (TRR) panels, cluster together in a monophyletic clade, with IRRI *aus* and *adm* biotypes forming sister clades (clade I; Supplementary Figure S4). Most *japonica* accessions are in the distinct major clade II from *indica* biotypes, although they display paraphyletic relationships. However, the single RGD *japonica* accession that passed quality control criteria was placed among IRRI ADM samples among a sister clade of *indica* samples in clade I.

### Population Genomic Analyses

**Figure 8:**
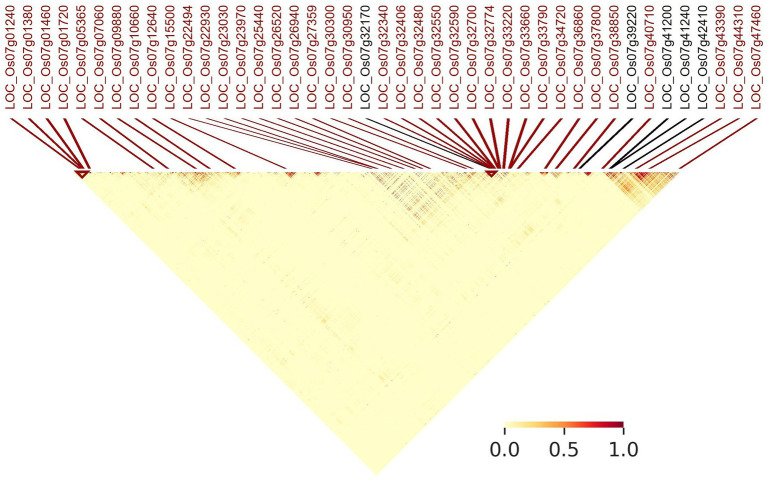
Population genomic structure plot following analyses in sNMF software indicating relationships patterns at the four-deme level. There is clear delineation according to accession variety types although further variation appears according to library origin of *indica* samples.

Cross-entropy analyses indicate no obvious number of demes among our combined libraries although the lowest score indicates 13 populations (Supplementary Figure S5). This is not unreasonable as IRRI metadata indicates 15 distinct subpopulations among five variety types. However, for our purposes, we consider the breakdown of relationships for K = 4 (following IRRI metadata assessment of variety type number; Figure 8). Among IRRI samples, there appears to be three distinct populations: *aus* and *indica* largely forming the same deme, japonica, and admix (identified as featuring mixed demes). However, in contrast to phylogenetic findings (Supplementary Figure S4), RGD *indica* do not cluster with IRRI *indica*, while the single RGD *japonica* sample does not cluster with IRRI *japonica*. Further evaluation of our genome wide vcf file indicates only a 4% differential in missing data between IRRI and RGD samples suggesting unequal sampling is not the reason for the disparity. This may be a result of large data artifact between different NGS sequencing protocols used in generating the two libraries.

### Site-Frequency Spectrum Analyses

**Figure 9:**
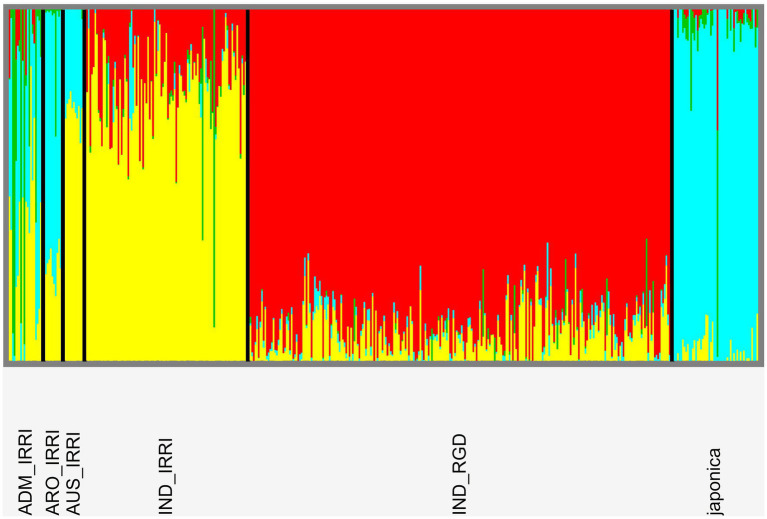
Site-frequency spectrum plot across all 12 rice chromosomes. The data are left-skewed as a result of SNP calling a predominantly *indica* panel against a *japonica* reference genome.

Figure 9 shows the site-frequency spectrum across all chromosomes. Data are skewed indicating a predominance of alternative alleles. This pattern is ubiquitous across individual chromosomes (Supplementary Figures S6). This no doubt reflects that our data matrix solely contains SNP loci from only gene coding regions and predominantly *indica* cultivars called against a *japonica* reference genome.

## Discussion

Agricultural crop breeding programs, potentially conducted at the national level, may primarily focus on the performance and development of elite focal cultivars (ECs). Attention will normally focus on development and improvement of ECs in the forms of marker-assisted selection, gene-editing and other forms of selective breeding that may have encouraged powerful directional selection. Such anthropologically mediated evolutionary forces may induce a plethora of incidental outcomes that encourage the accrual of non-lethal deleterious mutations across nontarget regions of the genome (Moyers et al., 2018). This has the potential to cause a weakened genetic background due to pleiotropy (Darmency, 2013; Paaby and Rockman, 2013) or other mechanistic processes. Furthermore, despite their utility in providing statistical power to large-scale genome-wide experimental studies, the potential utility of non-focal varieties (nominally landraces and other locally endemic varieties; NFVs) may have been unwittingly neglected (Tokatlidis and Vlachostergios, 2016). Additionally, typically employed SNP-based methods have potential pitfalls emanating from nonaccounting of epistatic dynamics (Clark, 2004; Bardel et al., 2005), low informativeness (Hamblin and Jannink, 2011), and disproportionate influence of rare variants on extreme phenotypes (Wray et al., 2013). Our riceExplorer bioinformatics pipeline offers a powerful, low-cost, and easily employed tool to both explore the comparative genomic variation among NFV accessions (relative to EC or alternatively stipulated accessions) and provide a useful catalogued record of their untapped potential genomic contribution.

We evaluated the distribution of all haplotypes comprising SNPs predicted to have functional impact (predicted by snpEff software) (Cingolani et al., 2012), and associated with extreme grain length phenotypes, across all *ca*. 55,000 MSU annotated gene regions (Kawahara et al., 2013) against our panel of Thai rice resource (TRR) accessions using our newly developed pipeline. In particular, we were interested in evaluating whether there are significant numbers of LGHs among NFVs that are not found among EC accessions. The 35 ECs yielded 159 LGHs (4.54 per accession) at almost three times the rate as was found among the 190 NFVs (295 LGHs at 1.41 per accession). This suggests that the overall impact of elite cultivar breeding programs featuring sophisticated technologies and extreme selective regimes does not have a disproportionately negative impact on nontarget genomic regions among ECs. Although without comprehensive pedigree analyses of the accession panel, it is impossible to say that Thai breeding programs have had no deleterious impacts.

However, our results indicate that NFVs contain numerous functionally impactful haplotypes that significantly associate with high grain length phenotypes in the 3KRG accession panel which are not found among EC accessions. Thus, indicating that ongoing breeding programs and focus on ECs may have resulted in the breeding potential of NFVs being overlooked. Many LGHs can be found clustered across significant lengths of several chromosomal regions in which EC accessions do not feature any LGHs (e.g., chromosomes 1–4, 6, 8; Supplementary Figure S1). Our results highlight chromosome 7 (Figure 3), where chromosomal region 12.65–26.47 Mb contains 27 LGHs located where there are only six recorded among ECs and where ECs contain several small grain length associated haplotypes. Many of these lie adjacent to known grain length genes (Li et al., 2018) identified as having reduced nucleotide diversity on chromosome 7 (*LOC_ Os07g39220*, *LOC_Os07g41200*, *LOC_Os07g41240* and *LOC_Os07g42410*; Figure 2), potentially in strong linkage disequilibrium. Furthermore, investigation of the output files generated by riceExplorer indicate that the landrace accession *Ai-Tai* (W00223) contains 15 LGHs in this region (Figure 6). Investigation of the output files from riceExplorer identified numerous examples of this type from our accession panel across the rice genome (Supplementary Table S5).

We also investigated the likely origins or affiliations of LGHs found among NFVs. For continuity, we initially interpret results on chromosome 7 (Figure 4). The majority of LGHs originally identified as associated with large grain size in the 3KRG database and also identified among TRR NFVs are found among IRRI *indica* varieties (n = 323) for this chromosome. This is unsurprising as most of the TRR panel comprises *indica* varieties that is the favored staple rice type in Thailand. Most of these (> 50%) originate from varieties from Africa and the Americas in the 3KRG panel. However, our pipeline also controls for sample size and evaluates haplotype sources according to the contribution of the number of accessions in the original reference (i.e., 3KRG) panel. When controlling for this sampling bias, we show that European accessions (which provided only 6% of IRRI accessions) associate the most with LGHs (6.7) per accession for *indica* on chromosome 7. This result is likely sensible. If the Thai breeding program has mostly focused on endemic accessions and others from the nearby regions (e.g., China and other Southeast Asian countries), we may expect that novel LGHs are likely to disproportionately come from other regional accession panels. Thus, future research within Thailand may consider incorporating more European *indica* accessions into the program in the hope that more useful gene regions that are compatible with the TRR may be identified. Such reasoning may prove fruitful, as theory dictates that hybrid vigor may yield more useful phenotypes (Birchler et al., 2006).

Finally, riceExplorer conducts a cursory word cloud inspection of MSU gene annotation descriptions for all LGH loci found among NFVs in order to evaluate which kinds of genes are most commonly identified and may also offer insight as to where future research may be directed (Figure 5). A subset of prominent gene functionality types was identified, including cytochrome P450 domains, auxin proteins, phosphate dehydrogenases, glutamate synthesis proteins, chalcone synthases, and others. It may be prudent to search for such gene family types, to incorporate into EC genomes during future research.

riceExplorer auxiliary analyses of relationships among panel accessions permits further evaluation of potential utility of identified LGHs. Annotated linkage disequilibrium plots inform the user of chromosome-wide linkage patterns relative to identified markers alongside those manually inputted as known phenotypically relevant genes (Figure 7). This information is augmented by tabular outputs highlighting specific relationships between markers of interest. Additionally, our riceExplorer evaluates phylogenetic (Supplementary Figure S4) and population genomic (Figure 8) relationships among accessions. For our panel, it is evident that that there are two distantly related clades dominated by *indica* and *japonica* variety types within rice (Stein et al., 2018). Our population genomic analyses further separated IRRI and TRR indica accessions, potentially due to data artifacts between different NGS sequencing protocols when generating the two libraries. Inspection of additional riceExplorer output files will inform the user if identified LGHs are found among potentially compatible cultivars relative to the program’s elite cultivar (EC) panel. Further outputs allow evaluation of site-frequency spectrum (SFS) patterns (Figure 9) across chromosomes. For our panel, featuring predominantly *indica* varieties with variants called against a *japonica* reference genome, SFS plots are notably left-skewed.

### Implications for Breeding Program Guidance

In general, riceExplorer identifies LGHs among a large number of annotated gene regions. Most of these are unlikely to be directly linked to the study phenotype, in this case, grain length, although recent advances indicate that markers contributing to complex phenotypes are typically numerous, scattered widely across the genome, and consequently, often uncharacterized (Wing et al., 2018). There are sound theoretical reasons to expect that many of the identified LGHs that are not directly associated with grain length loci may cause improved phenotypic syndromes in TRR accessions. For example, it is likely that the “costs of domestication” such as the accrual of slightly deleterious mutations (Moyers et al., 2018) has occurred throughout the timespan of TRR breeding programs. This may have occurred *via* genetic hitchhiking of linked loci as desirable genotypes have been incorporated into Thai ECs (Moyers et al., 2018). Moreover, many of the identified LGHs may have pleiotropic influence on alleles that directly control grain length. However, before embarking upon any subsequent recuperative breeding strategies, it is also necessary to ensure that none of these identified regions carry other important functional genes, including those that may exhibit negative pleiotropy (Rose, 1982) against large grain length phenotypes.

Cutting-edge technologies employed by modern breeding programs typically involve marker-assisted selection that is moving toward greater reliance on predictive genetics-based breeding (Keller et al., 2020), requiring understanding of species’ responses to abiotic (or other) drivers (Cortés et al., 2020). However, a reliance on SNP markers has its drawbacks and the identification of functional haplotypes has been posited as a powerful additional tool in the genomics-assisted breeding armory (Bhat et al., 2021). As such, riceExplorer will be of great value in directing researchers to regions of the genome that may be ripe for exploitation. riceExplorer’s design functionality means it accounts for several problems associated with SNP-based methods by encompassing epistatic effects, only focusing on functional variants, and by minimizing the influence of rare alleles. However, further investigation would be required to establish whether identified genes display robust predictive signal with respect to key sustainability challenges such as those envisaged by future climate change dynamics. Moreover, its ability to discern haplotype origins according to population origin (e.g., landraces) may aid predictive breeding investigations (as shown for rice; Bhandari et al., 2019) that potentially offer powerful solutions for toward the goals of sustainable agriculture (Blair and Izquierdo, 2012; Cortés et al., 2020).

We expect that our pipeline has provided a starting point to various avenues of research that may lead to the incorporation of available genomic resources into TRR ECs. This may improve the genetic background of future varieties developed to exhibit robust, high-yield stress tolerant phenotypes. With respect to our own dataset, it appears that chromosome 7, at least, including specific genes identified by the pipeline, should be considered as an area of potential improvement within the TRR. Finally, while we present riceExplorer as the full implementable package, we note that it also offers alternative related utility. For example, the implementation of the initial steps 1–4 (see Figure 1) against a sequenced and phenotyped germplasm panel would facilitate the cataloging of functional haplotypes associating with extreme phenotypes (while evaluation of the full snpEff outputs would also include identification of non-functional haplotypes—see GitHub pages for details).

## Conclusion

Agricultural crop breeding programs are likely to (i) exaggerate deleterious alleles at nontarget loci among focal elite cultivars (ECs), (ii) overlook the potential genomic resources residing in their non-focal variety (NFV) accessions, and (iii) over-rely on SNP-based methodologies featuring notable drawbacks. As a result, it is desirable to have an automated genomic cataloging system to monitor crop panels at the haplotype level. Our riceExplorer bioinformatics pipeline provides such a system that is easy to implement, cheap to use, and provides a catalog of potentially useful loci referenced against global databases. Our analyses show the potential of riceExplorer having identified 114 gene regions where potentially useful genomic haplotypes associated with a key agronomic trait (grain length) are found in NFVs, while absent among ECs.

## Data Availability Statement

The original contributions presented in the study are included in the article/Supplementary Materials, further inquiries can be directed to the corresponding author.

## Author Contributions

CD, TT, and SW: conceptualization. CD: carried out programming, workflow design, and writing the original draft. SW, VR, MS, and WA: molecular biology and sequencing. BT: genomic data curation. CD and SW: writing the review and editing. TT: supervision. All authors contributed to the article and approved the submitted version.

## Funding

This study was supported by grant from National Science and Technology Development Agency, Thailand (NSTDA grant number: P-18–51456).

## Conflict of Interest

The authors declare that the research was conducted in the absence of any commercial or financial relationships that could be construed as a potential conflict of interest.

## Publisher’s Note

All claims expressed in this article are solely those of the authors and do not necessarily represent those of their affiliated organizations, or those of the publisher, the editors and the reviewers. Any product that may be evaluated in this article, or claim that may be made by its manufacturer, is not guaranteed or endorsed by the publisher.

## Supplementary Material

Supplementary Table S4 GO Slim information for annotated genes identified across NFV accessions (generated at: http://rice.uga.edu/downloads_gad.shtml; accessed 16/09/21). See Supplementary Table S4.

Supplementary Table S5 Full table of large grain size-associated haplotypes (LGHs) found among Thai NFV accessions but not ECs across all 12 rice genome chromosomes (*cf*. Table 2 in main text). See Supplementary Table S5.
