# Supplementary material for: riceExplorer: Uncovering the Hidden Potential of a National Genomic Resource Against a Global Database
Source: Front Plant Sci. 2022 Apr 29;13:781153. doi: 10.3389/fpls.2022.781153 (PMC9100803; doi:10.3389/fpls.2022.781153)
Supplement: Supplementary Table S4 — GO Slim information for annotated genes identified across NFV accessions (generated at: http://rice.uga.edu/downloads_gad.shtml; accessed 16/09/21). See Supplementary Table S4. [file Data_Sheet_1.zip › SI2/SupplInfo_2022_03_25.docx]

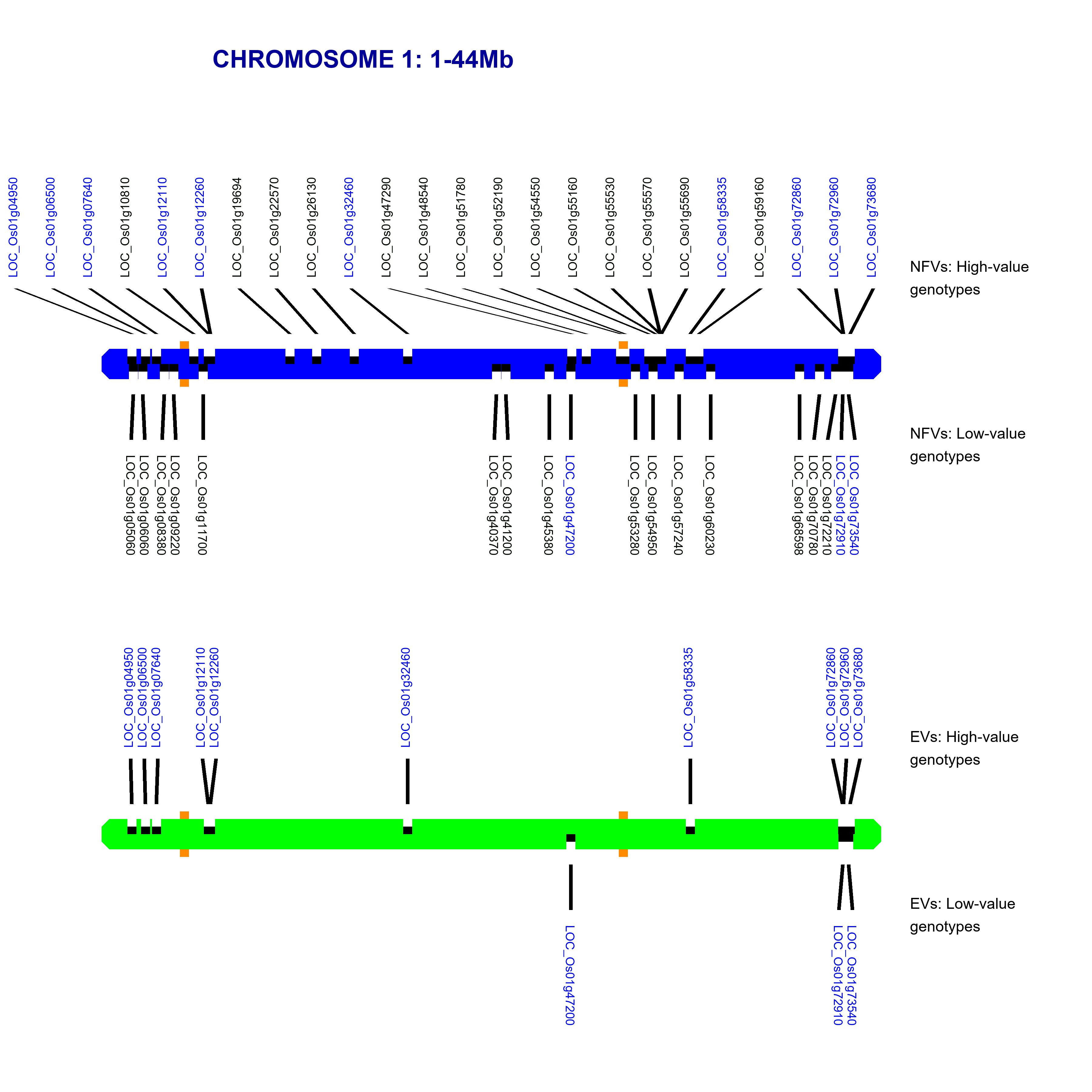
 Figure S1a. Potential LGHs on chromosome 1.


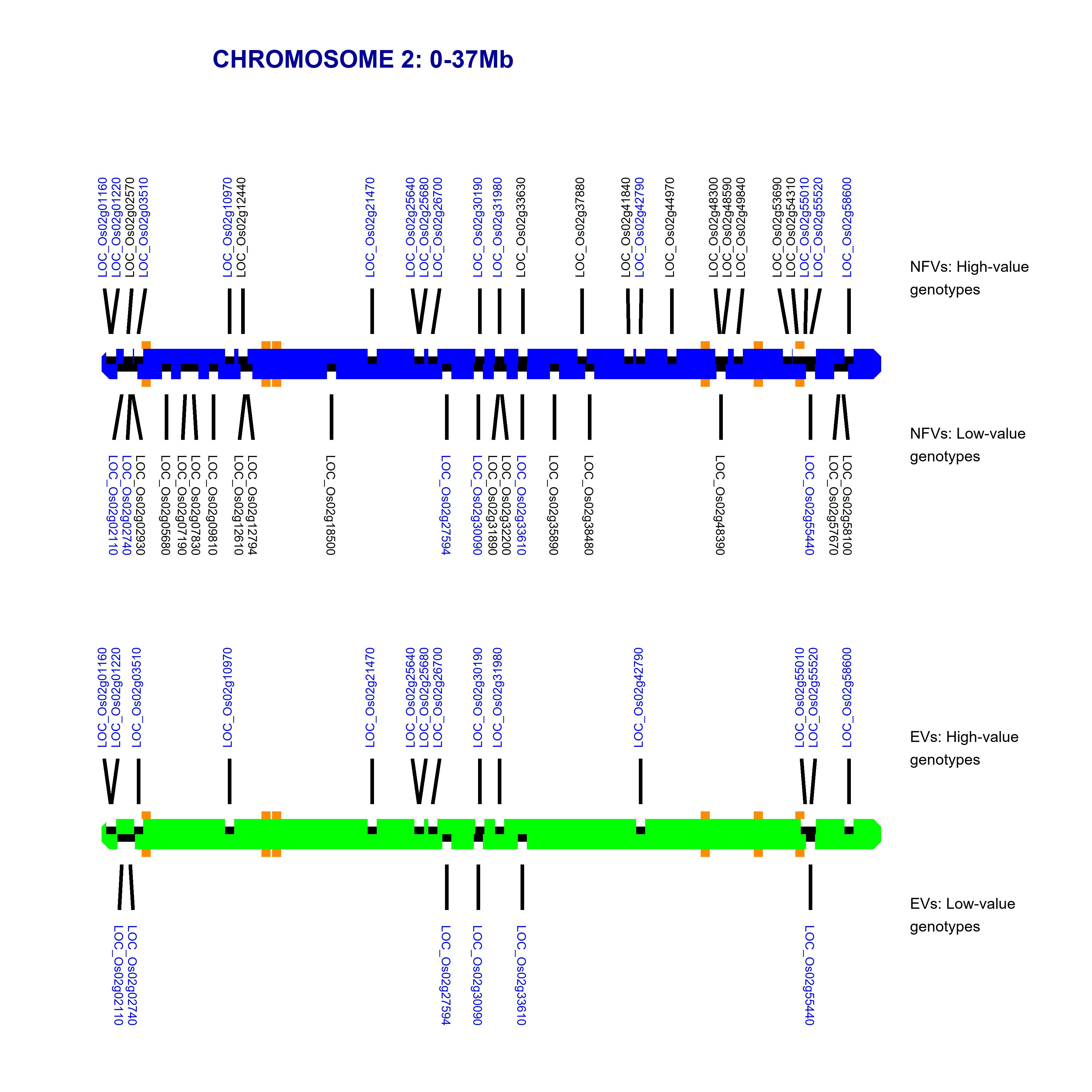
 Figure S1b. Potential LGHs on chromosome 2.


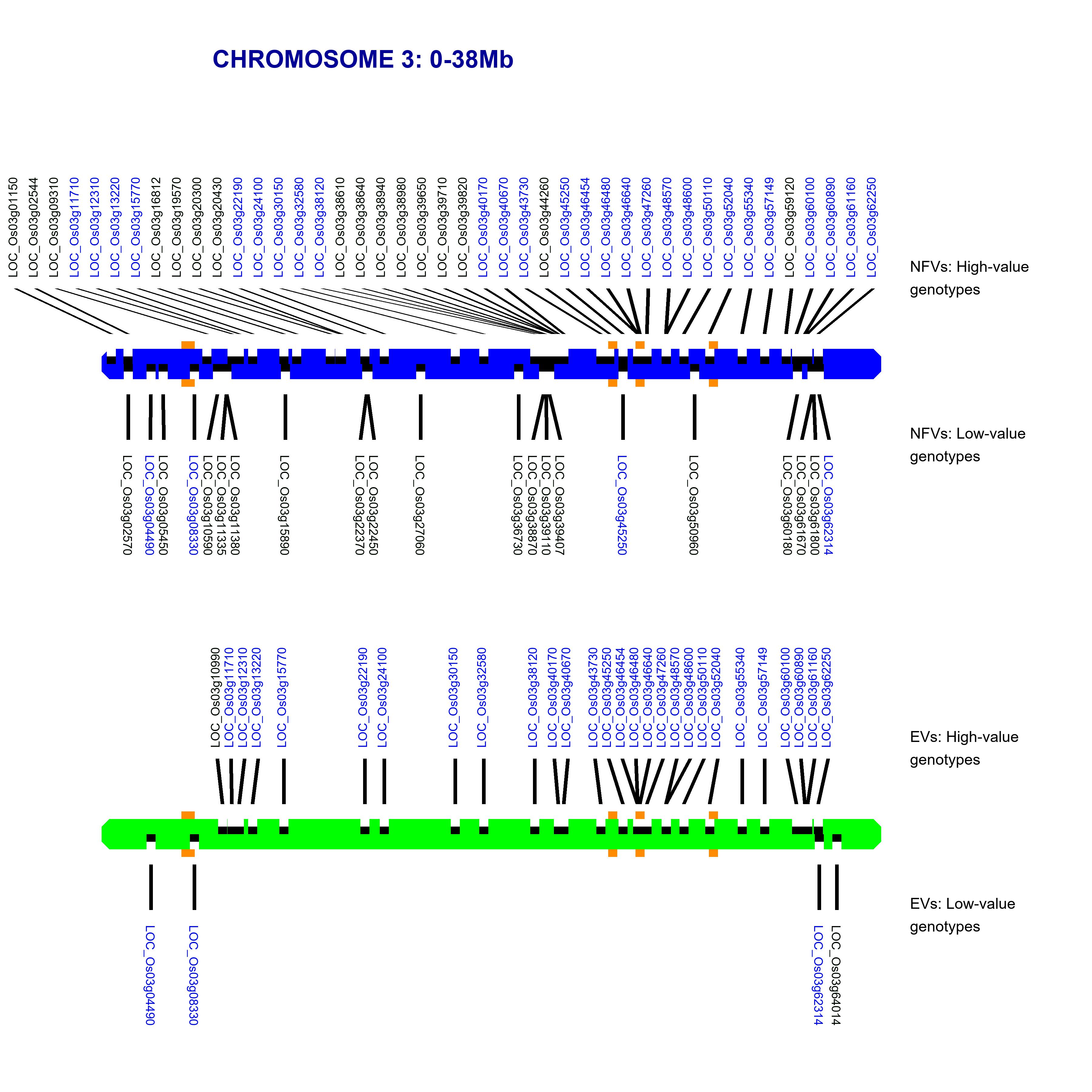
 Figure S1c. Potential LGHs on chromosome 3.


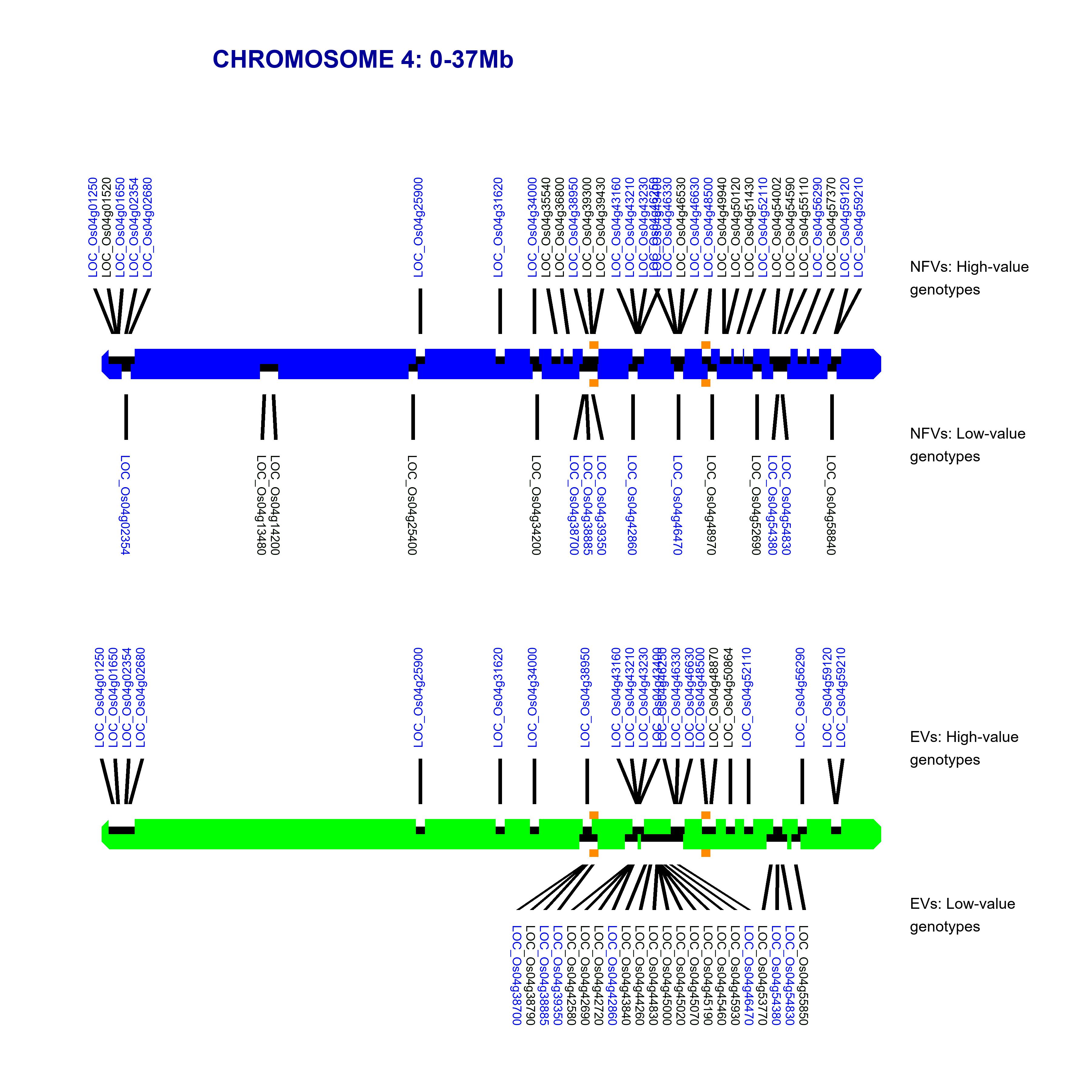
 Figure S1d. Potential LGHs on chromosome 4.


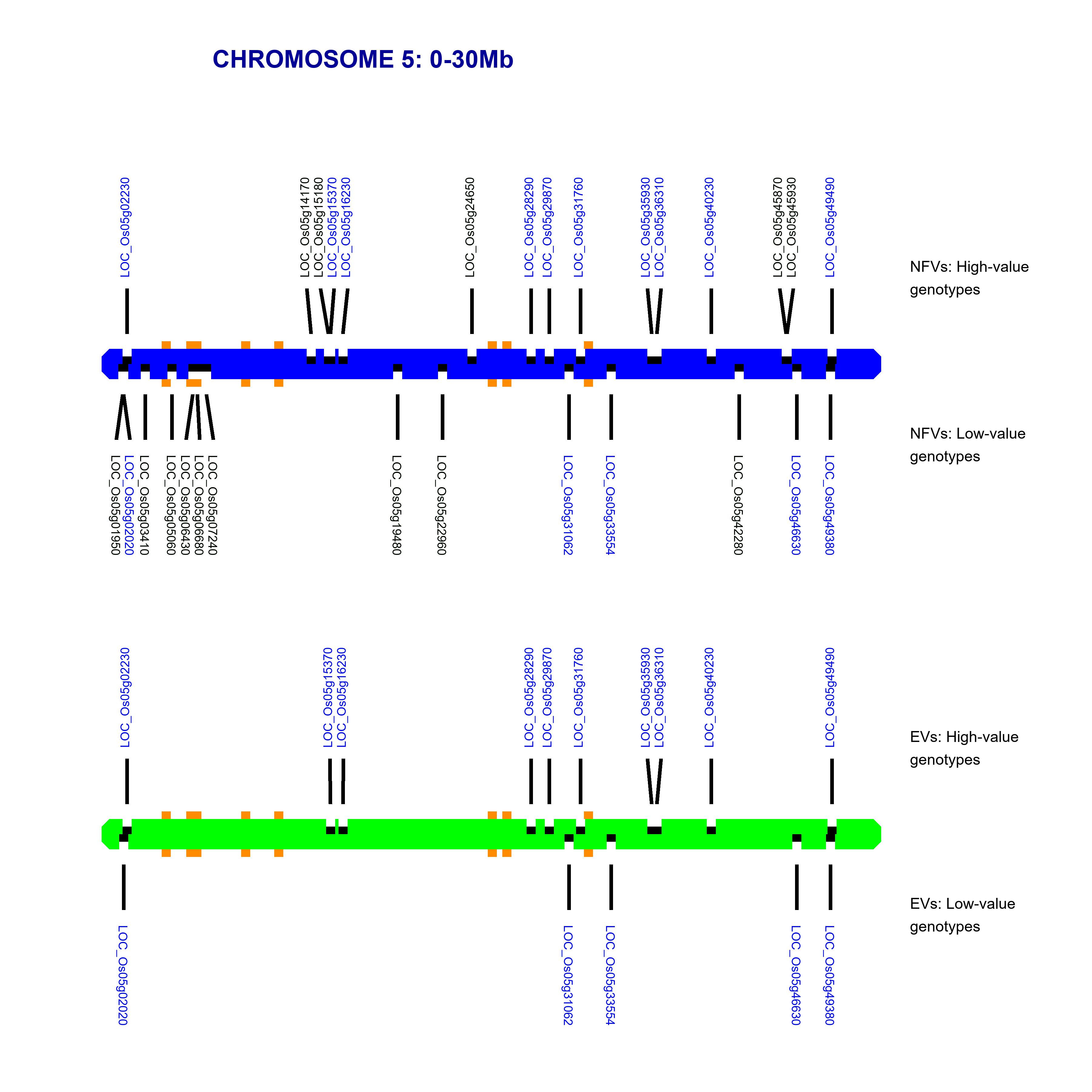
 Figure S1e. Potential LGHs on chromosome 5.


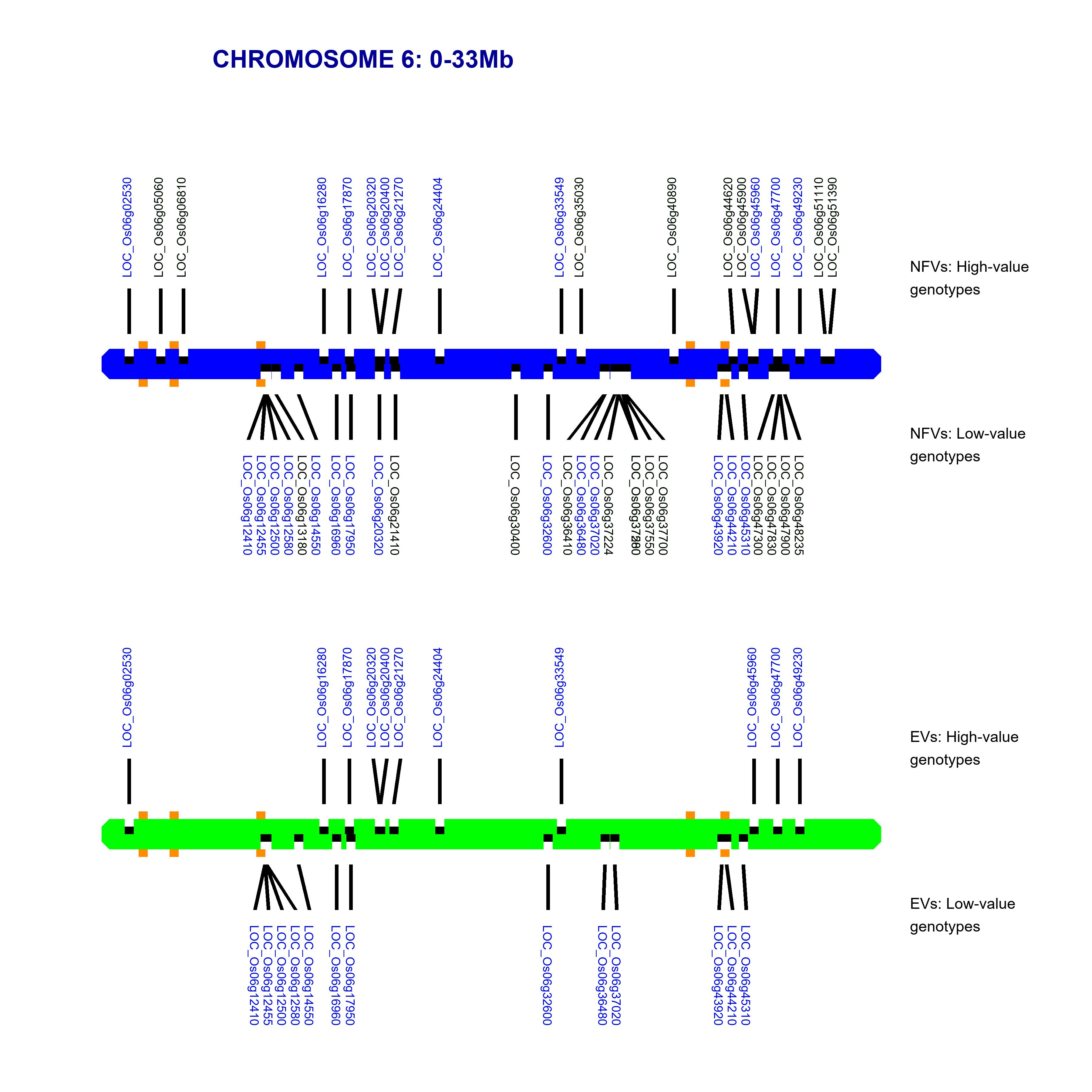
 Figure S1f. Potential LGHs on chromosome 6.


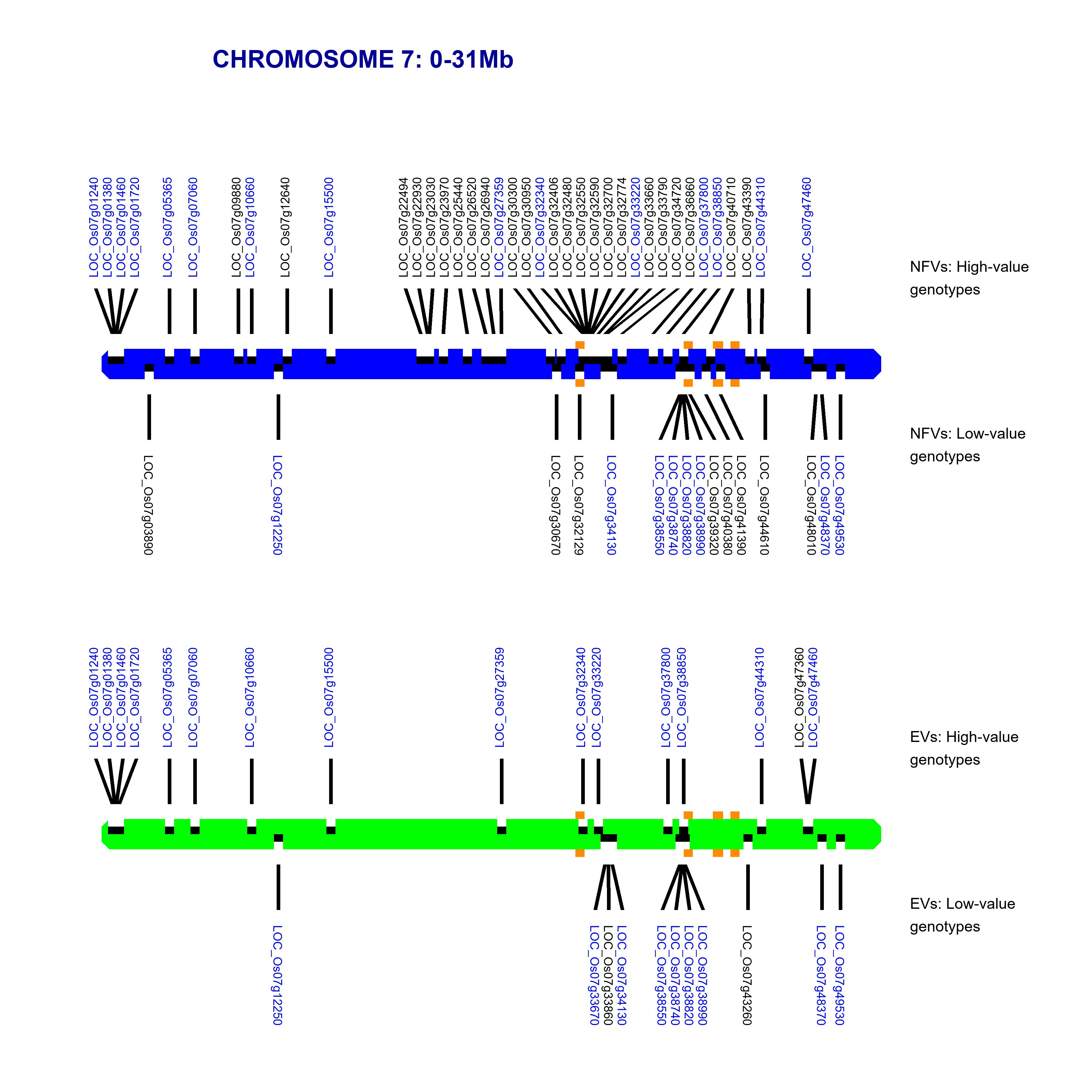
 Figure S1g. Potential LGHs on chromosome 7.


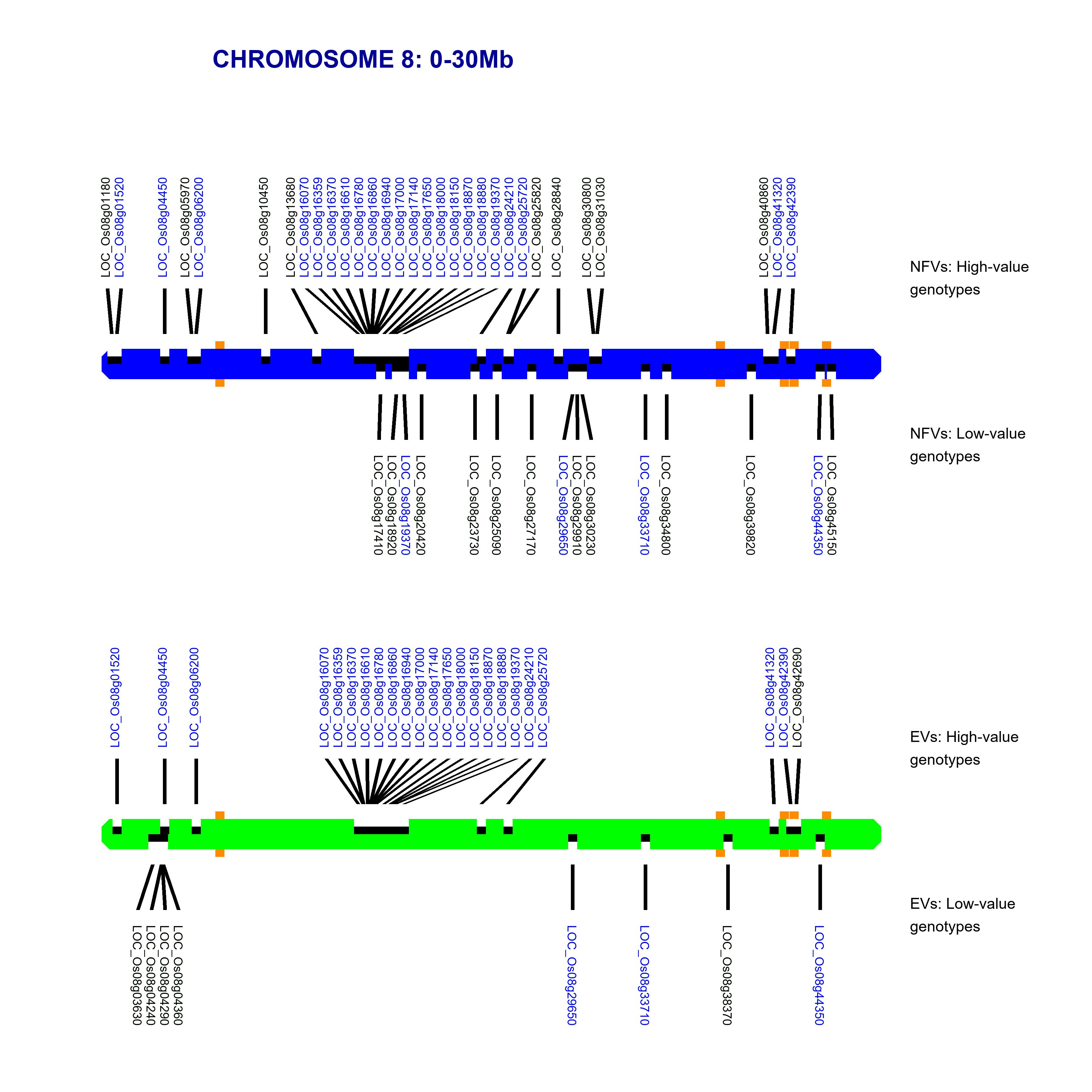
 Figure S1h. Potential LGHs on chromosome 8.


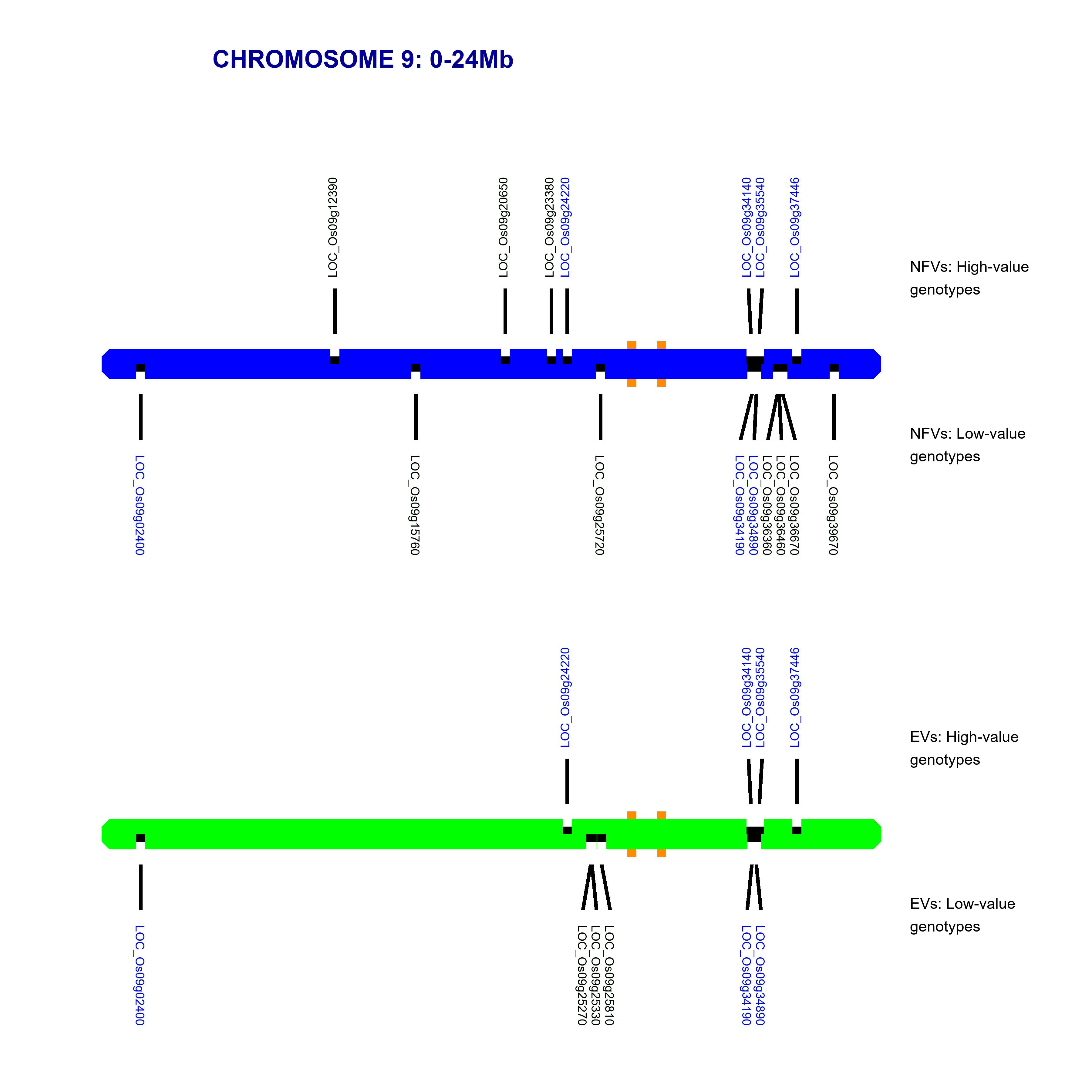
 Figure S1i. Potential LGHs on chromosome 9.


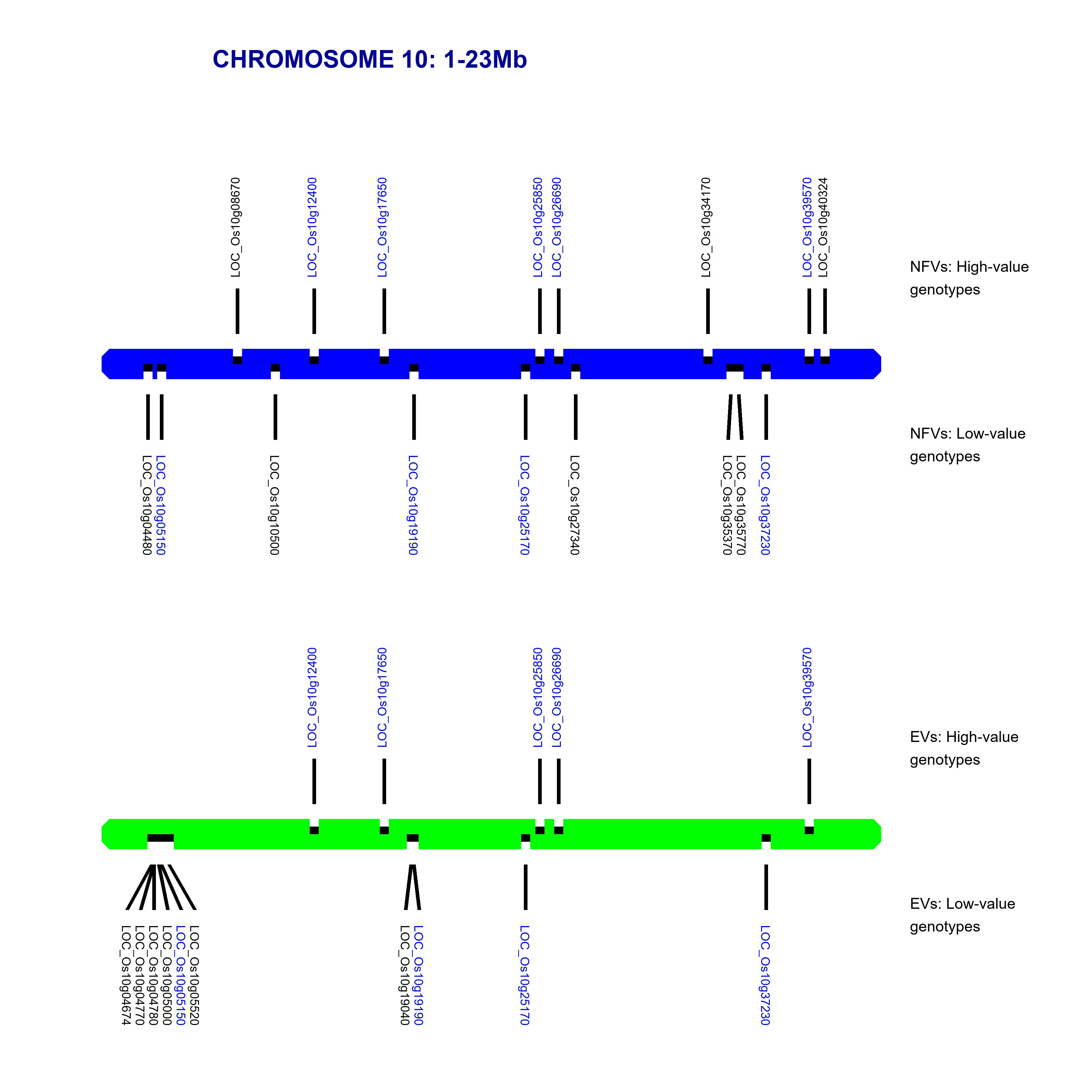
 Figure S1j. Potential LGHs on chromosome 10.


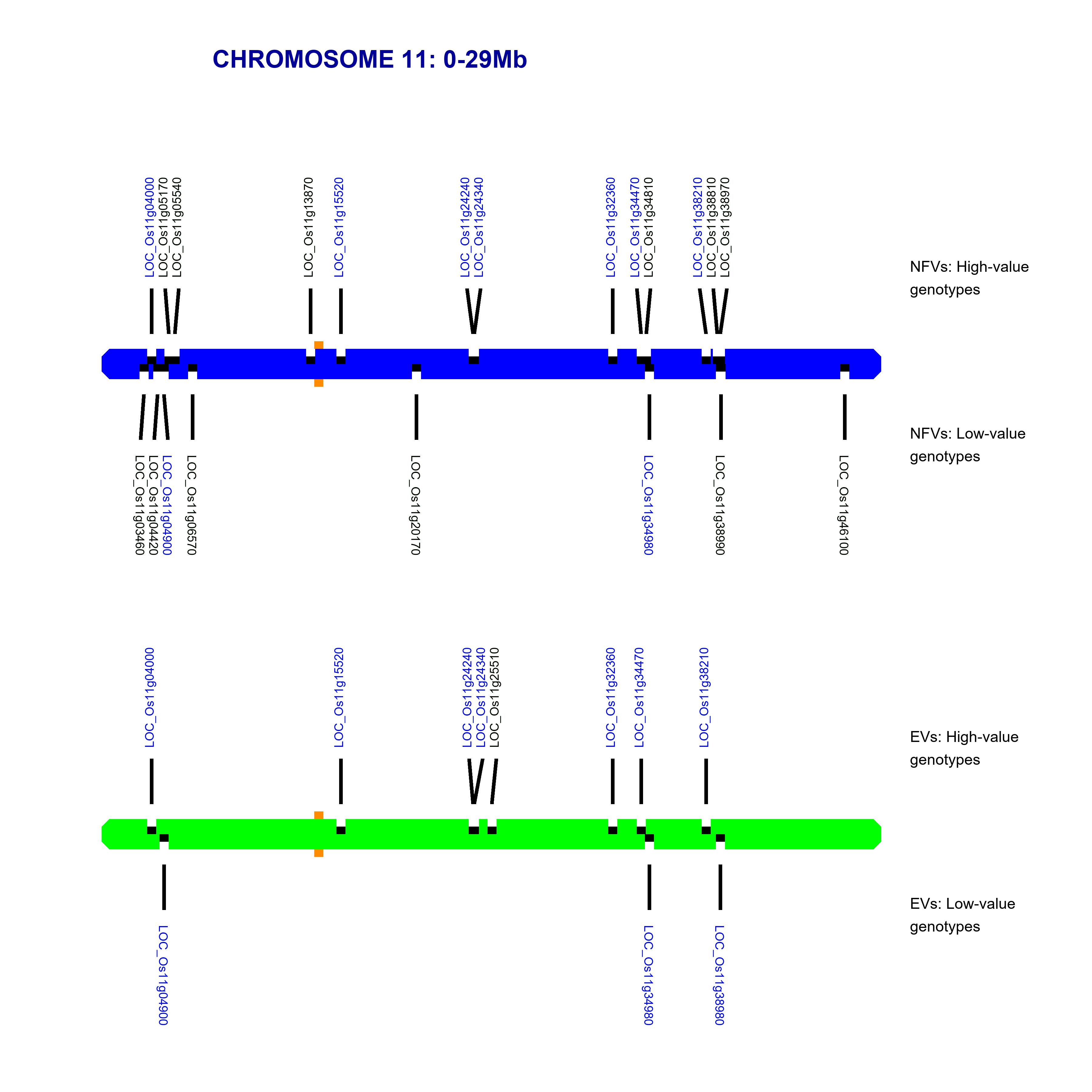
 Figure S1k. Potential LGHs on chromosome 11.


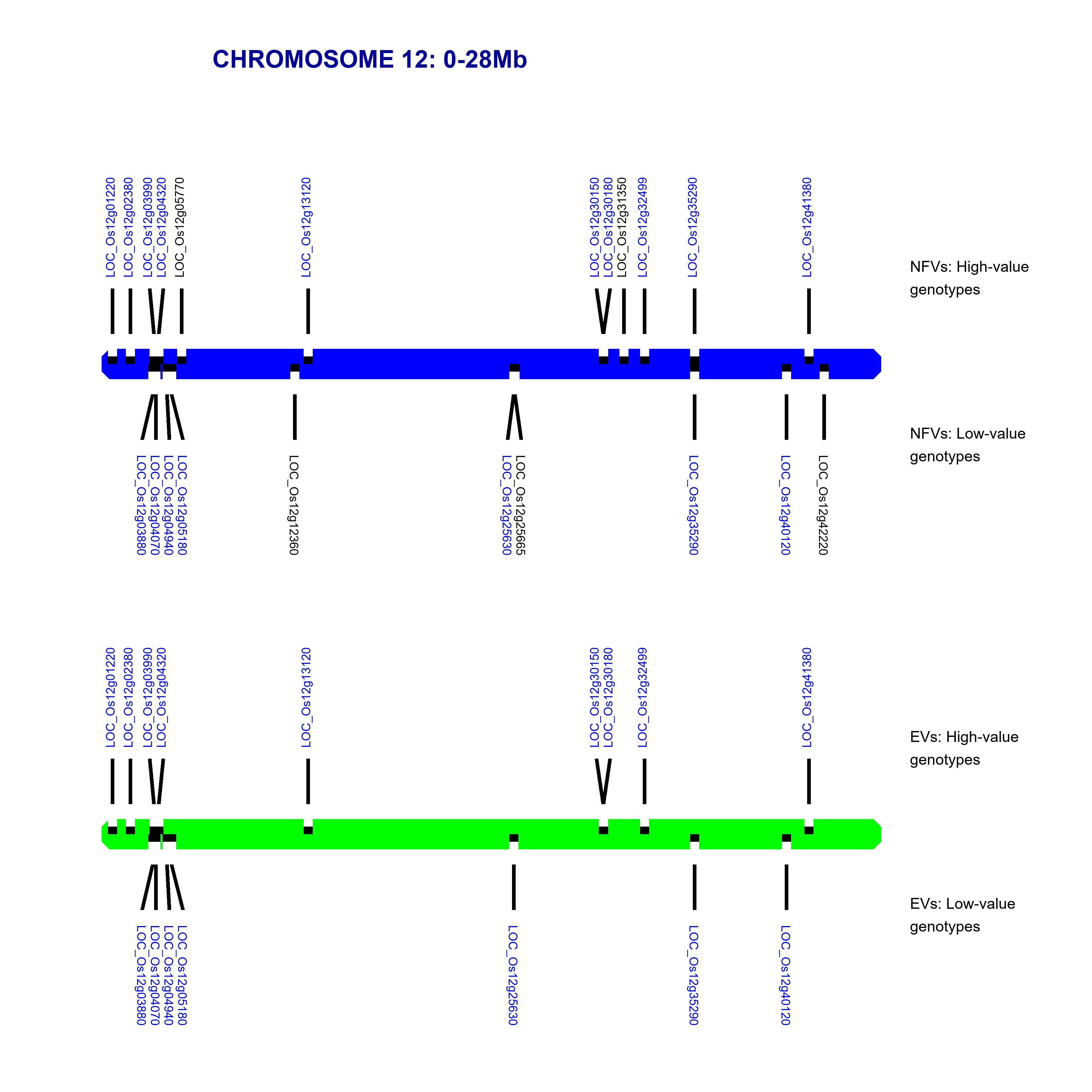
 Figure S1l. Potential LGHs on chromosome 12.


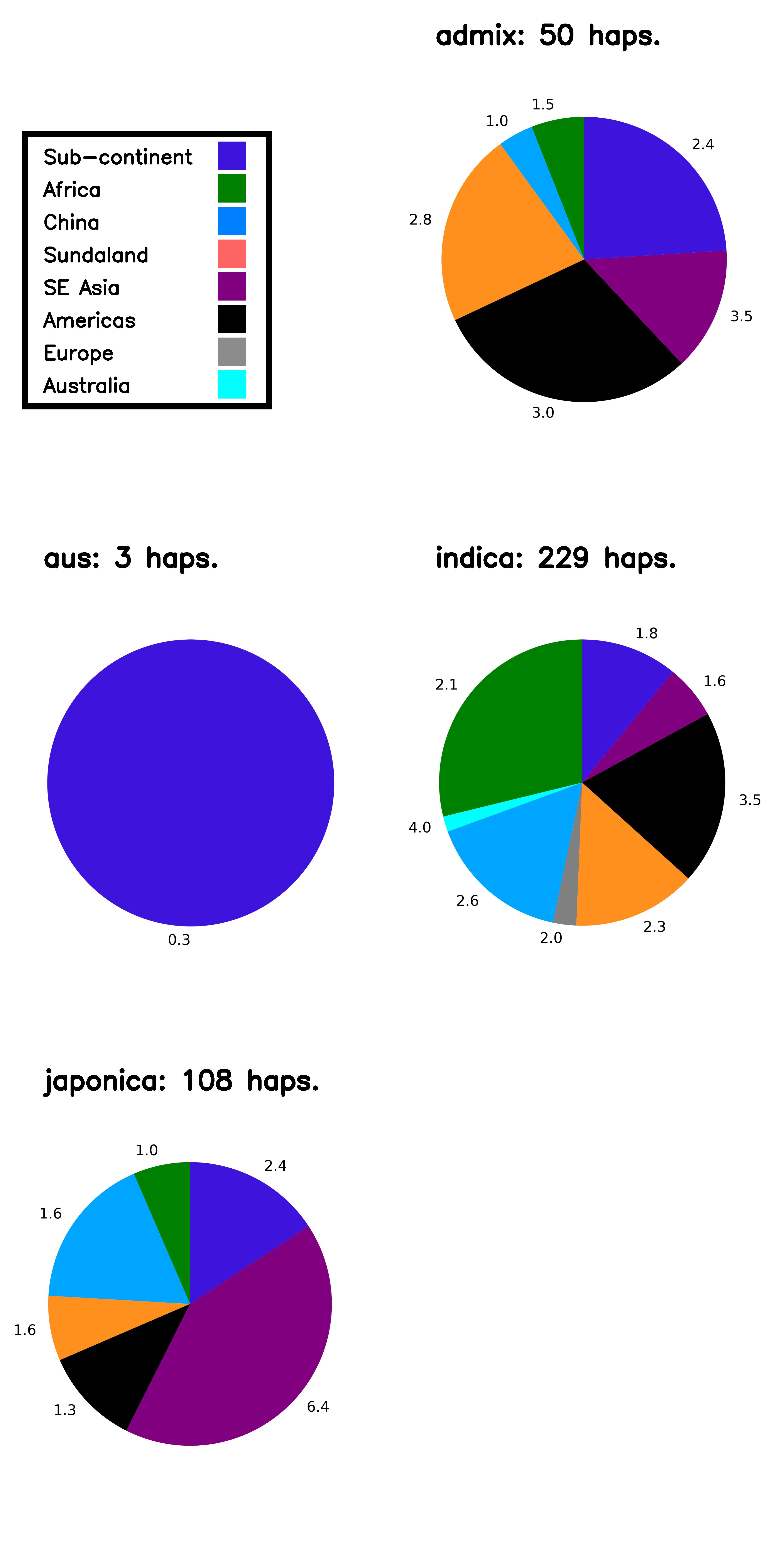


Figure S2a. IRRI database origins of high value associated haplotypes on chromosome 1.


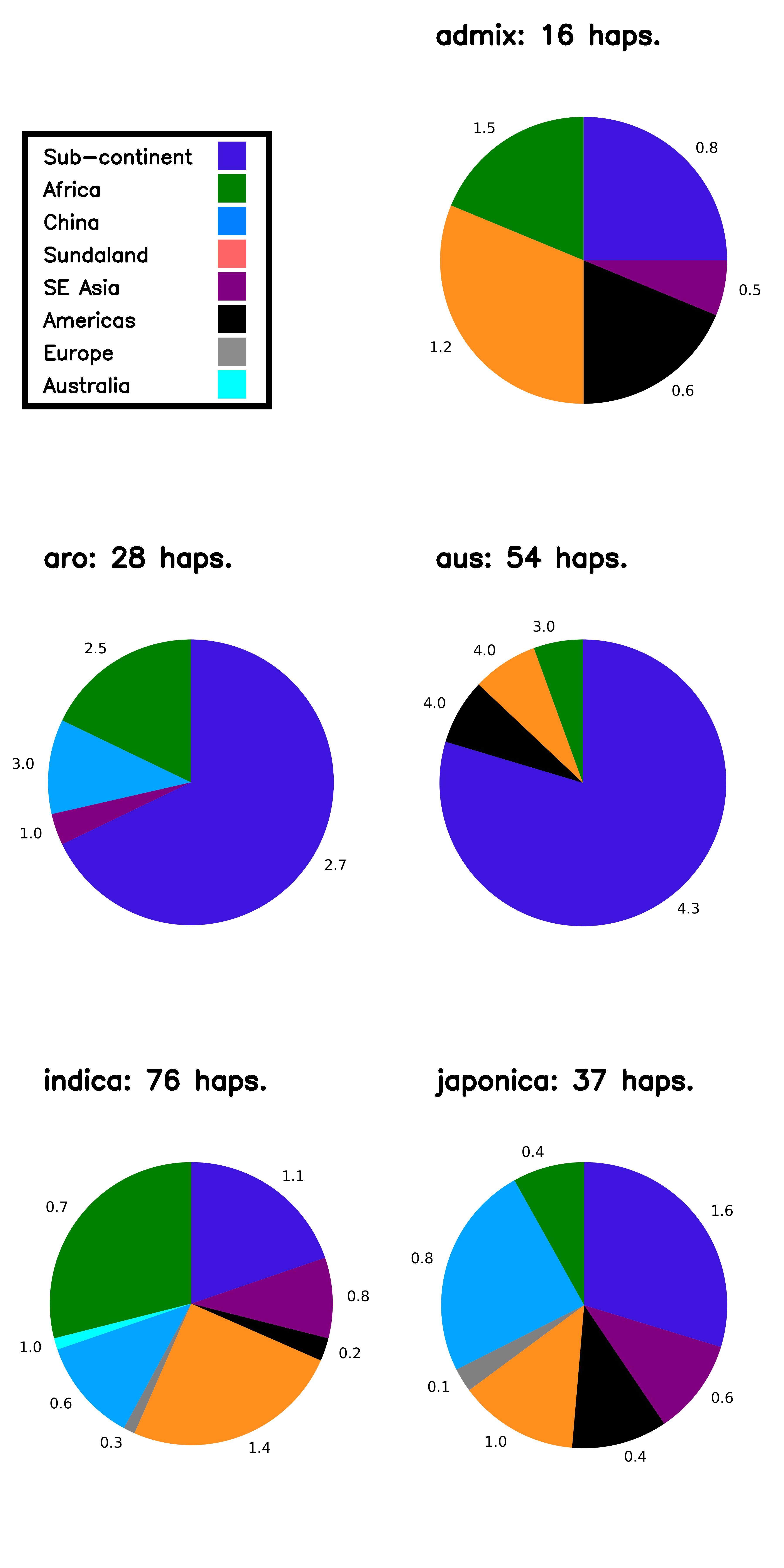


Figure S2b. IRRI database origins of low value associated haplotypes on chromosome 1.


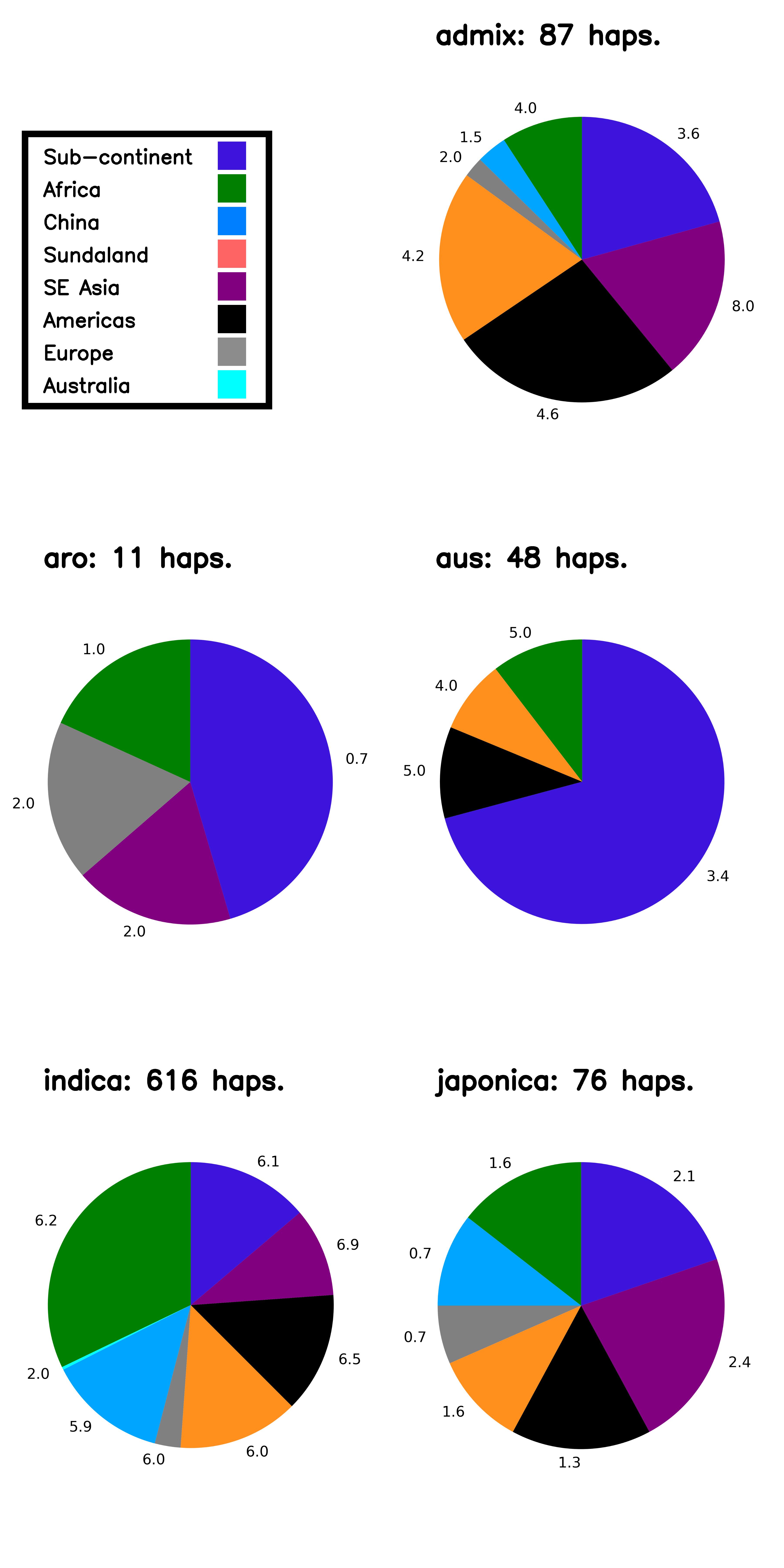


Figure S2c. IRRI database origins of high value associated haplotypes on chromosome 2.


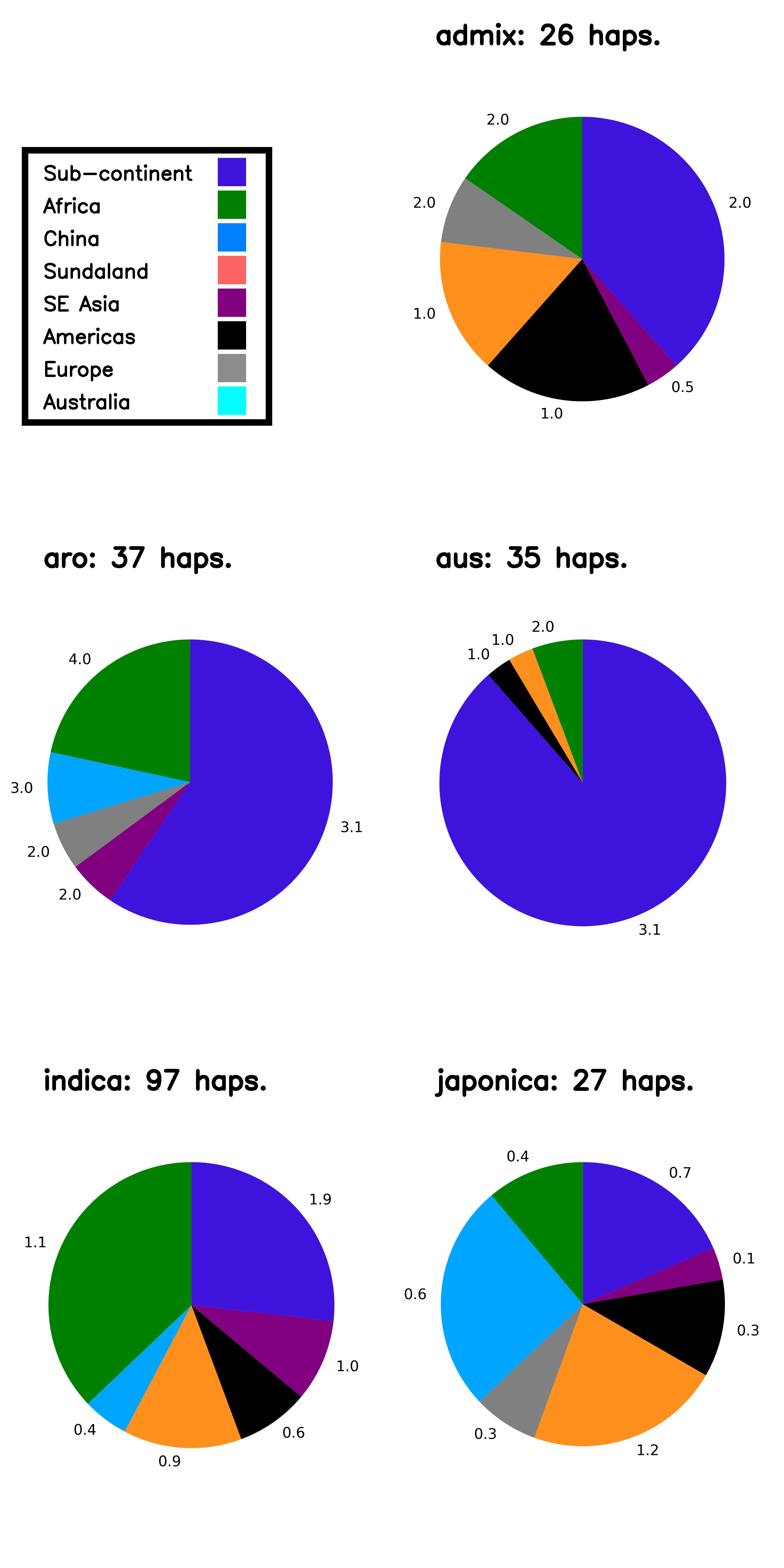


Figure S2d. IRRI database origins of low value associated haplotypes on chromosome 2.


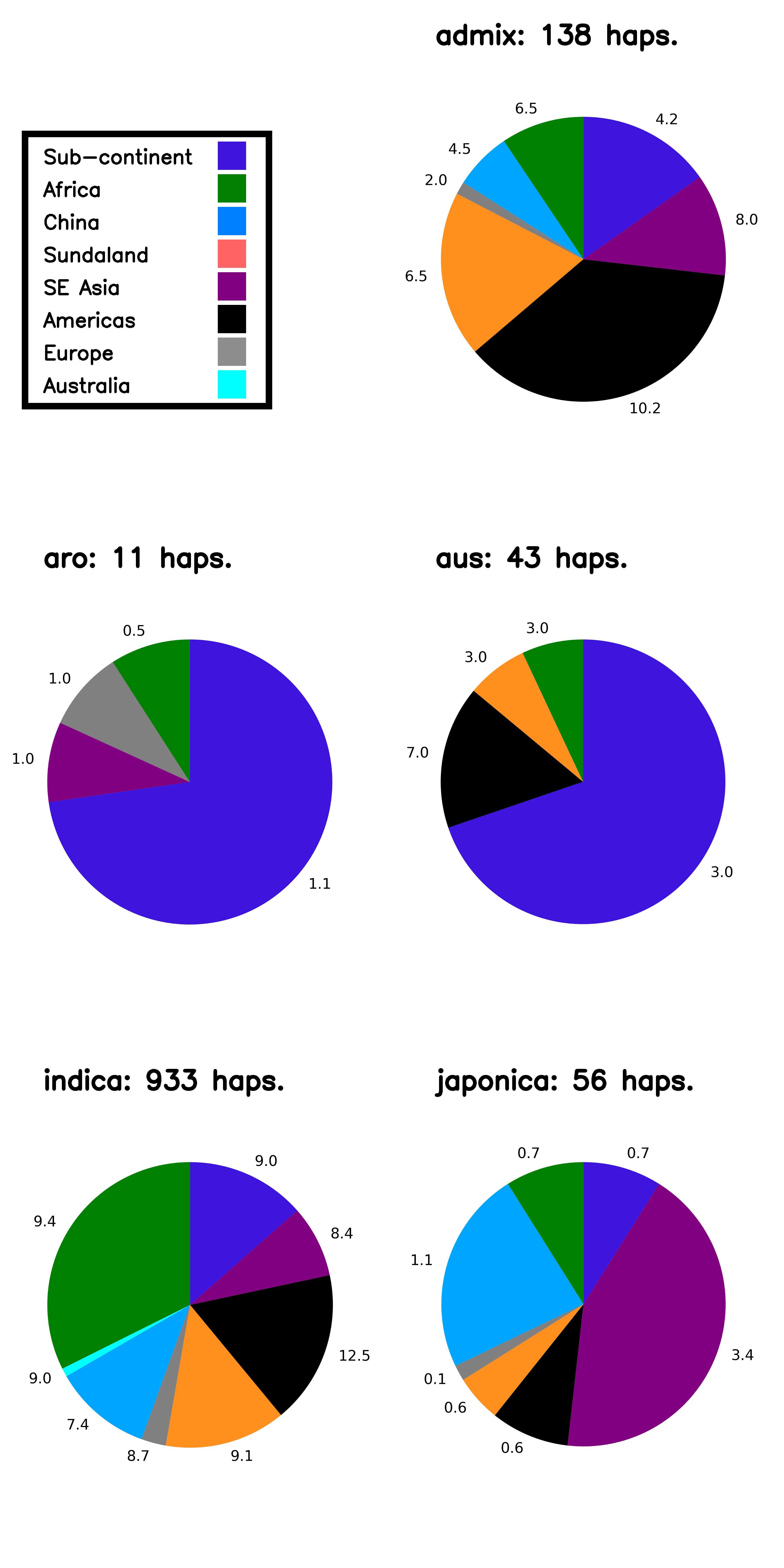


Figure S2e. IRRI database origins of high value associated haplotypes on chromosome 3.


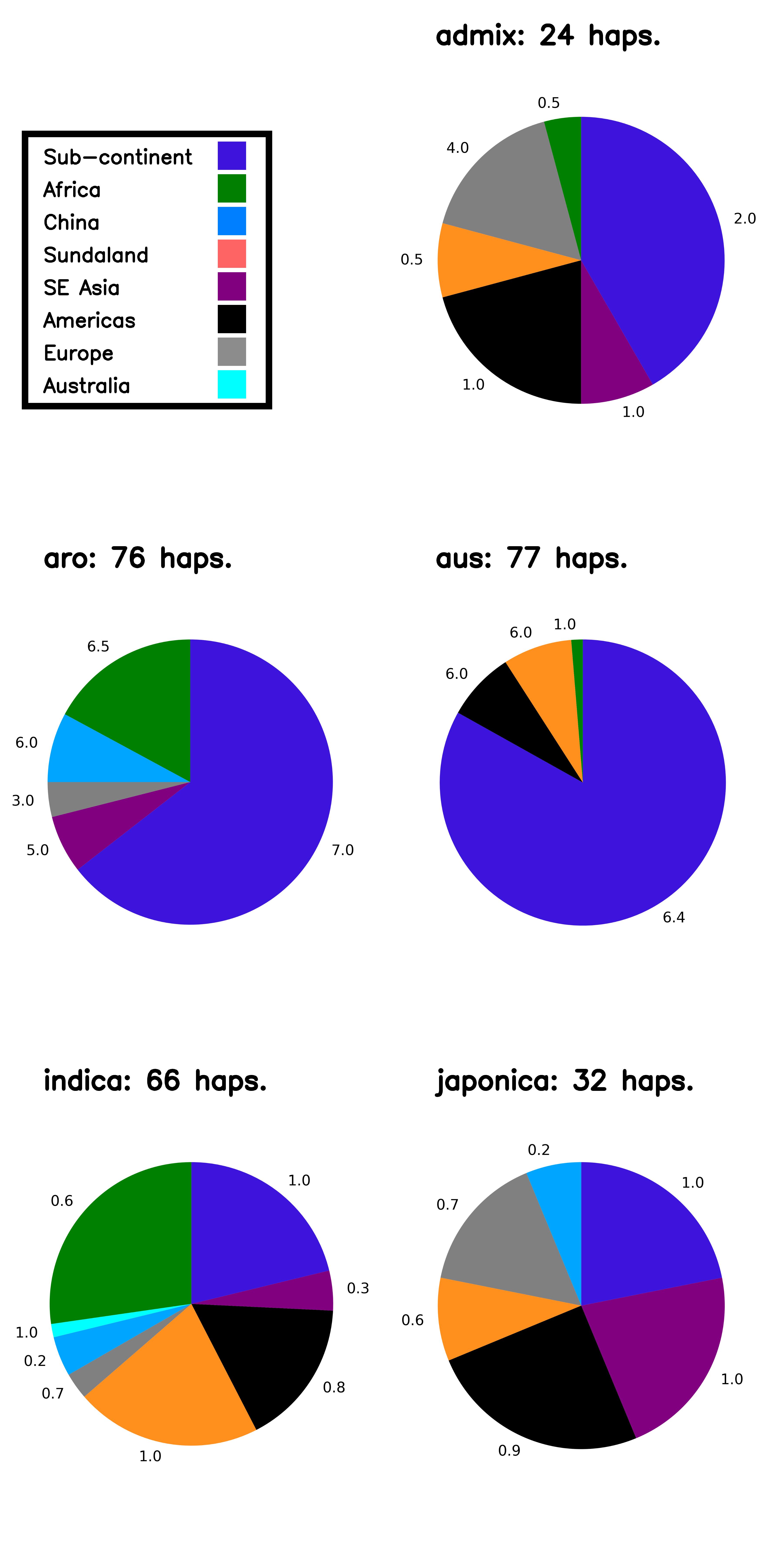


Figure S2f. IRRI database origins of low value associated haplotypes on chromosome 3.


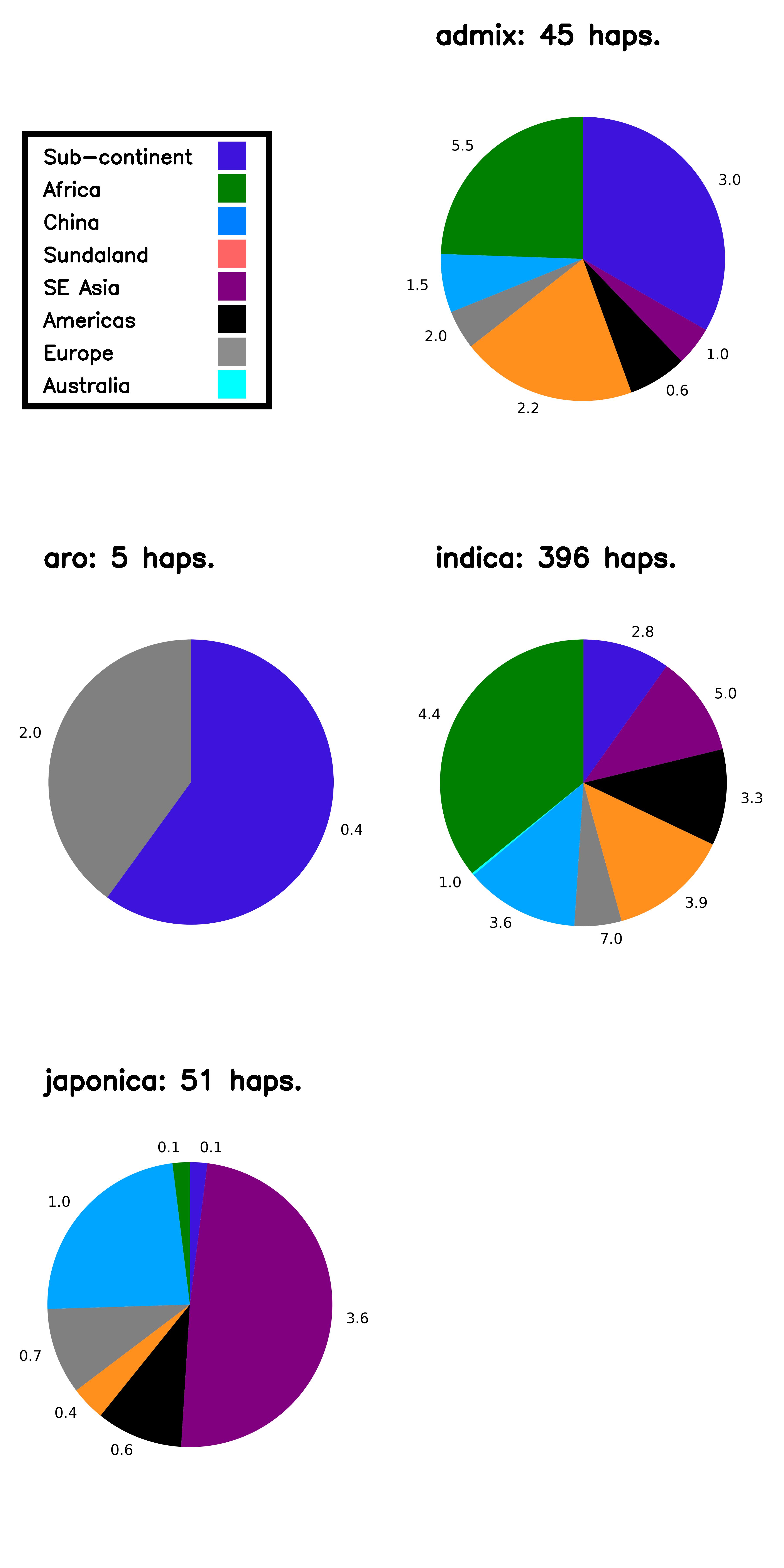


Figure S2g. IRRI database origins of high value associated haplotypes on chromosome 4.


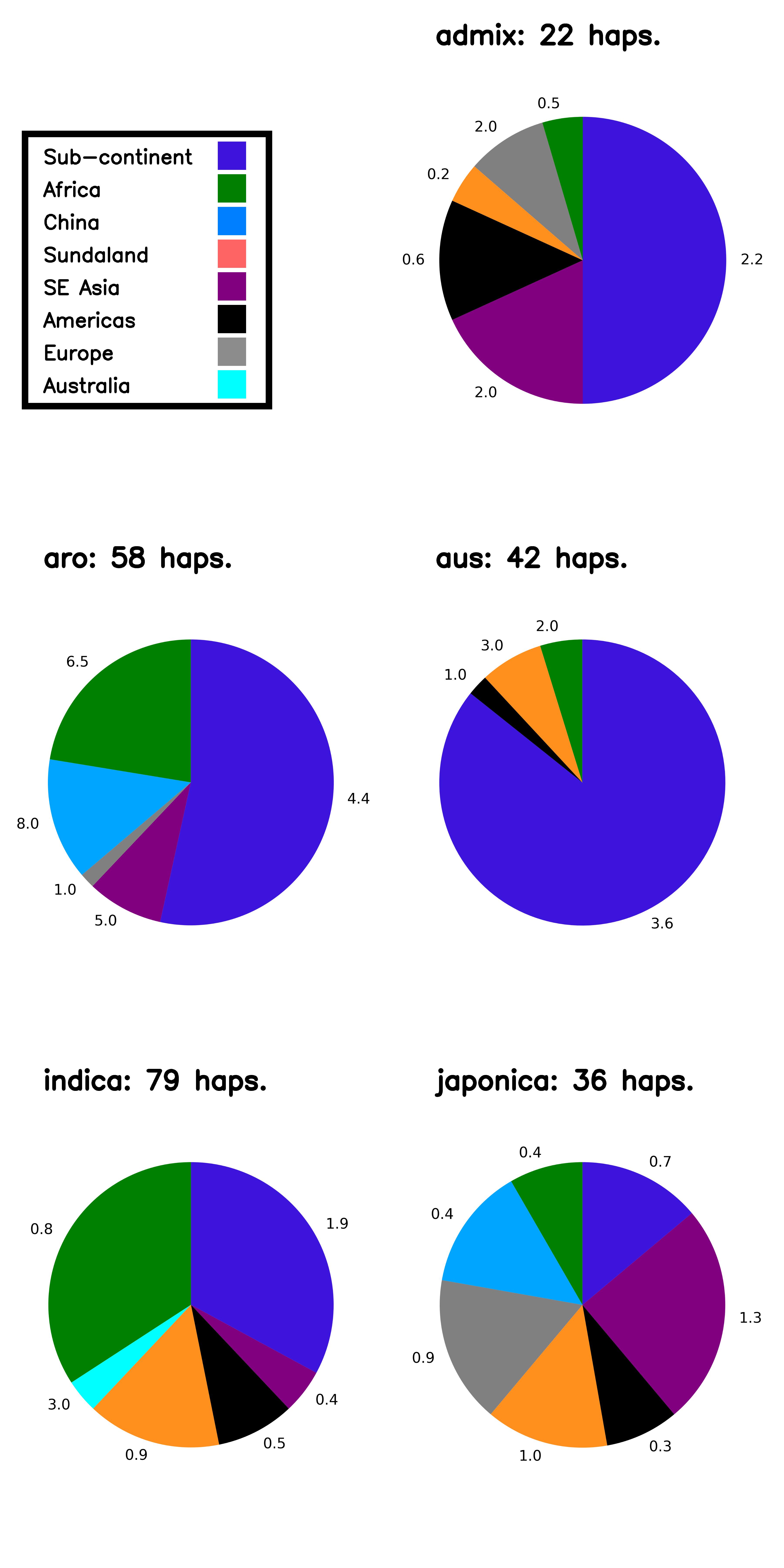


Figure S2h. IRRI database origins of low value associated haplotypes on chromosome 4.


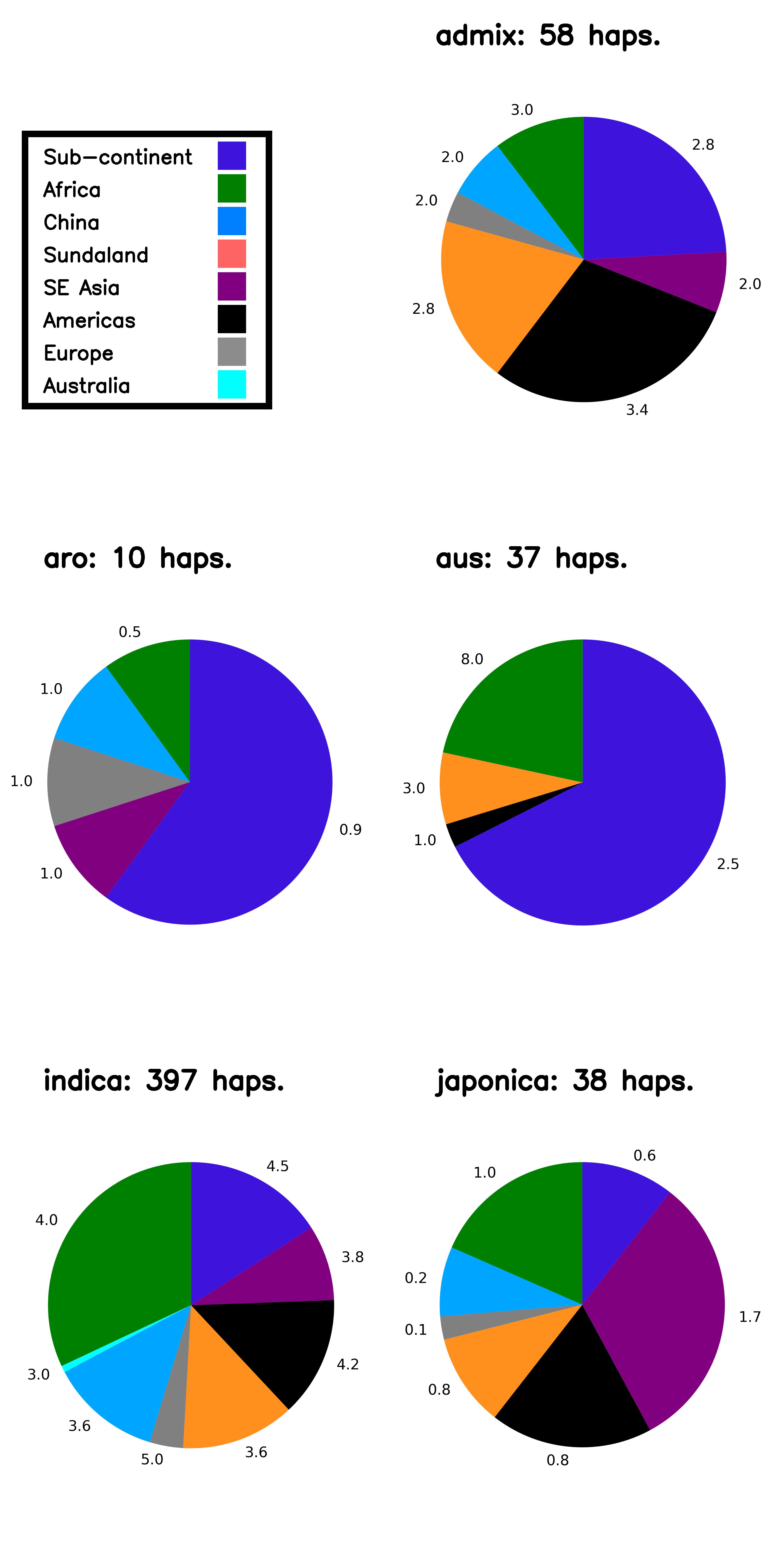


Figure S2i. IRRI database origins of high value associated haplotypes on chromosome 5.


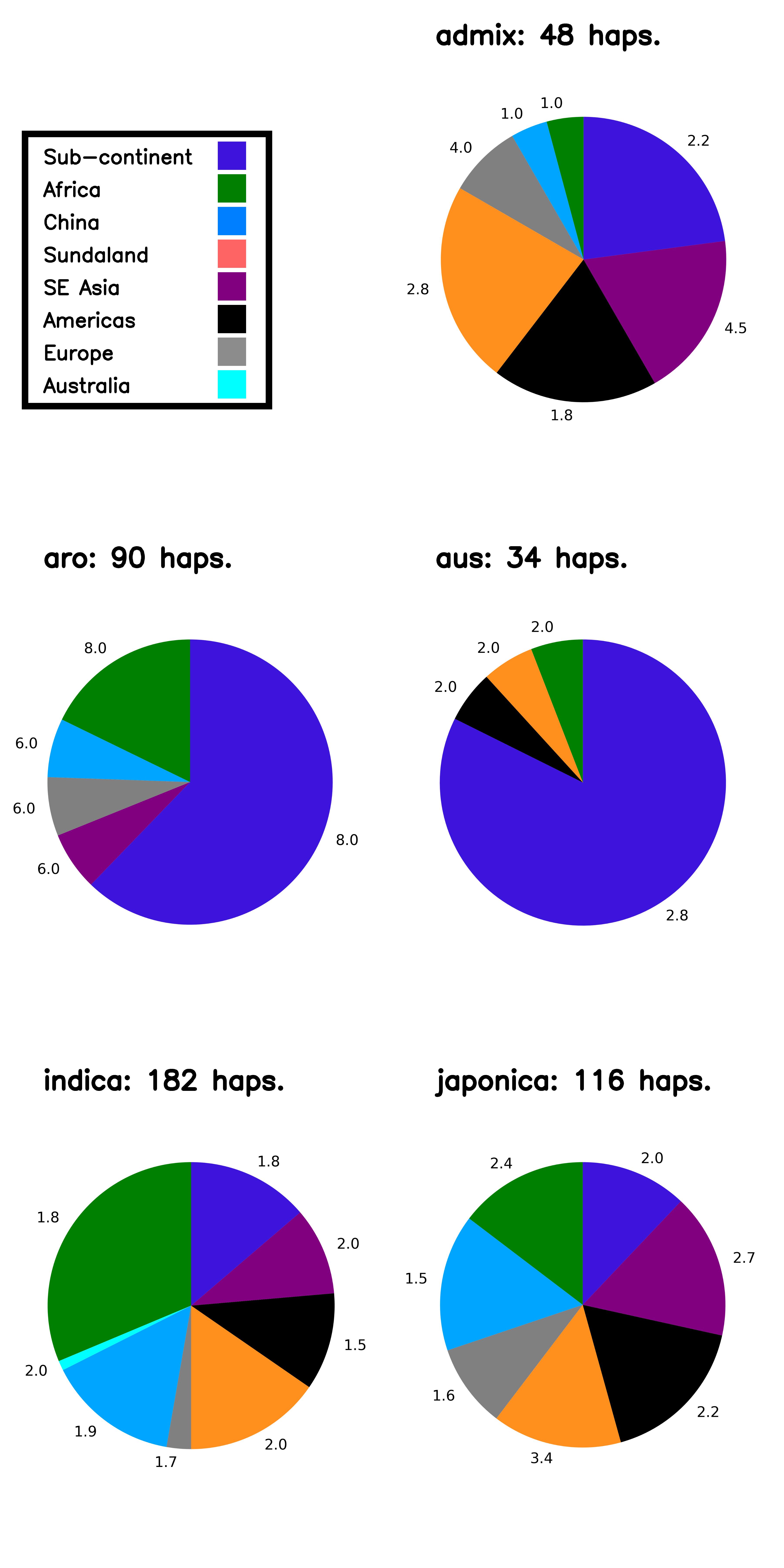


Figure S2j. IRRI database origins of low value associated haplotypes on chromosome 5.


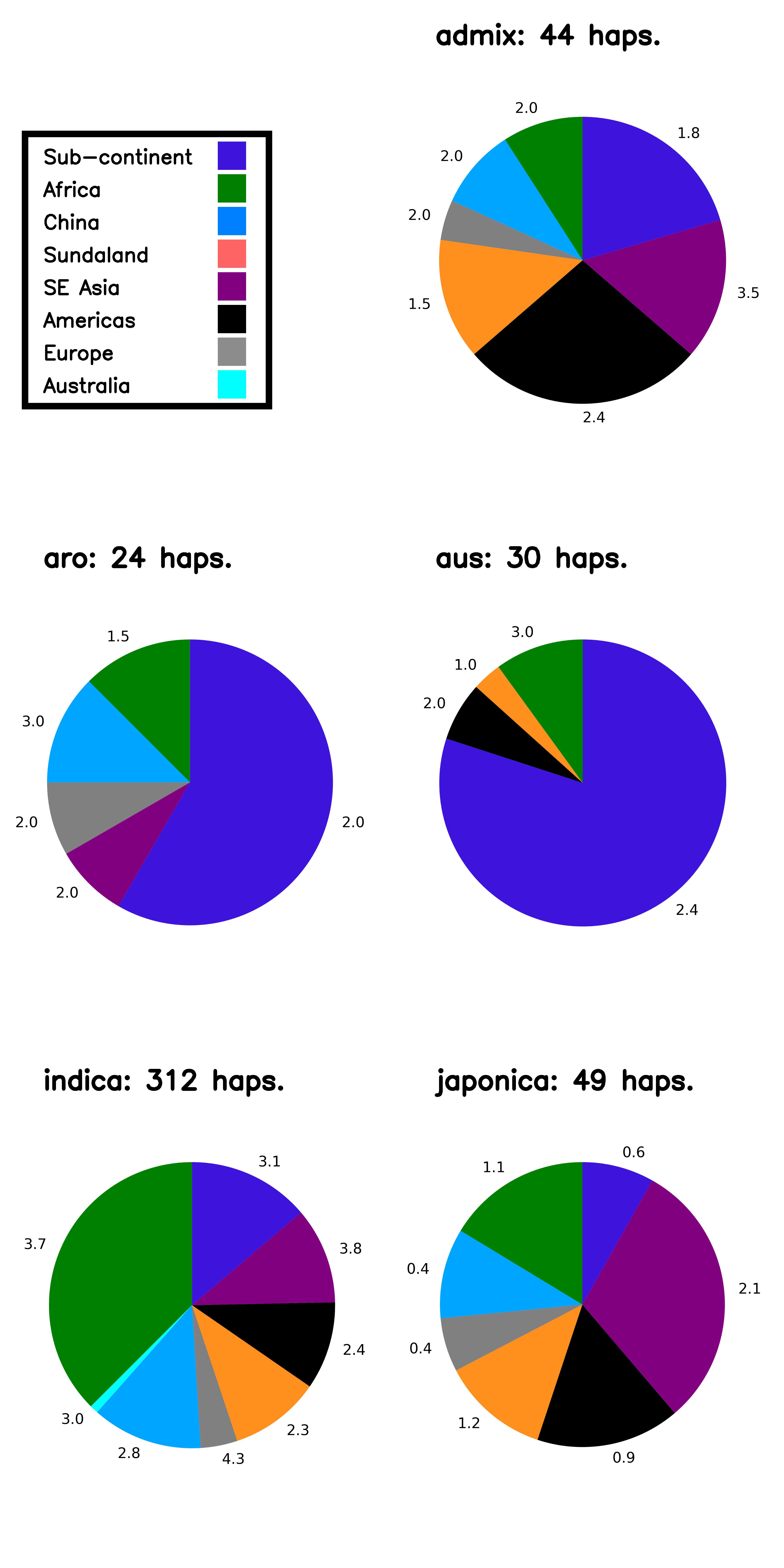


Figure S2k. IRRI database origins of high value associated haplotypes on chromosome 6.


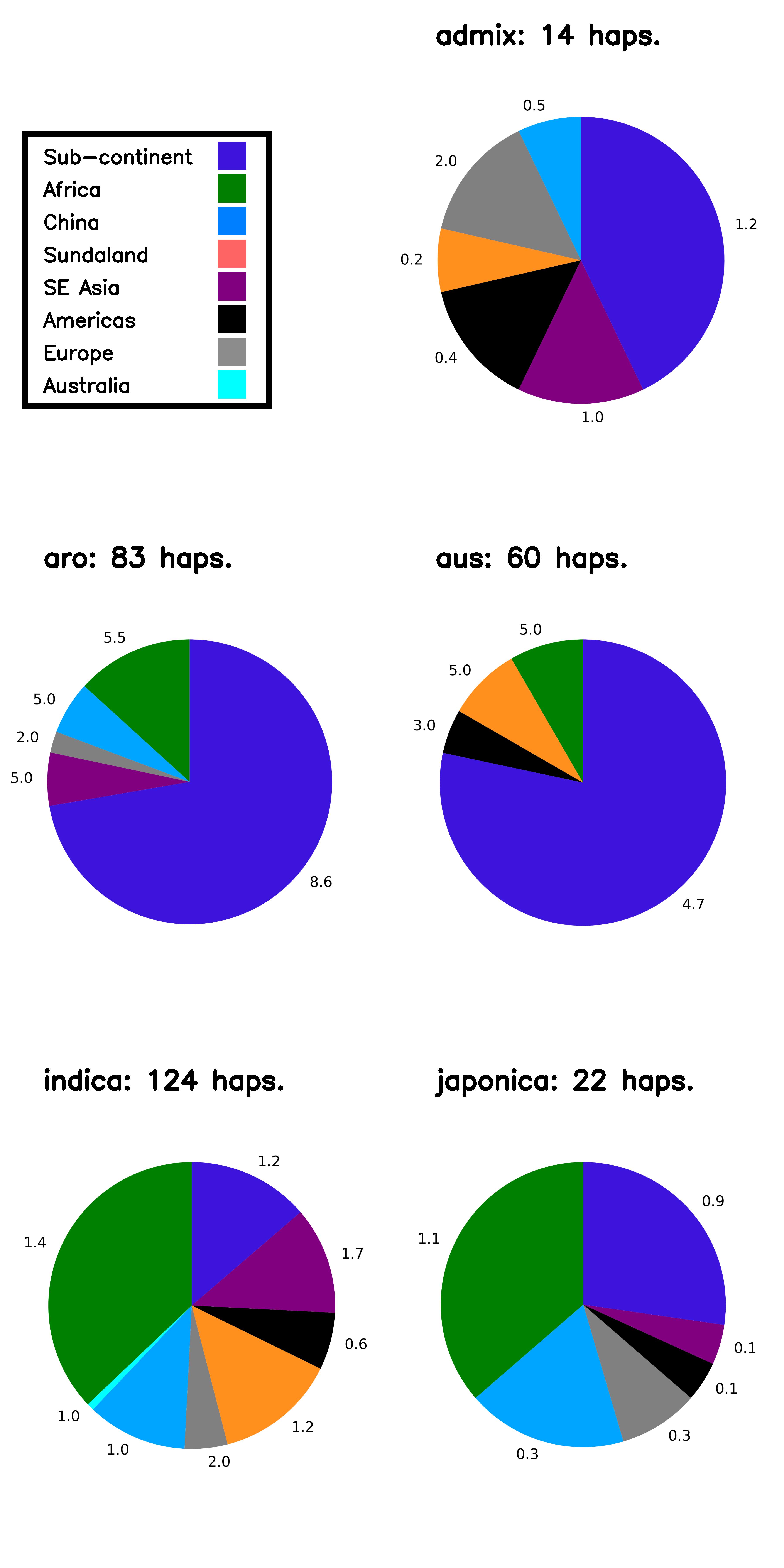


Figure S2l. IRRI database origins of low value associated haplotypes on chromosome 6.


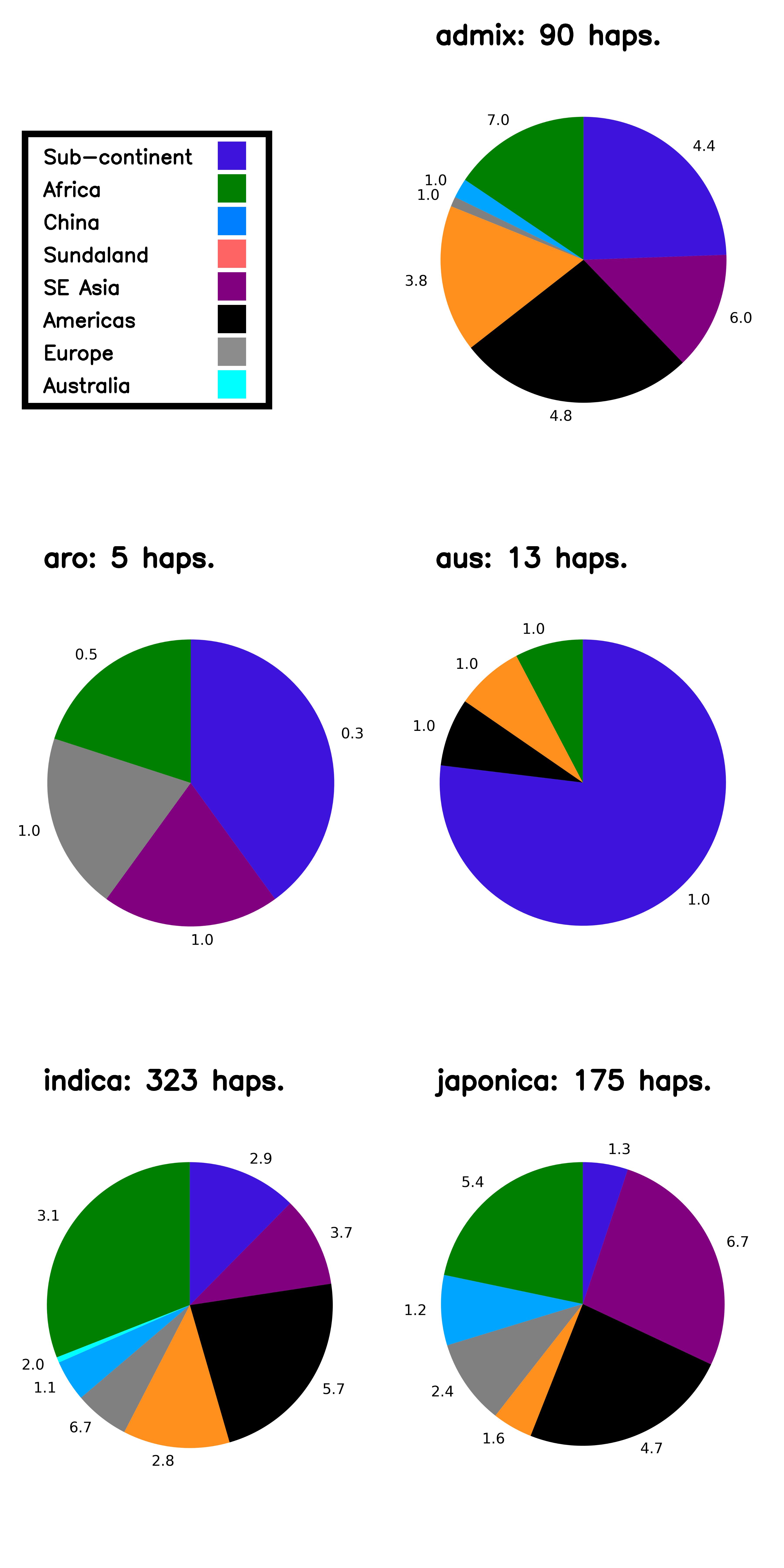


Figure S2m. IRRI database origins of high value associated haplotypes on chromosome 7.


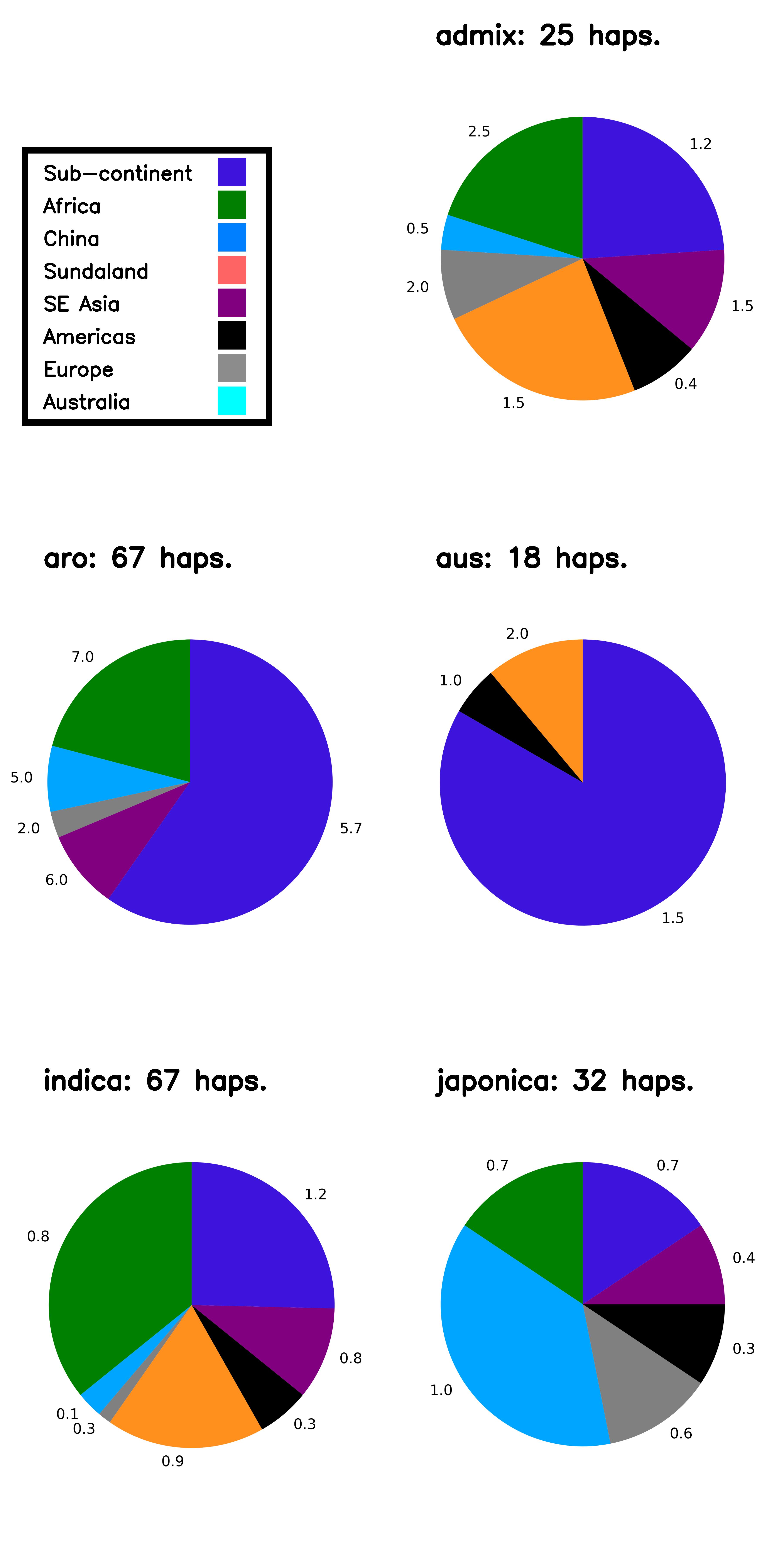


Figure S2n. IRRI database origins of low value associated haplotypes on chromosome 7.


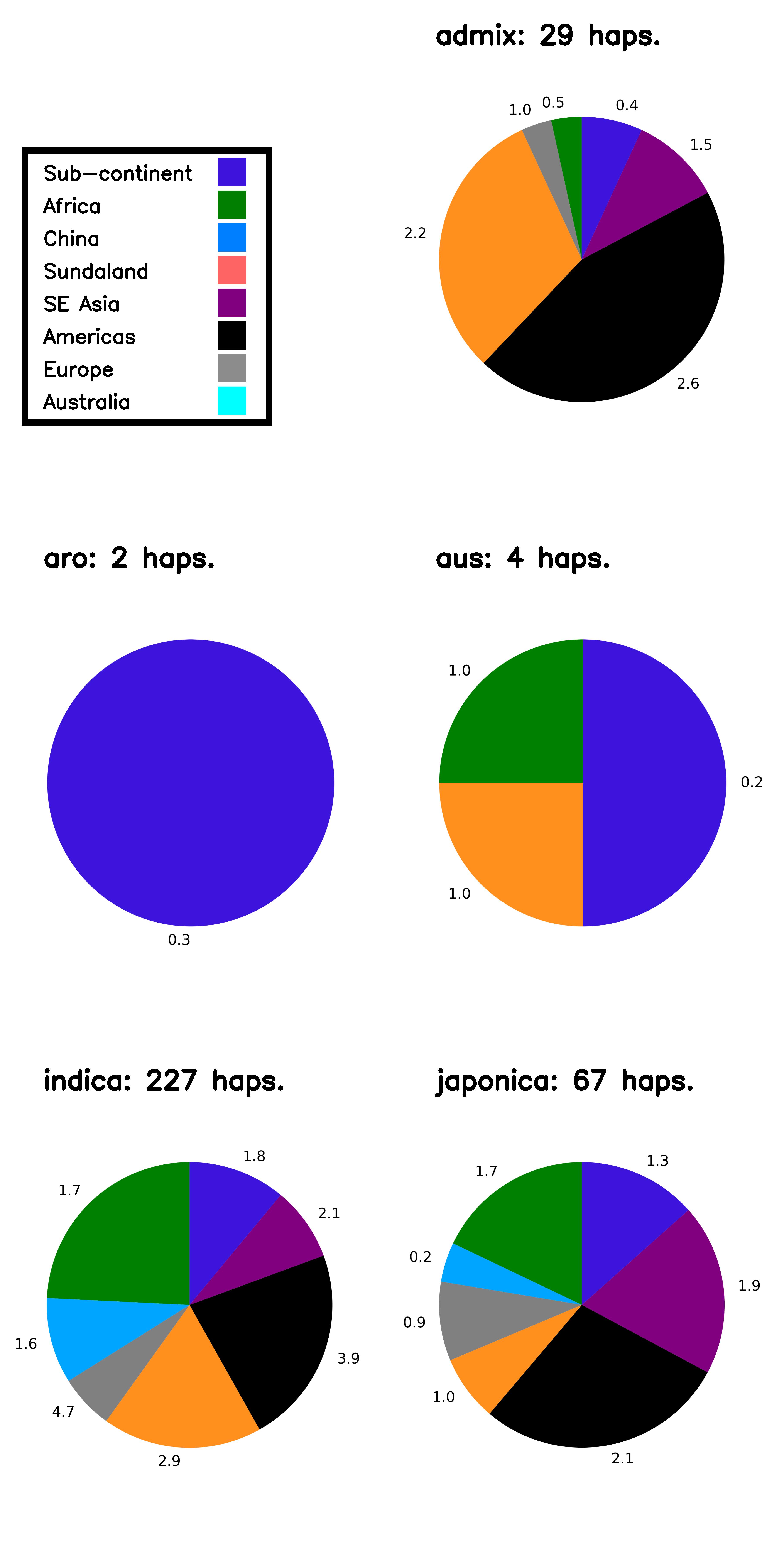


Figure S2o. IRRI database origins of high value associated haplotypes on chromosome 8.


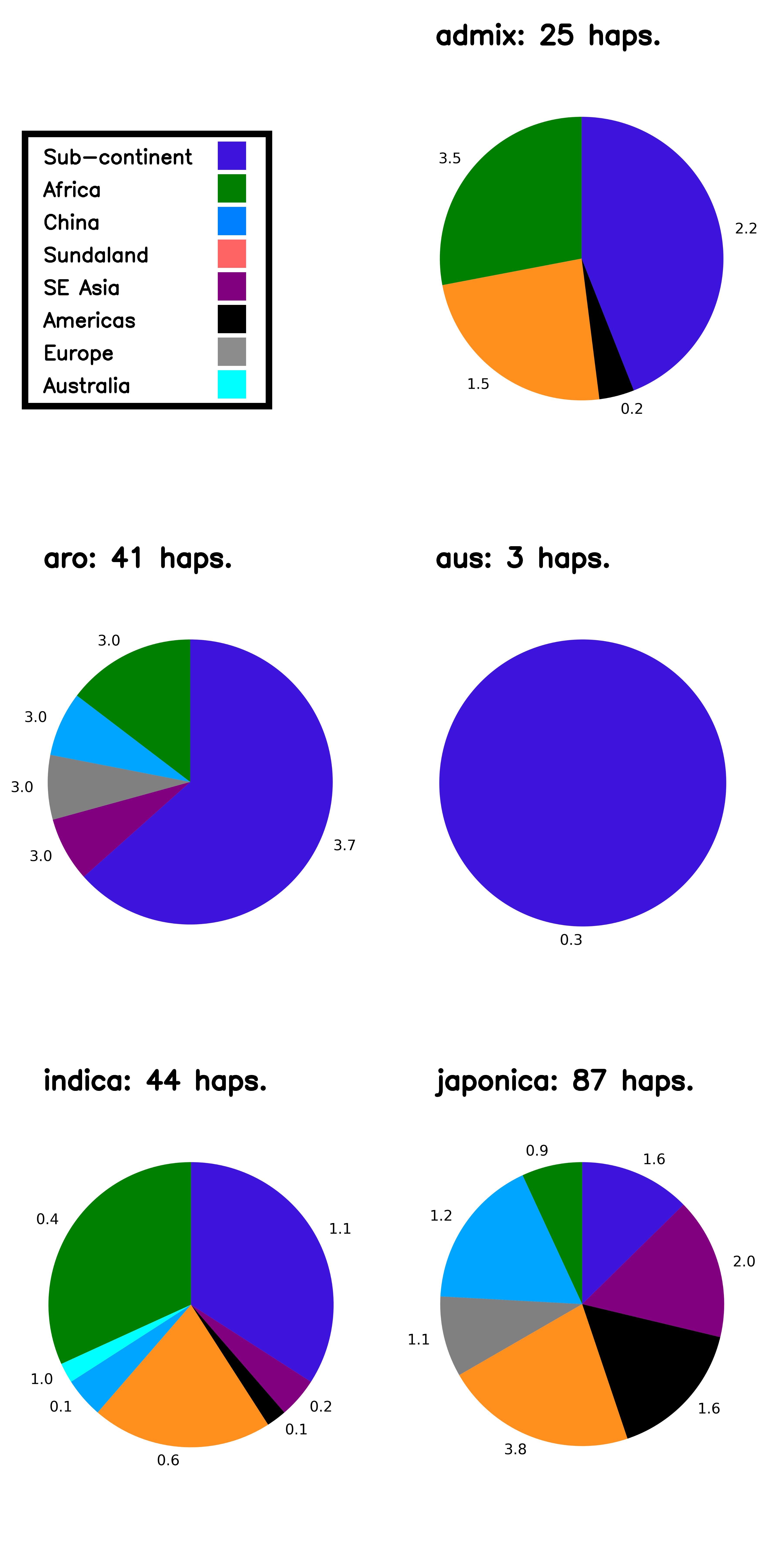


Figure S2p. IRRI database origins of low value associated haplotypes on chromosome 8.


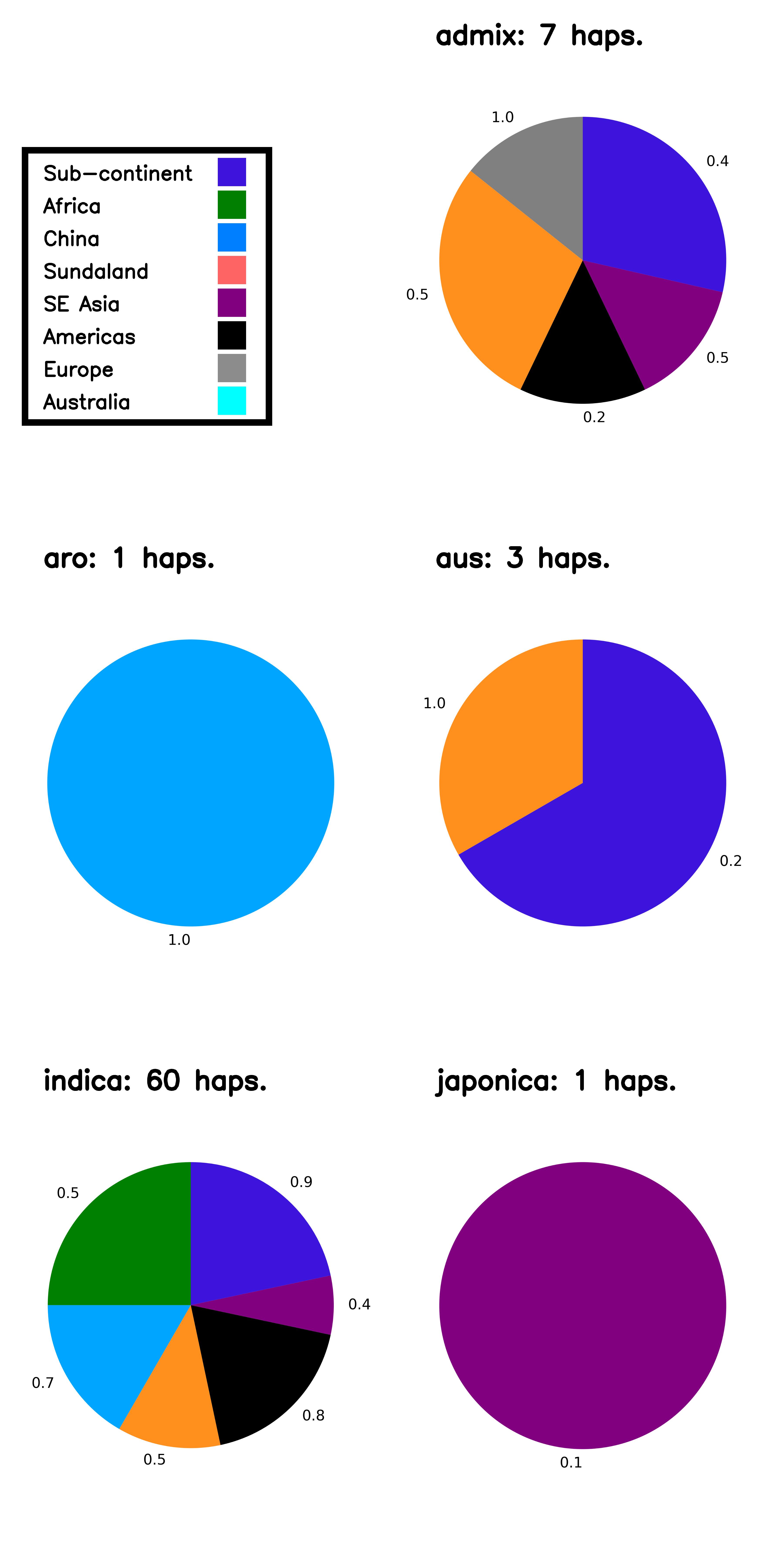


Figure S2q. IRRI database origins of high value associated haplotypes on chromosome 9.


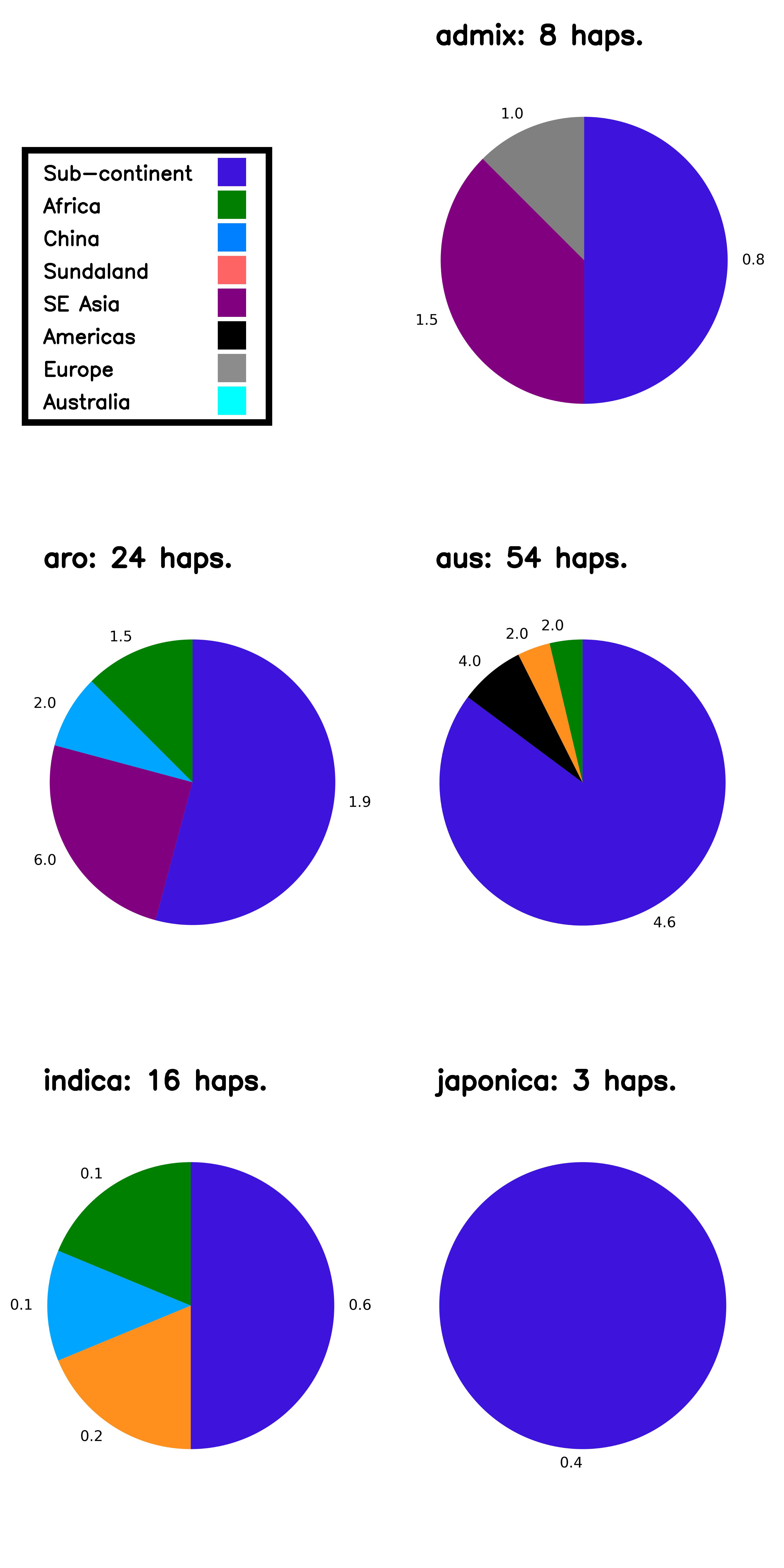


Figure S2r. IRRI database origins of low value associated haplotypes on chromosome 9.


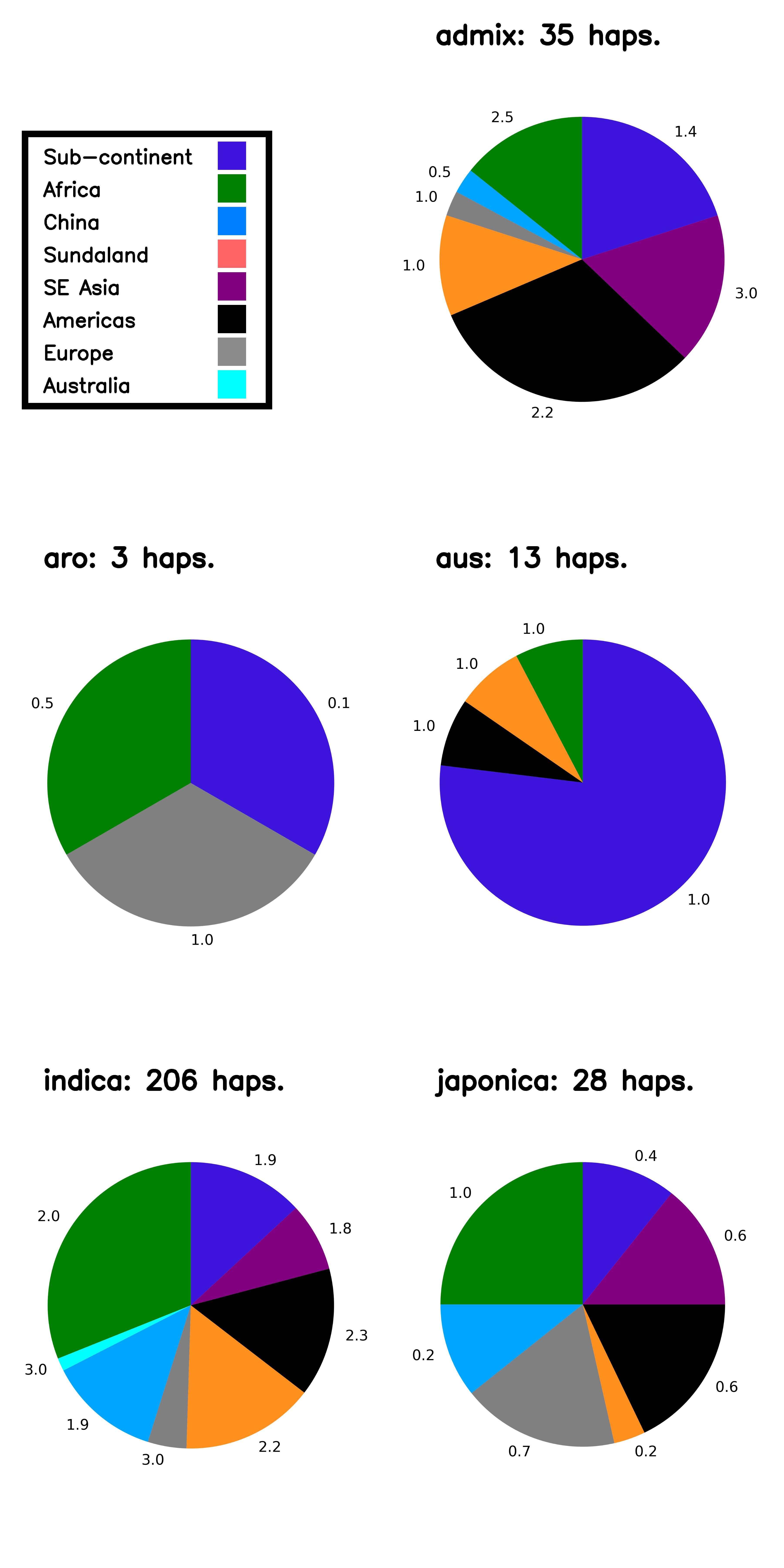


Figure S2s. IRRI database origins of high value associated haplotypes on chromosome 10.


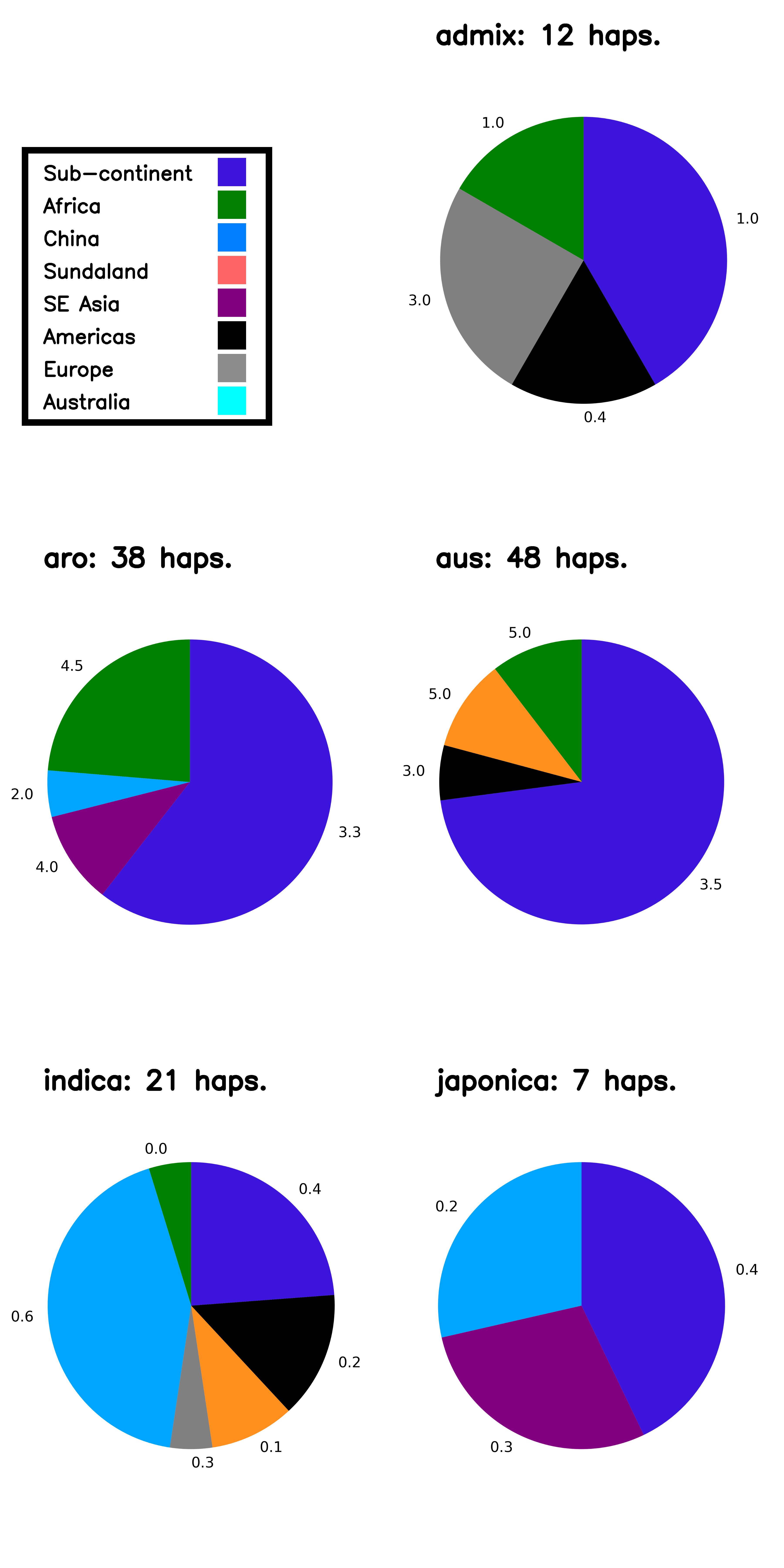


Figure S2t. IRRI database origins of low value associated haplotypes on chromosome 10.


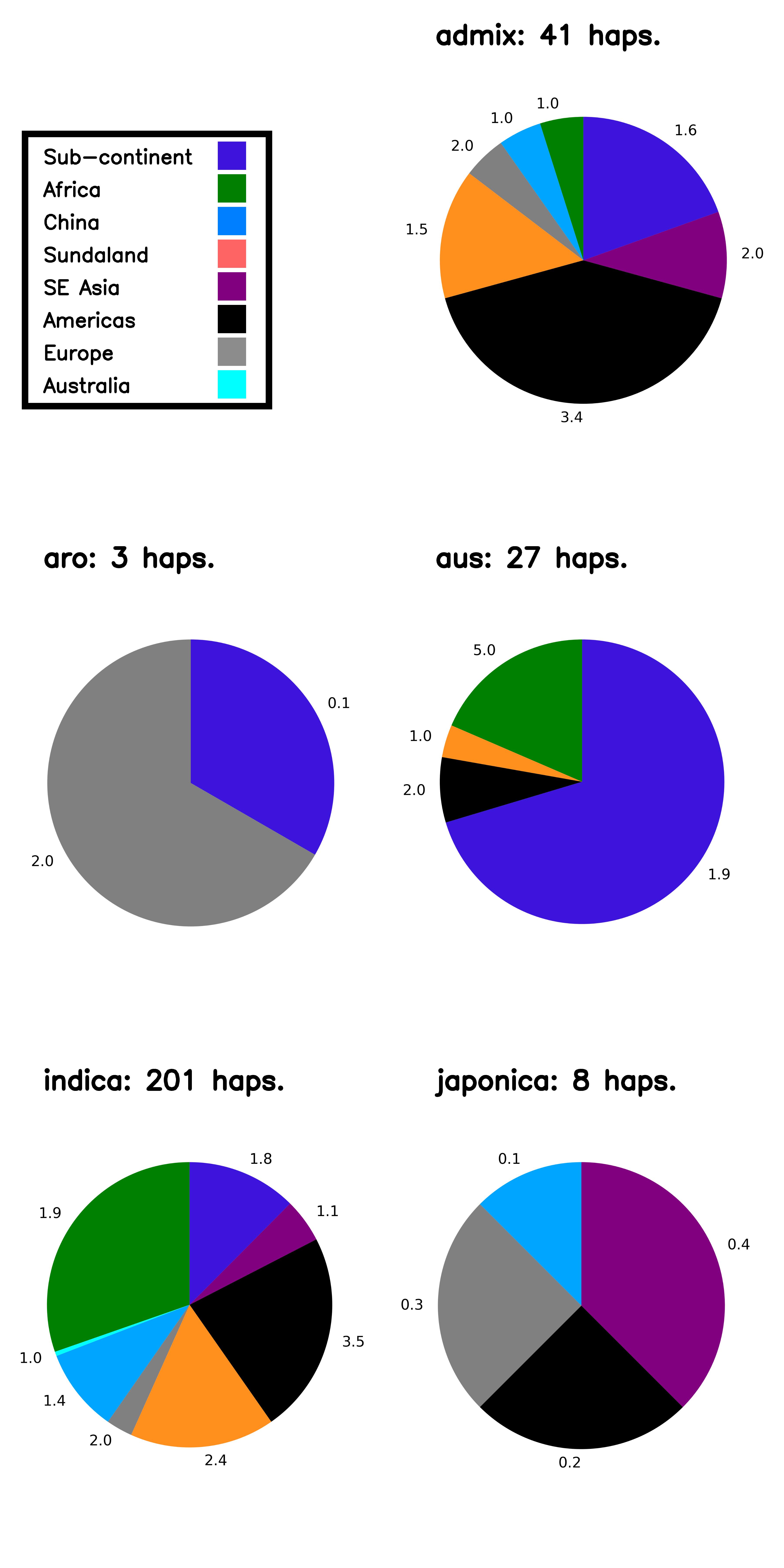


Figure S2u. IRRI database origins of high value associated haplotypes on chromosome 11.


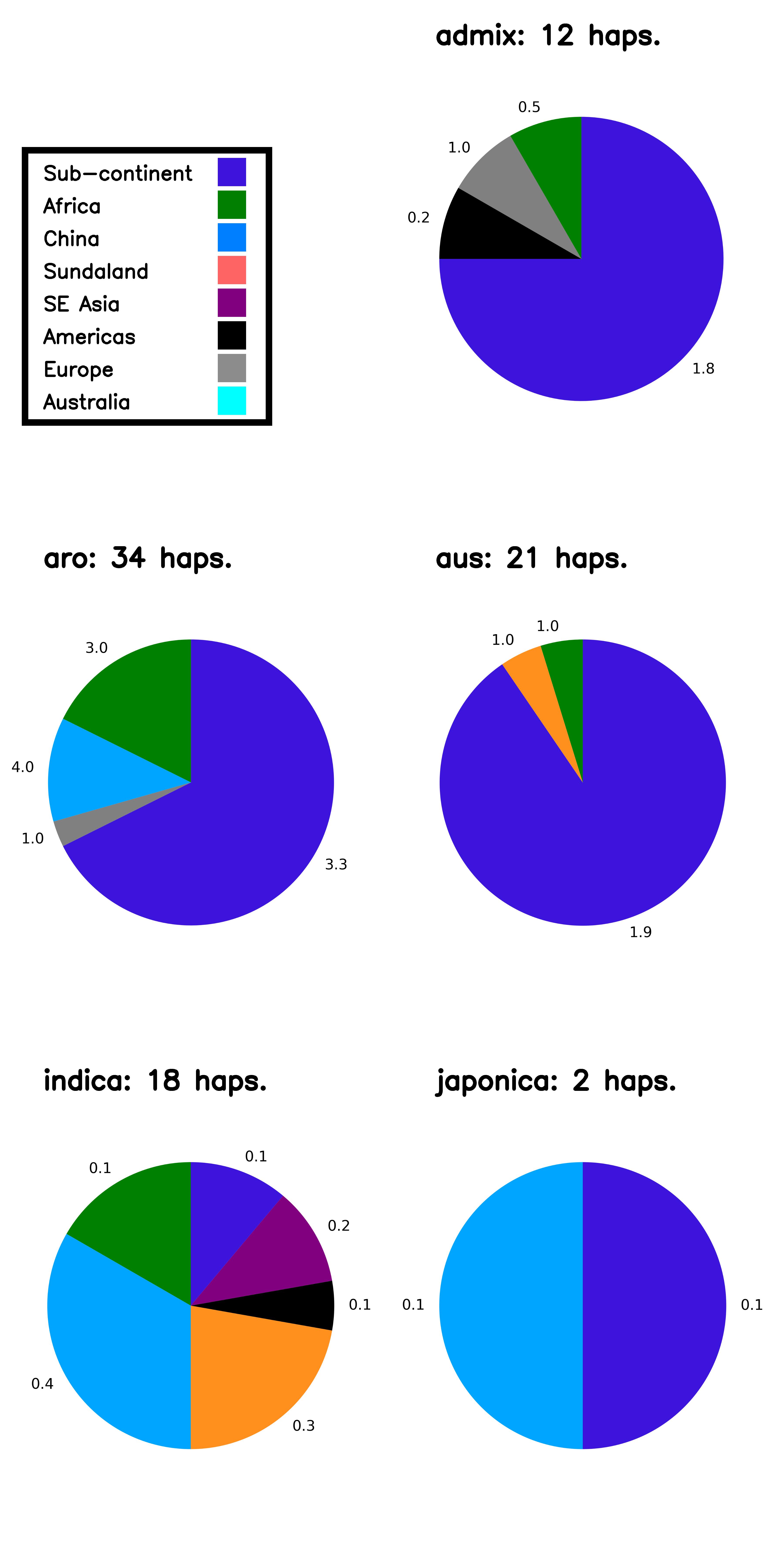


Figure S2v. IRRI database origins of low value associated haplotypes on chromosome 11.


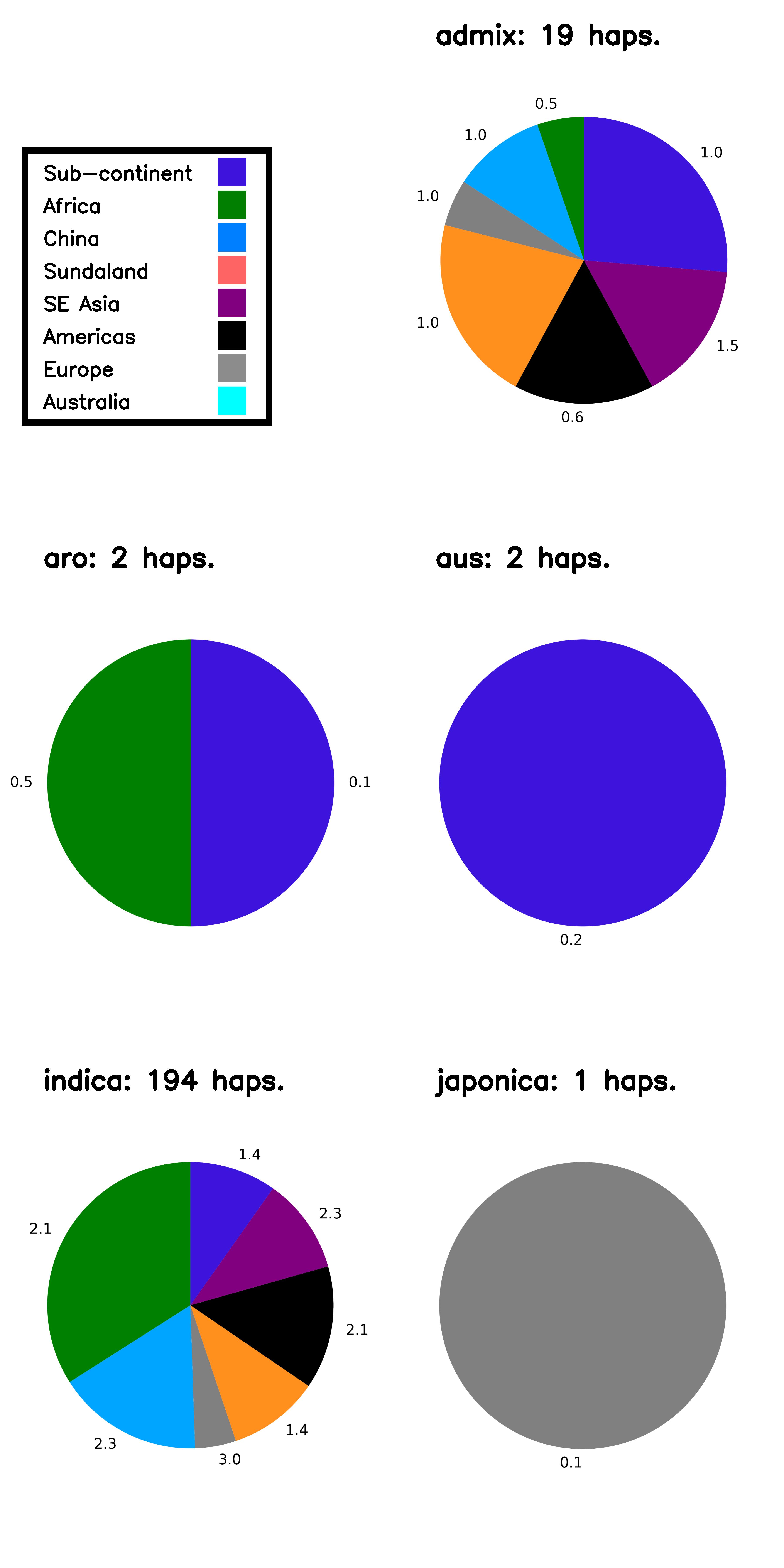


Figure S2w. IRRI database origins of high value associated haplotypes on chromosome 12.


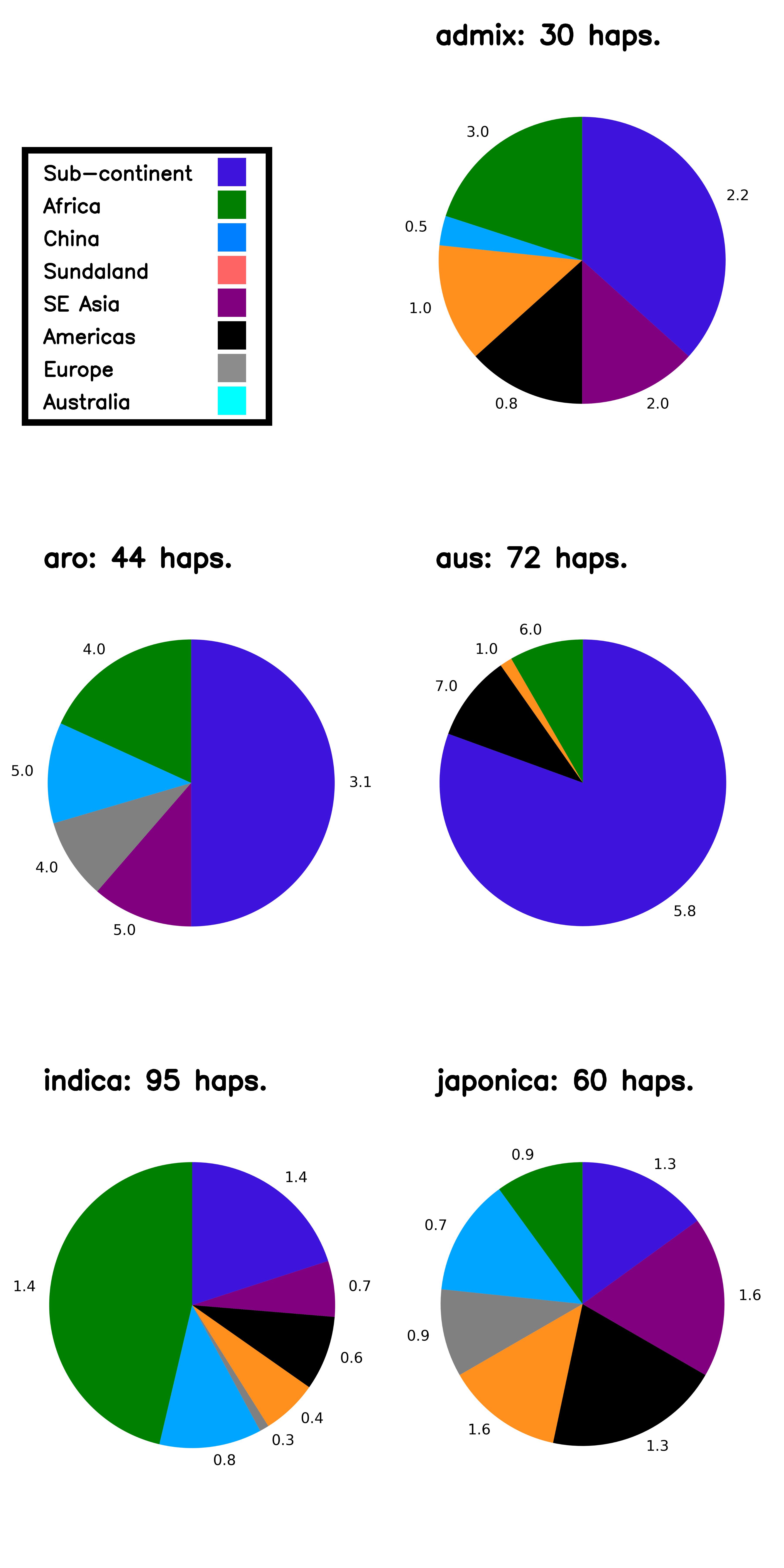


Figure S2x. IRRI database origins of low value associated haplotypes on chromosome 12.


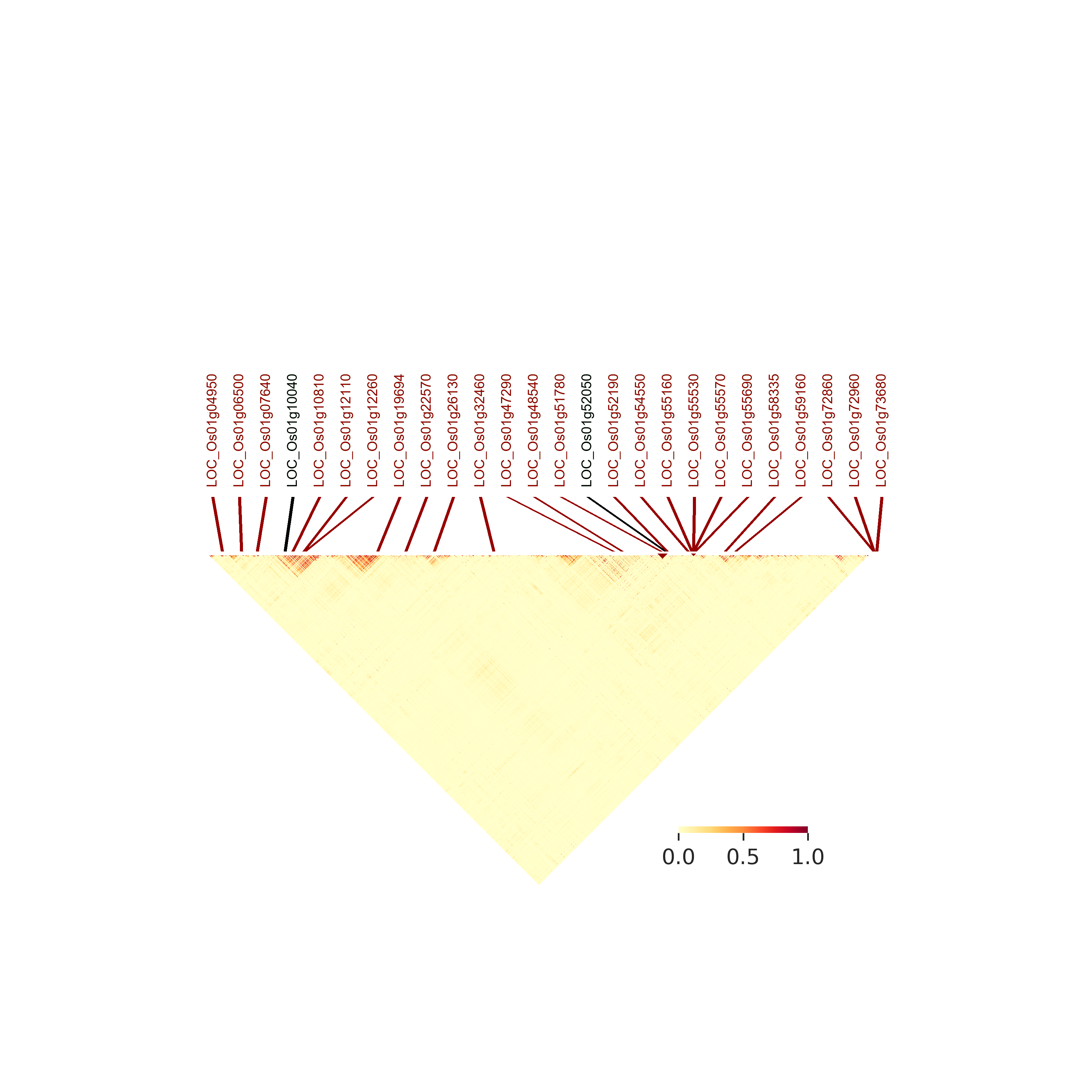
 Figure S3a. LD map of chromosome 1.


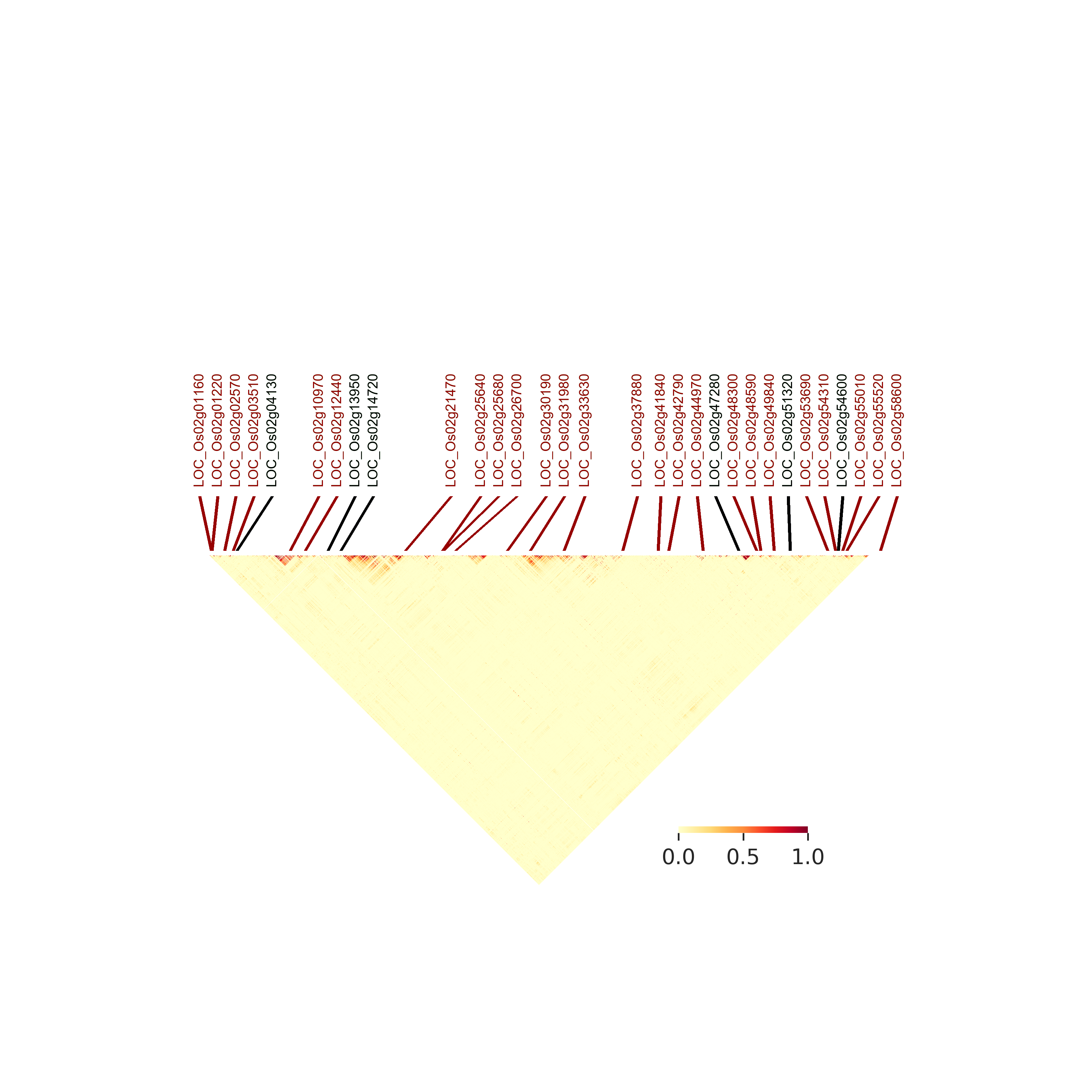
 Figure S3b. LD map of chromosome 2.


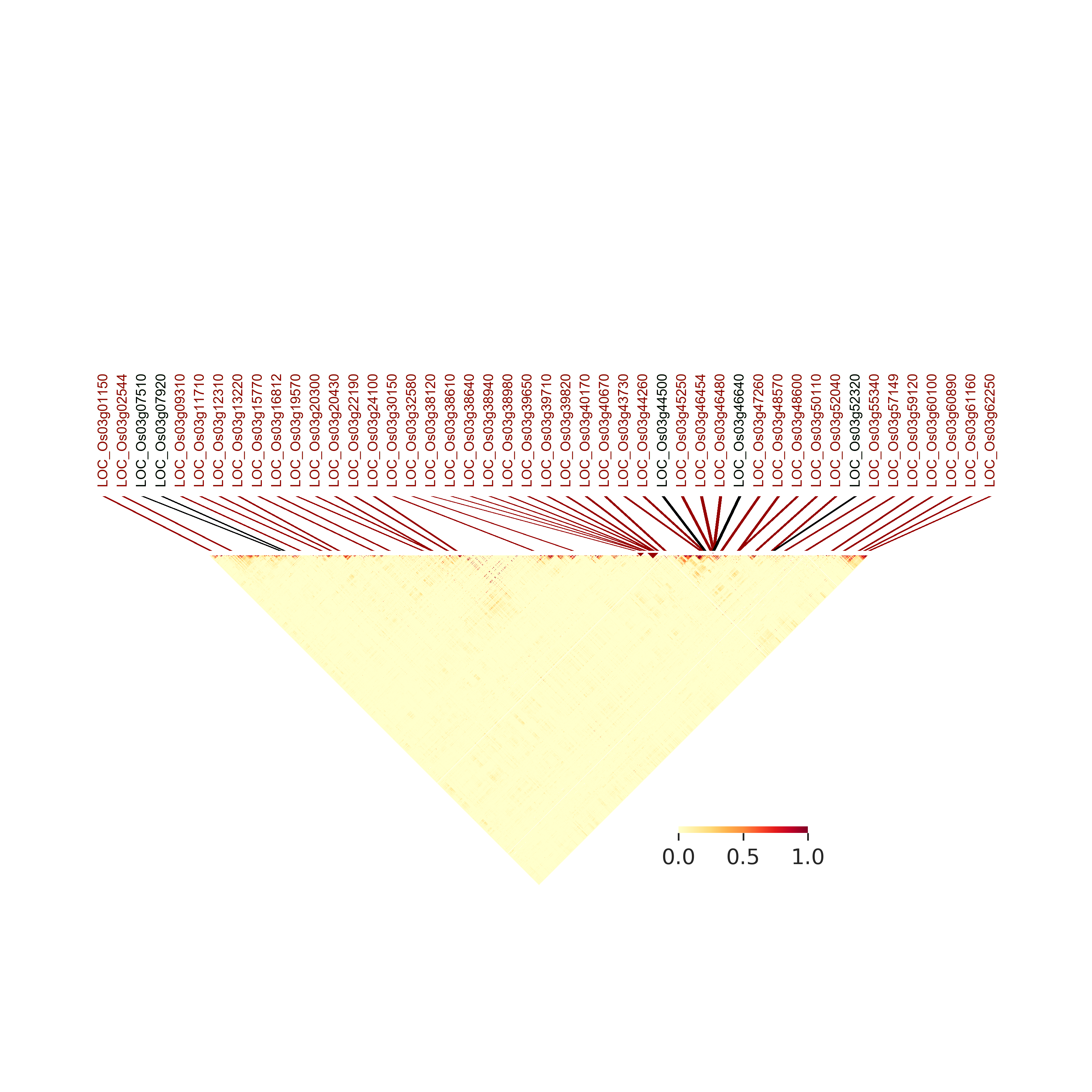
 Figure S3c. LD map of chromosome 3.


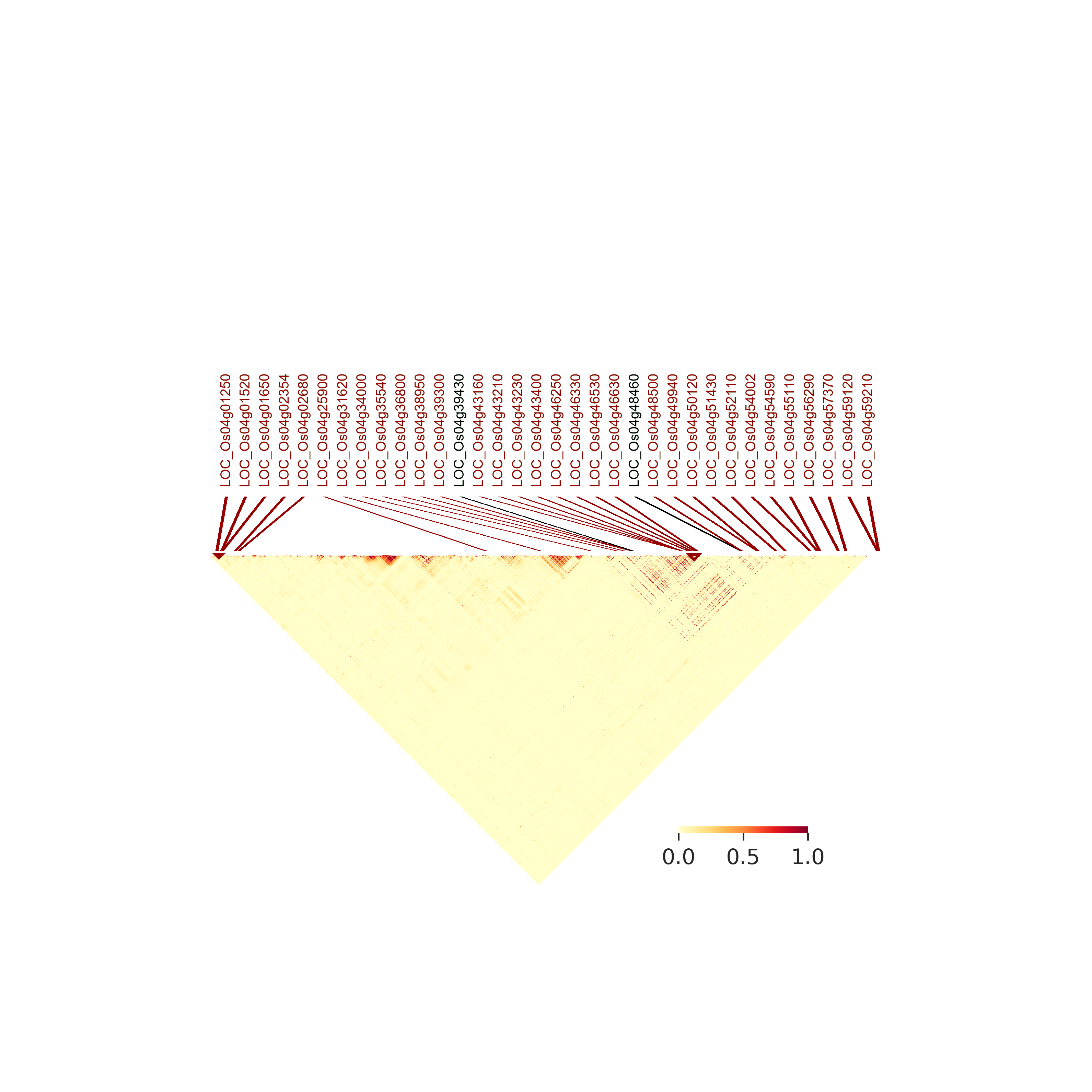
 Figure S3d. LD map of chromosome 4.


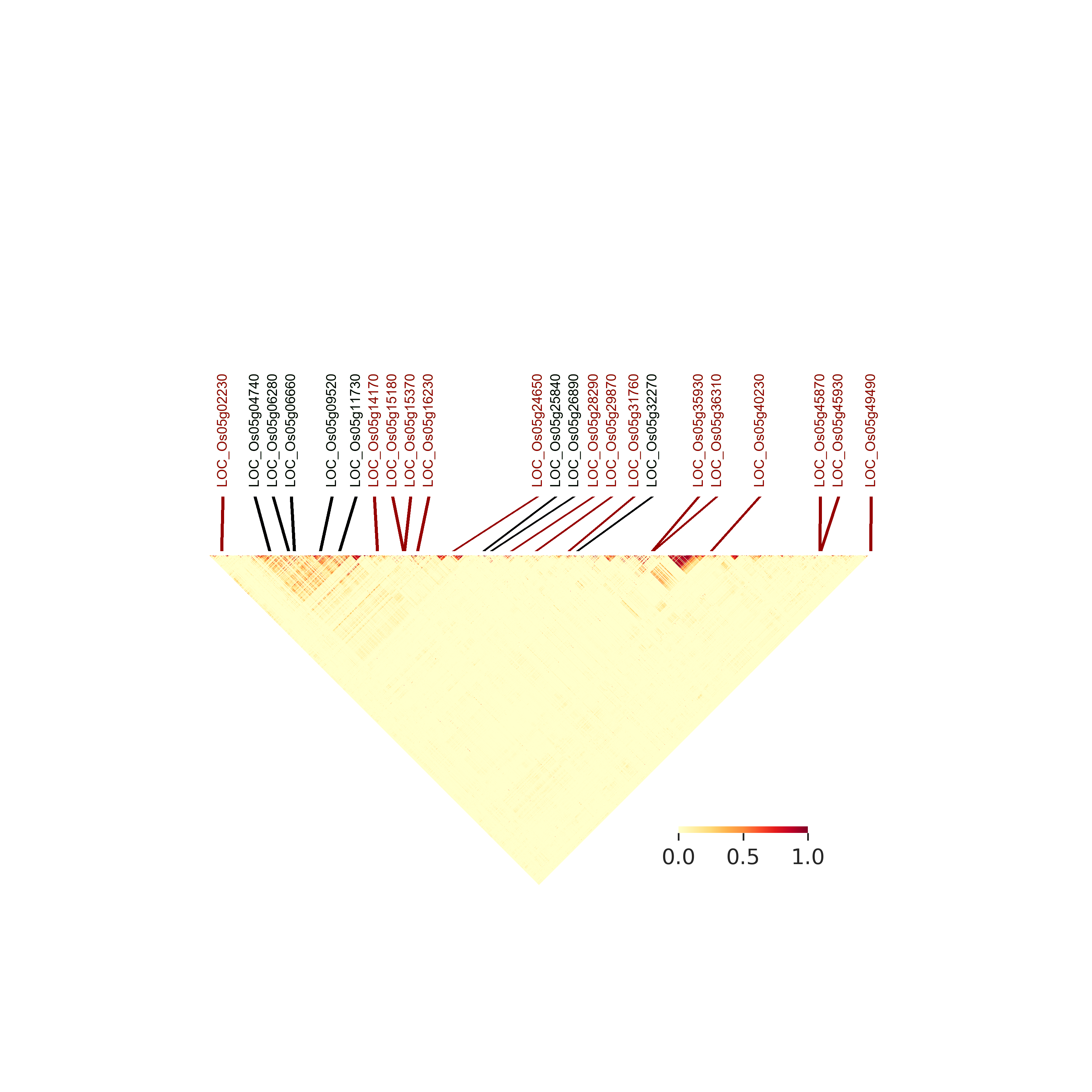
 Figure S3e. LD map of chromosome 5.


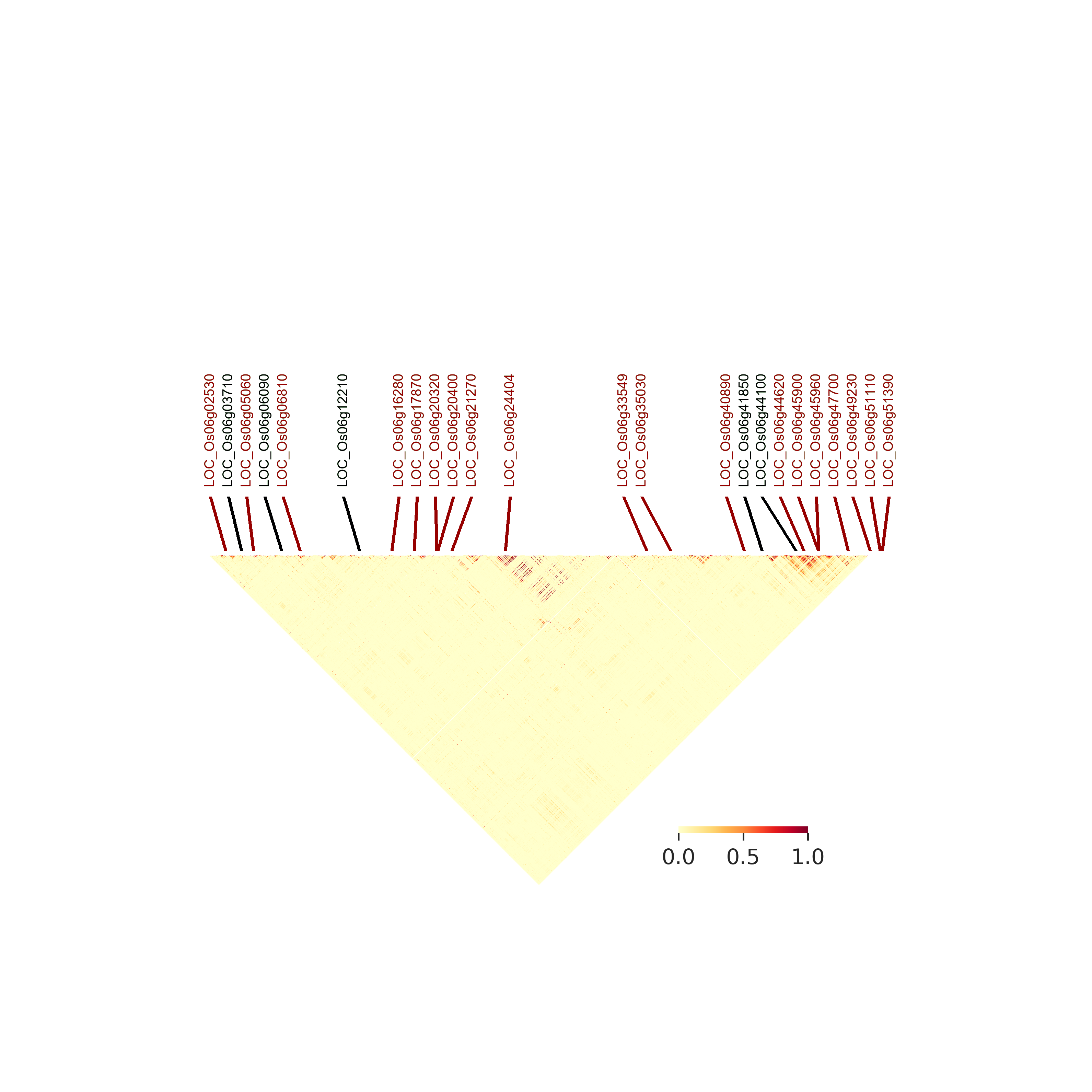
 Figure S3f. LD map of chromosome 6.


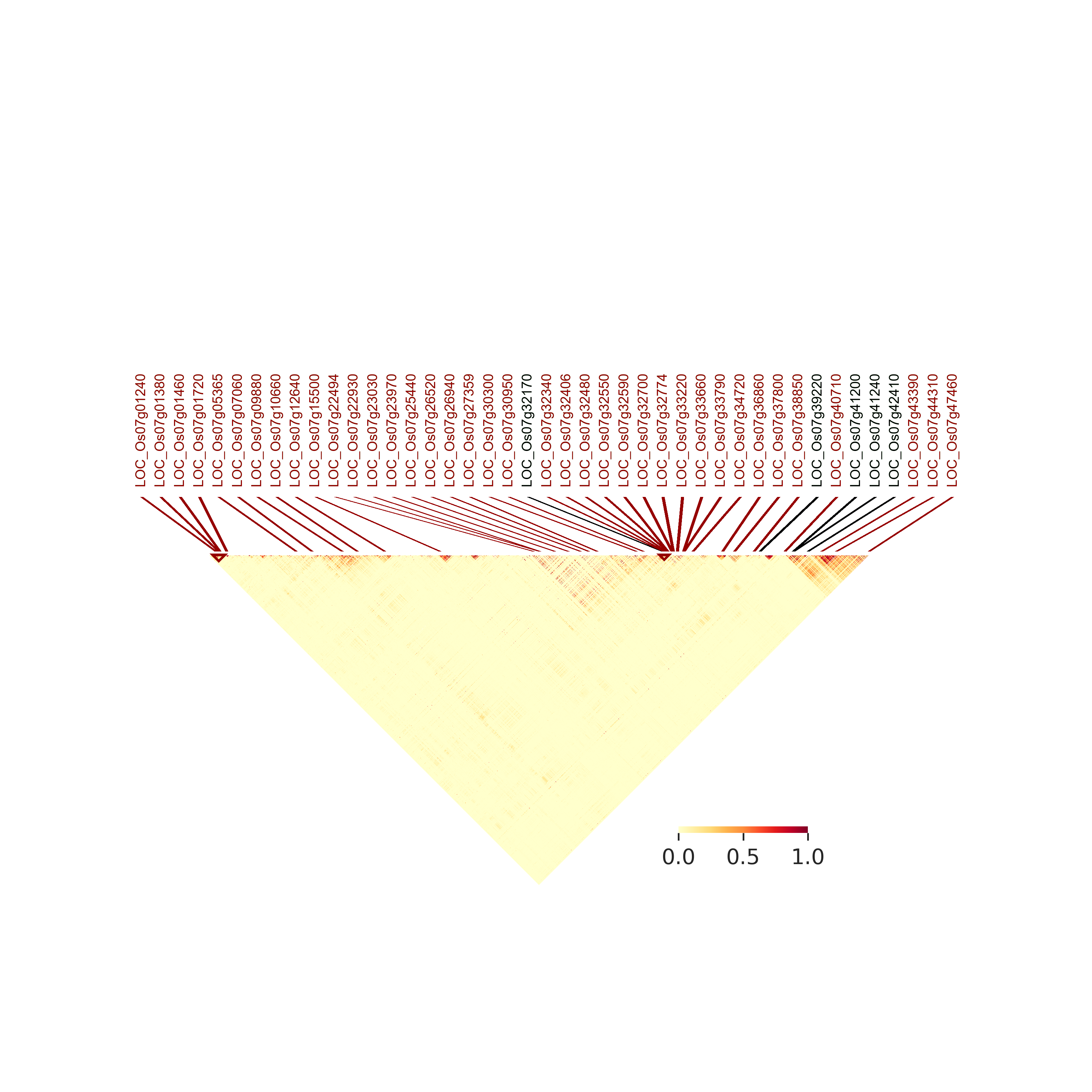
 Figure S3g. LD map of chromosome 7.


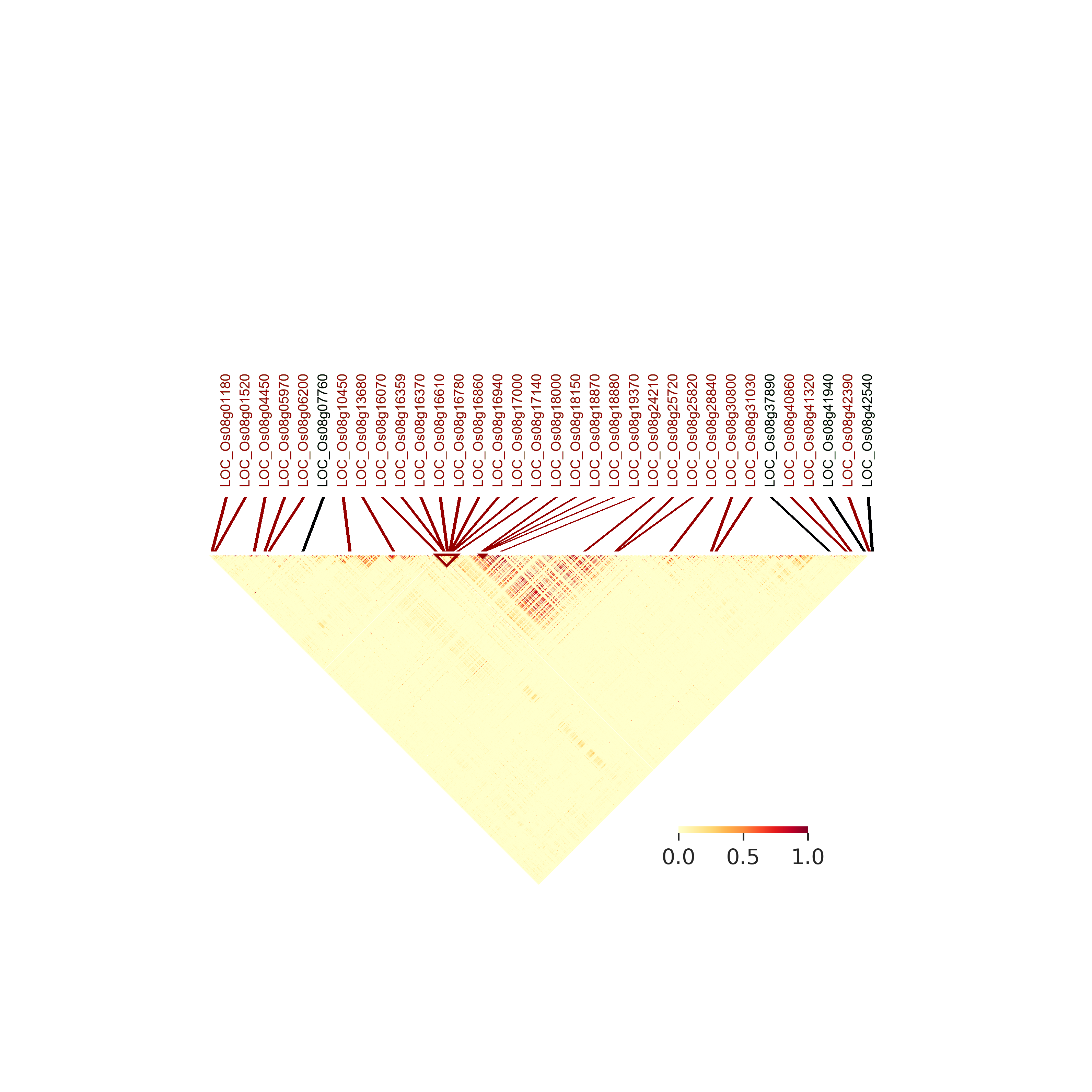
 Figure S3h. LD map of chromosome 8.


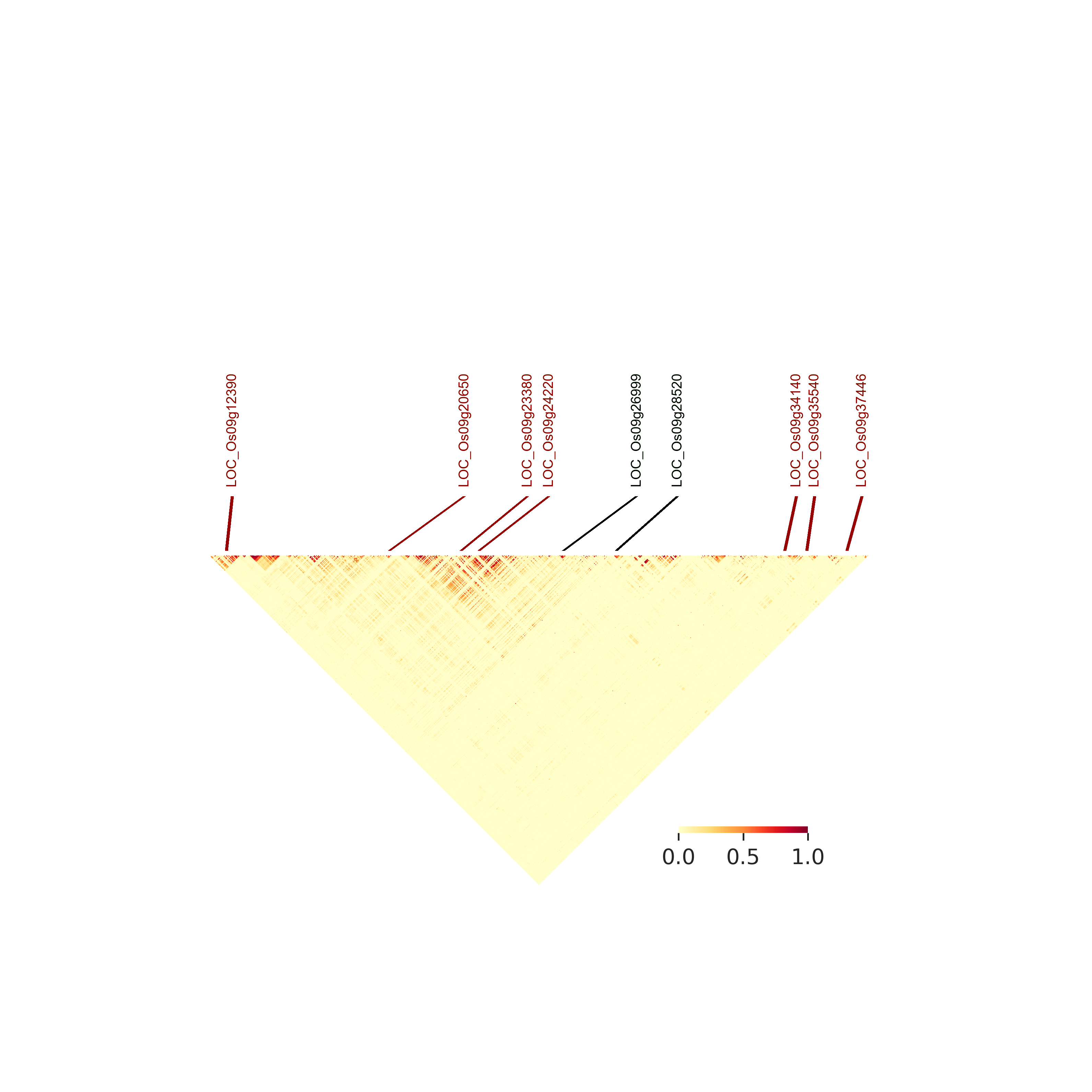
 Figure S3i. LD map of chromosome 9.


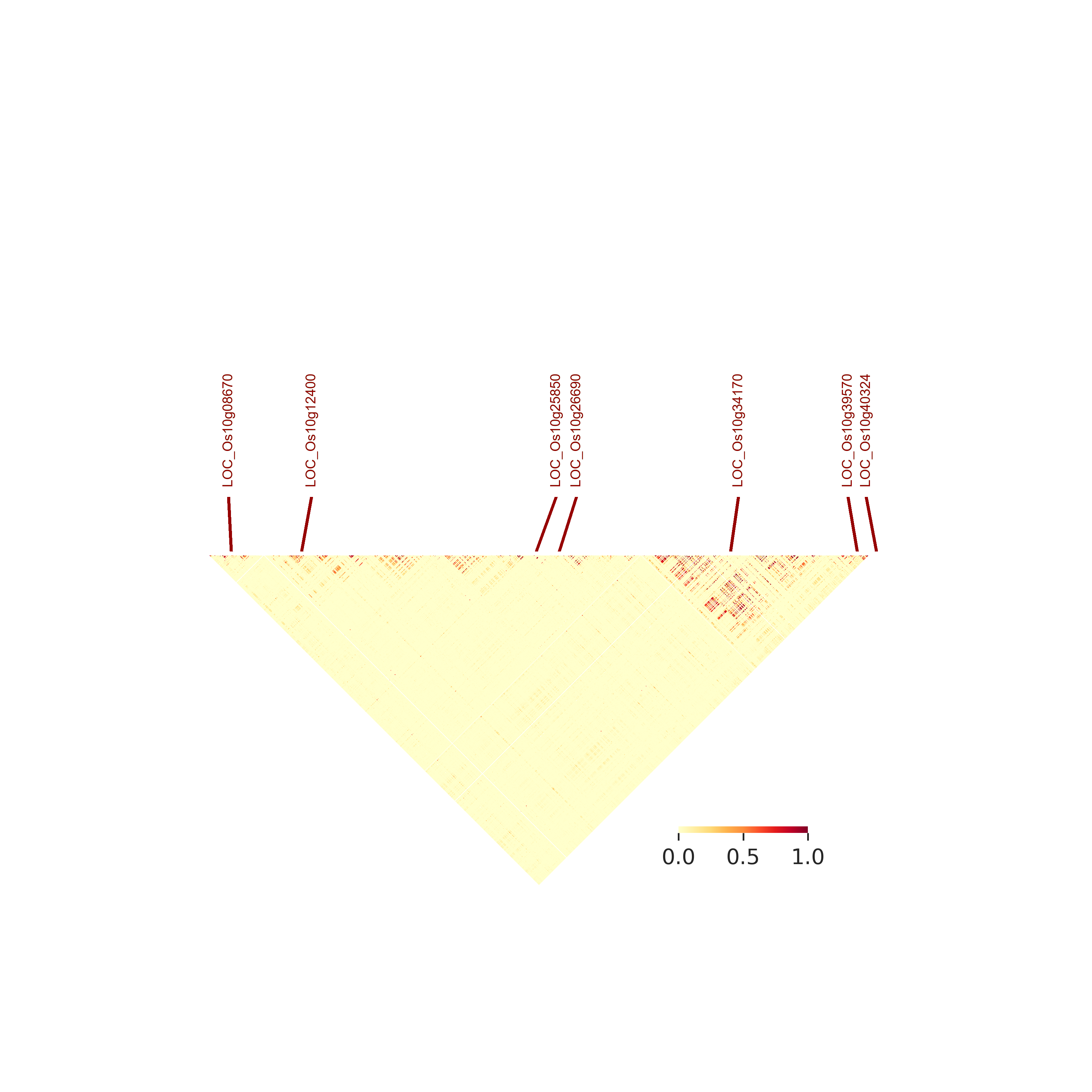
 Figure S3j. LD map of chromosome 10.


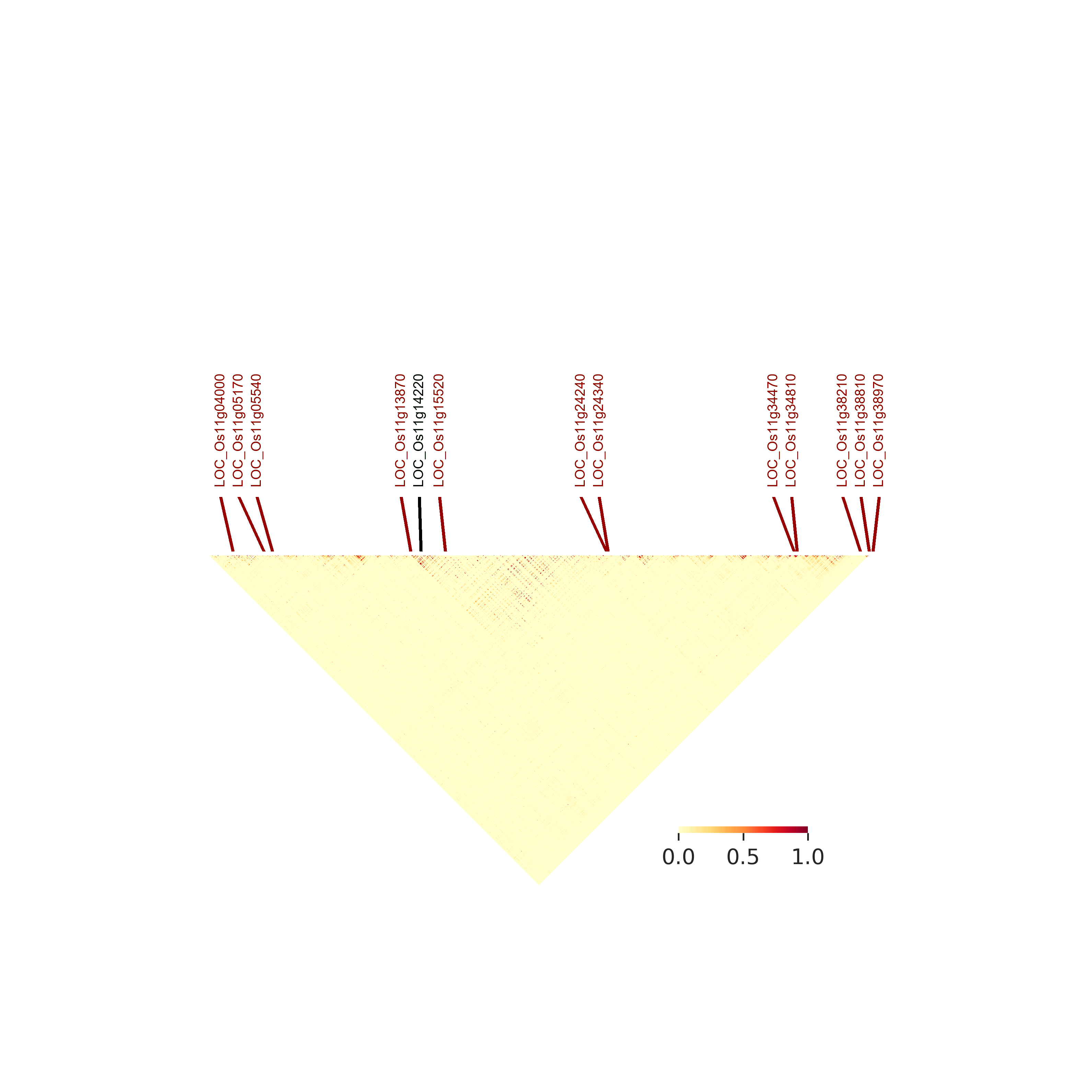
 Figure S3k. LD map of chromosome 11.


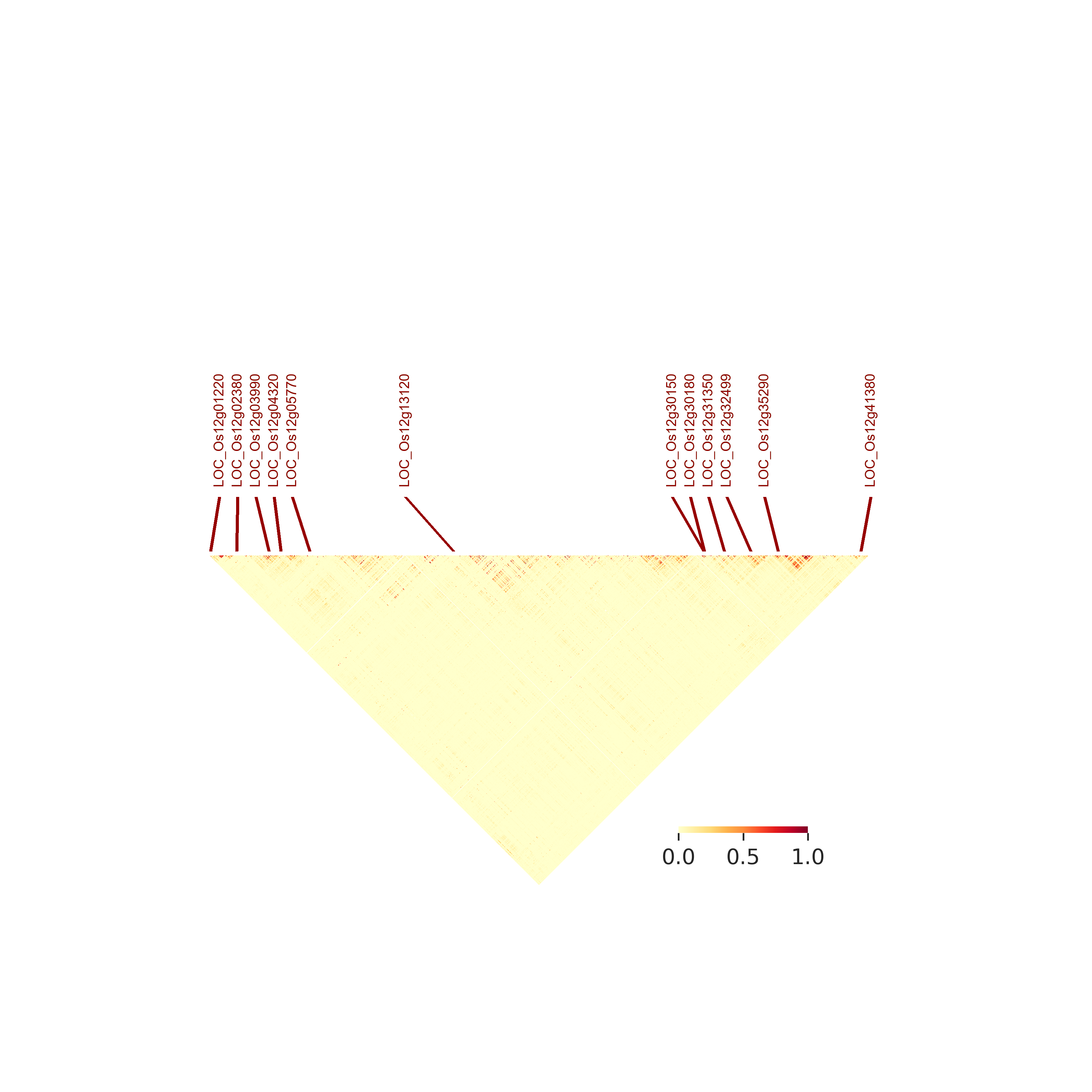
 Figure S3l. LD map of chromosome 12.


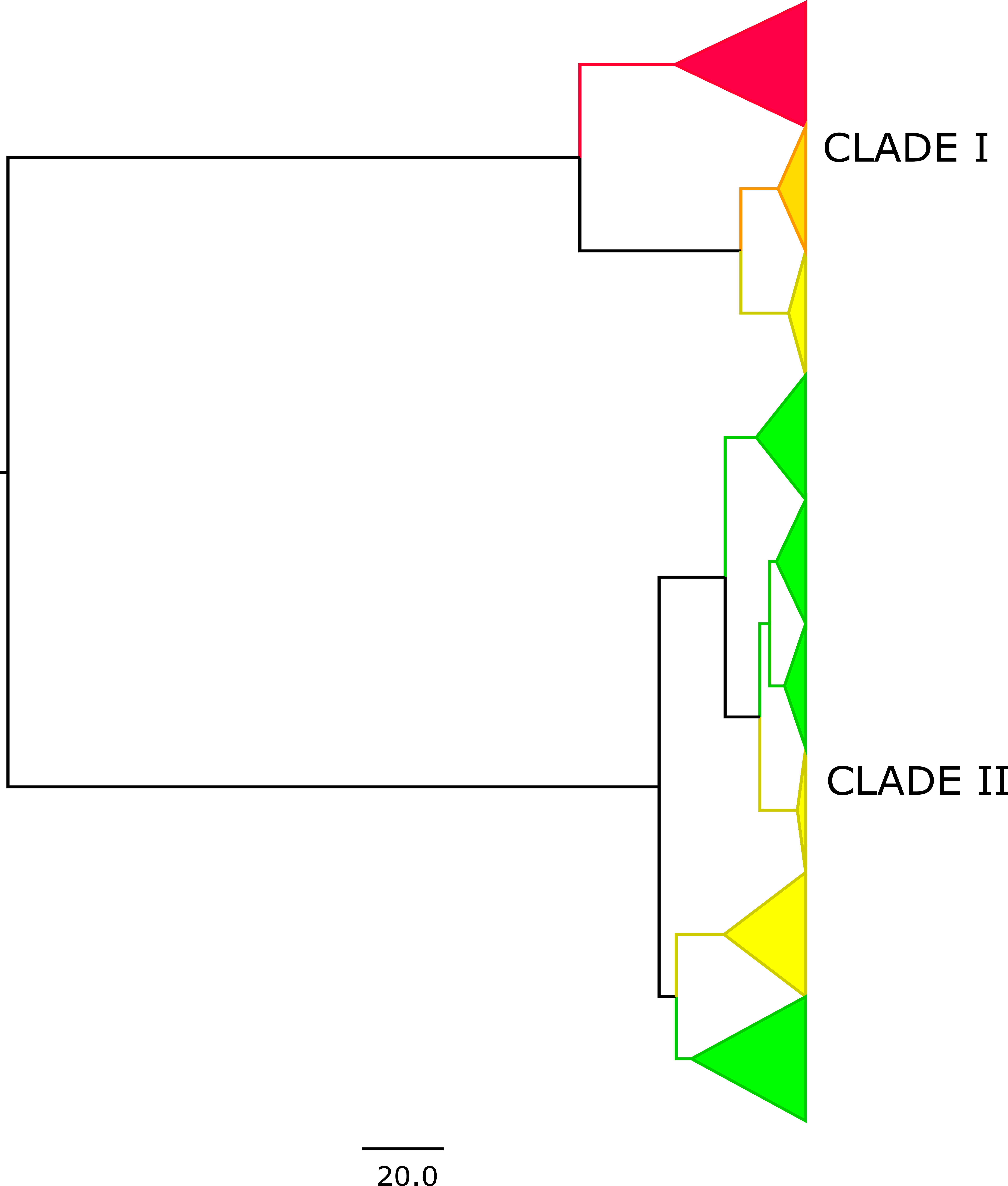


Figure S4a.


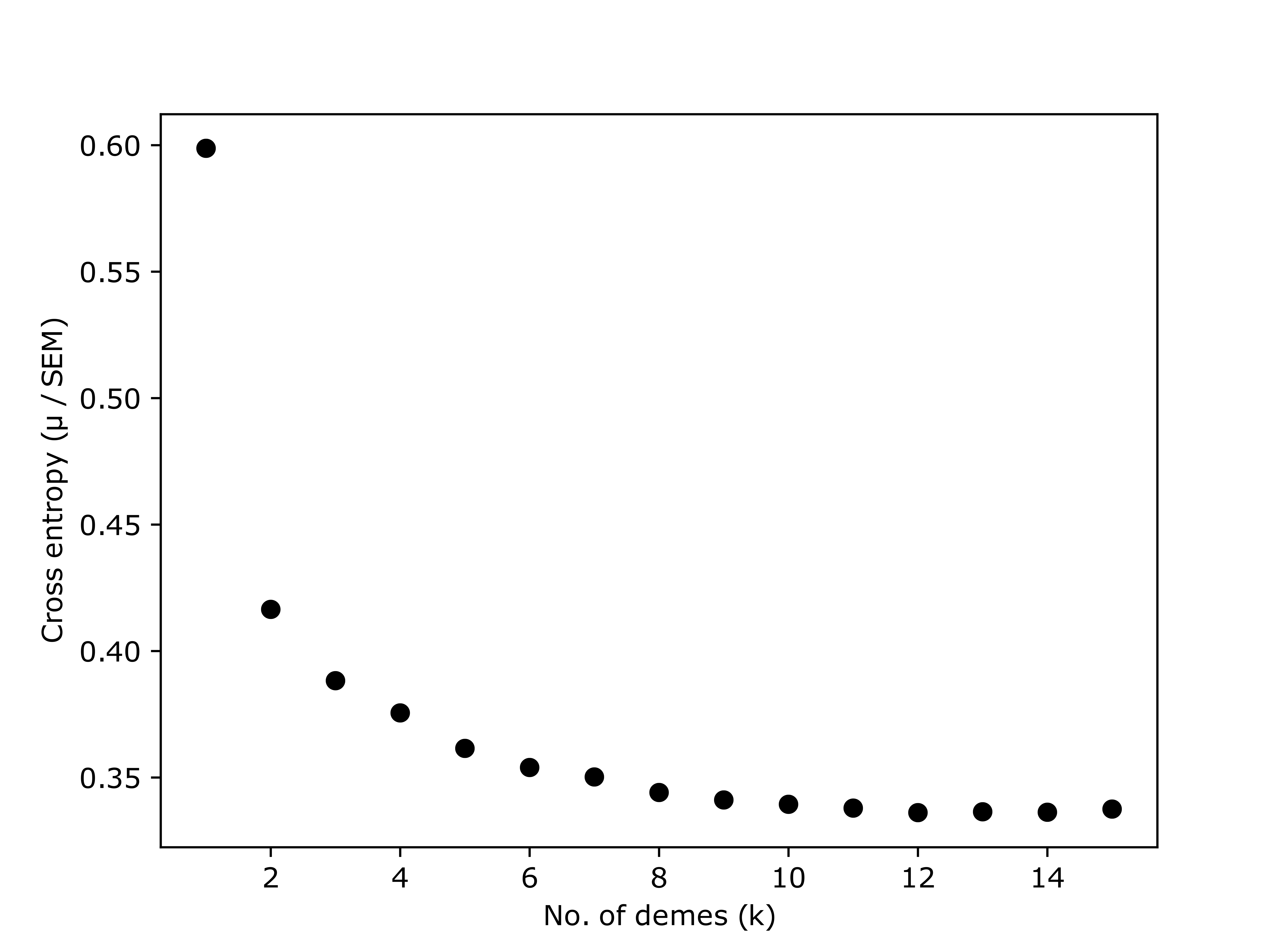


Figure S5.


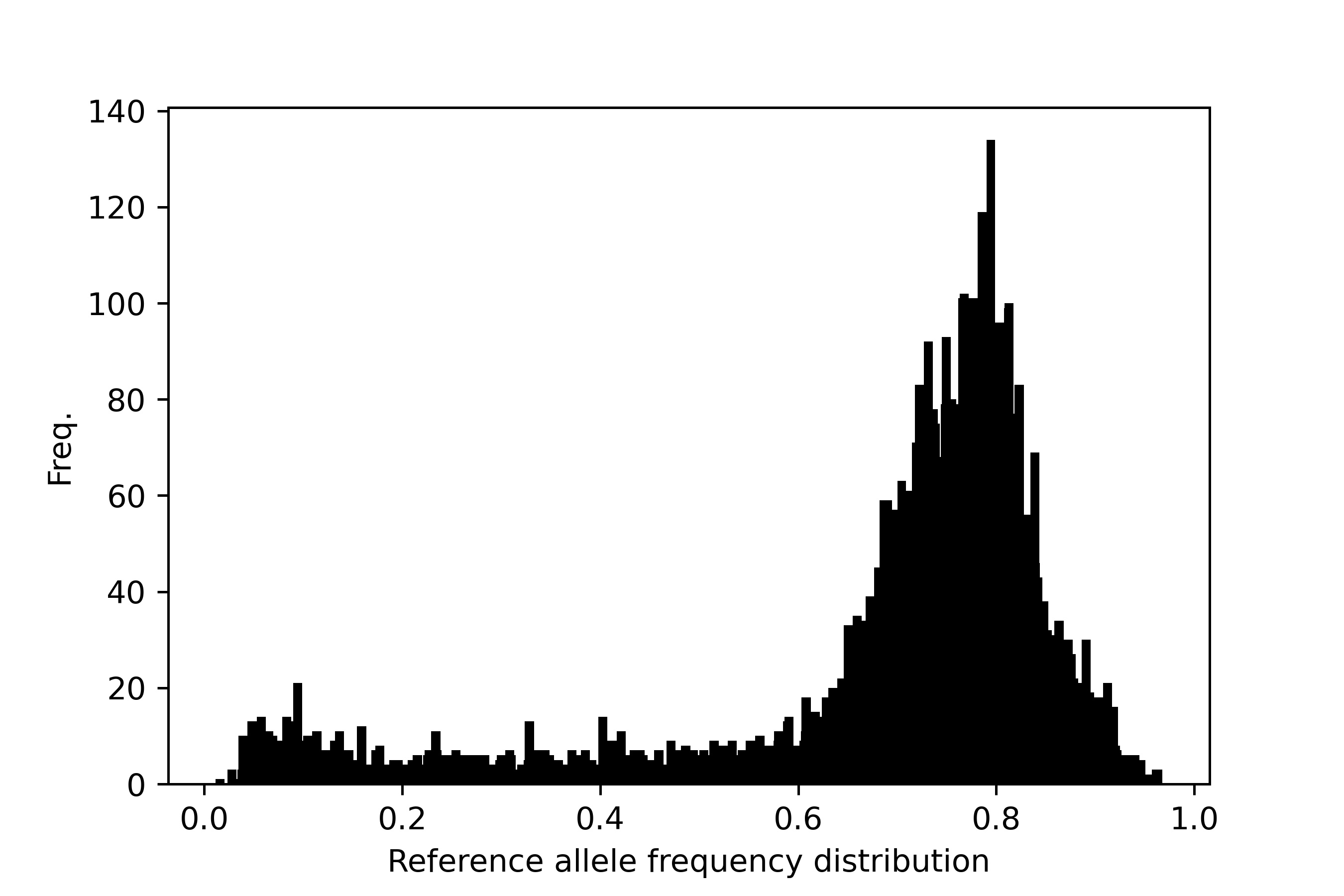
 Figure S6a. Site-frequency spectrum for Chromosome 10.


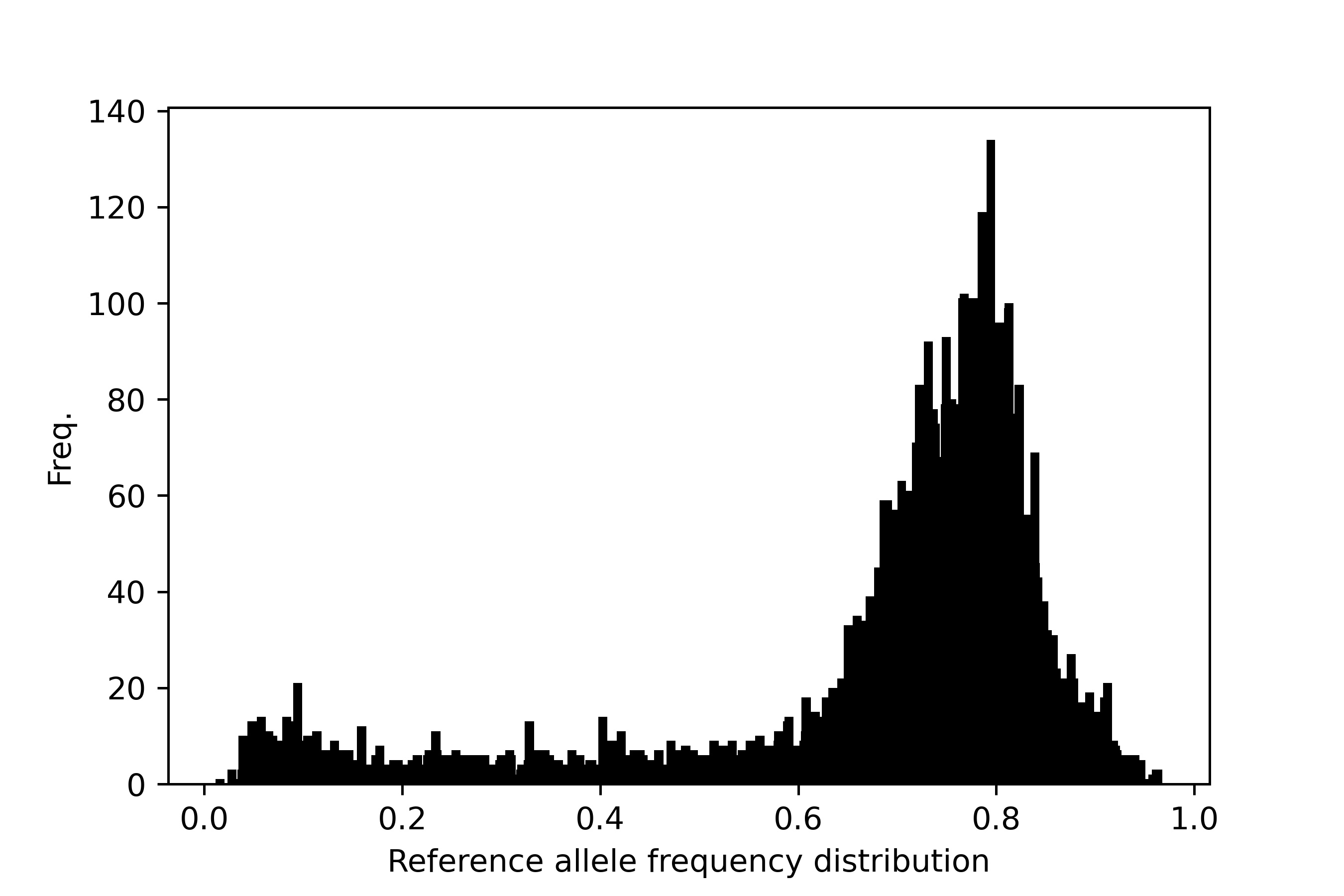
 Figure S6b. Site-frequency spectrum for Chromosome 11.


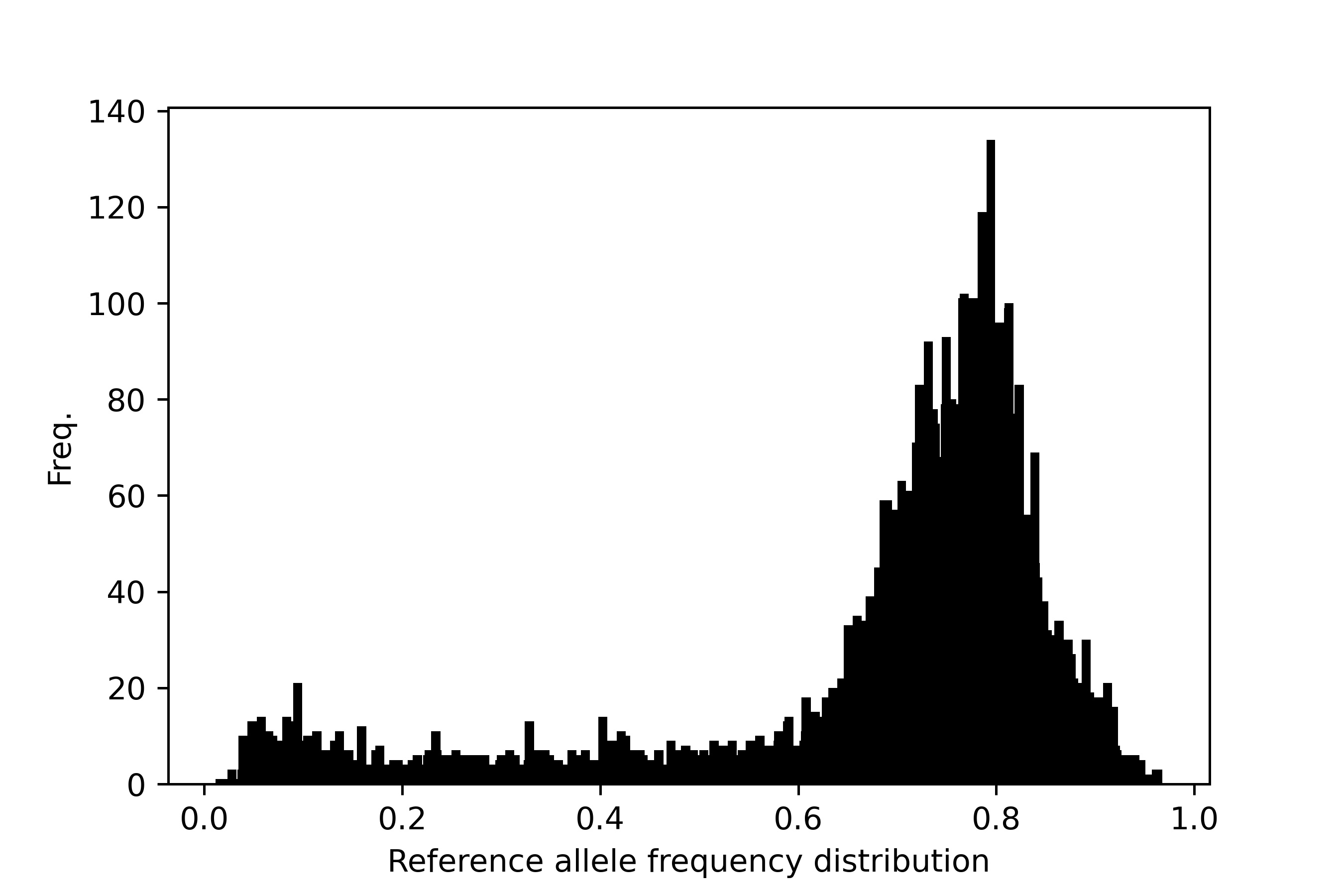
 Figure S6c. Site-frequency spectrum for Chromosome 12.


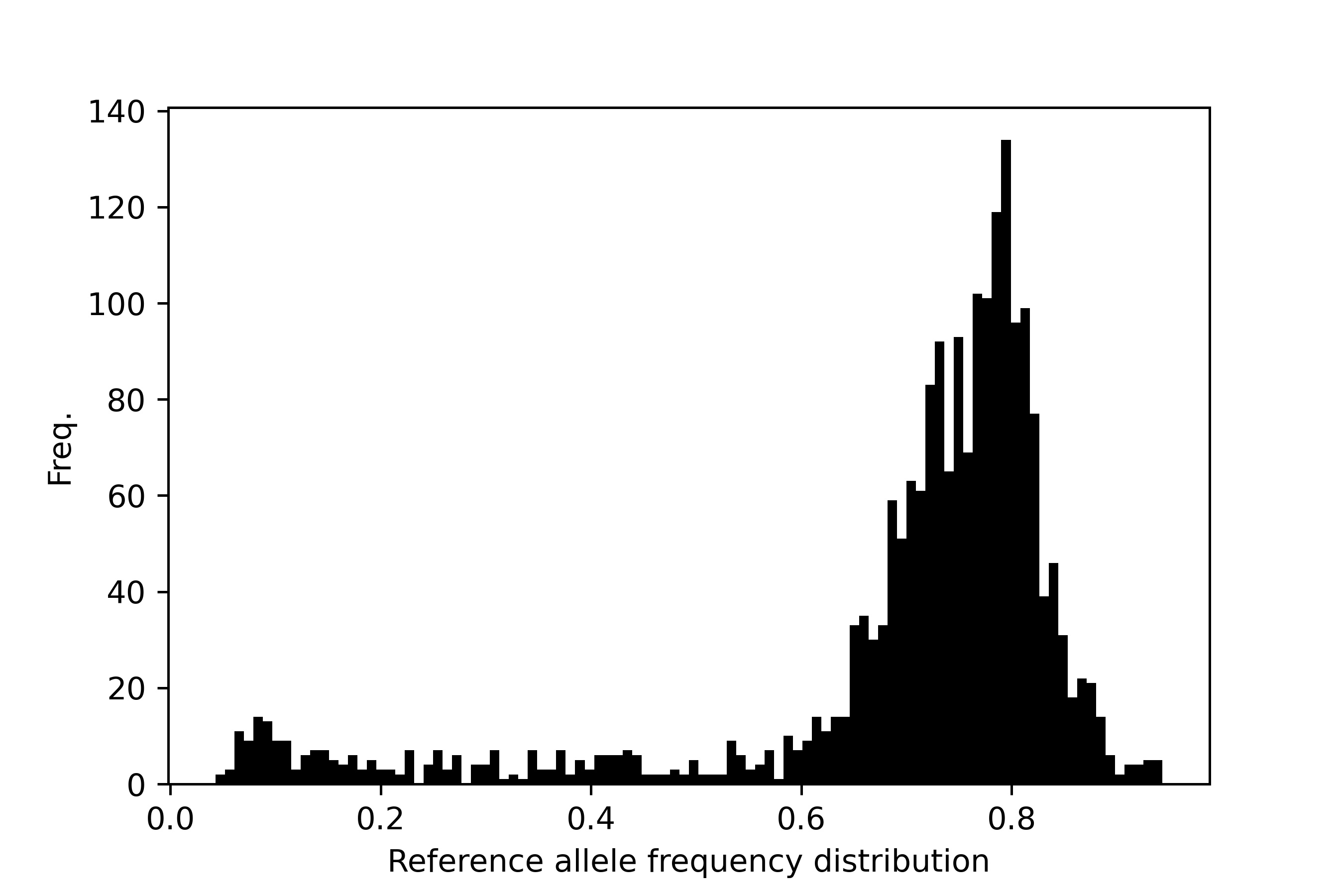
 Figure S6d. Site-frequency spectrum for Chromosome 1.


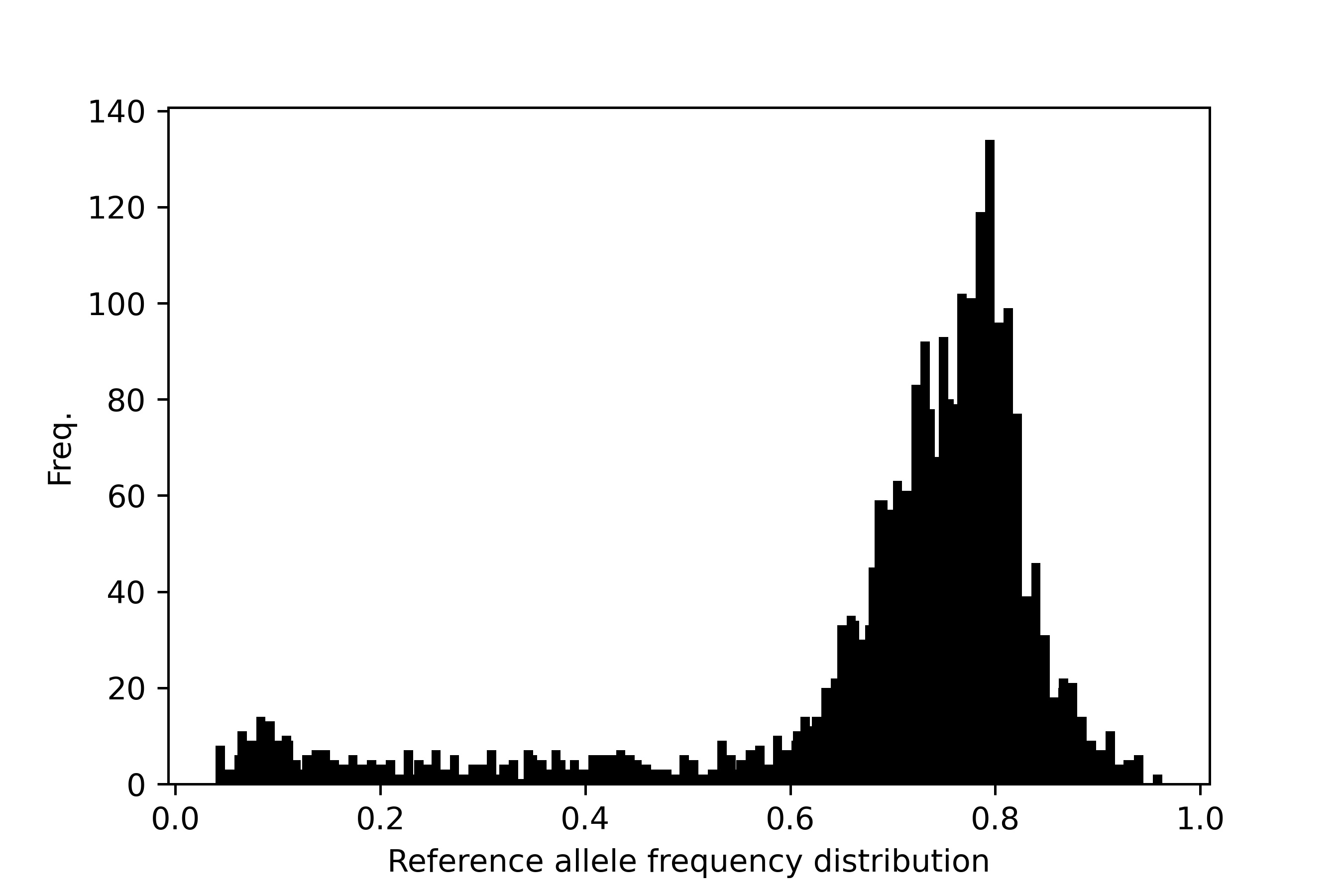
 Figure S6e. Site-frequency spectrum for Chromosome 2.


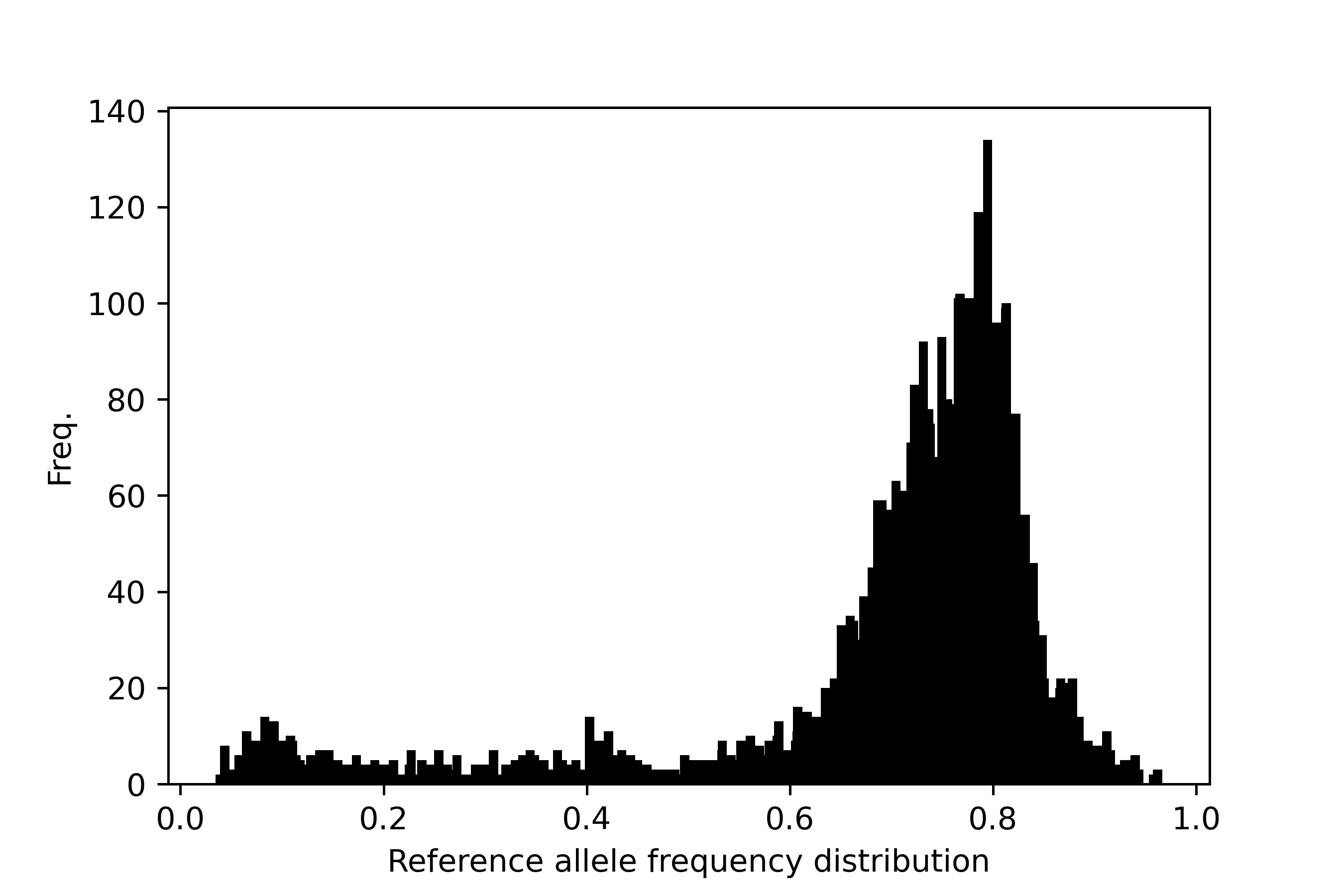
 Figure S6f. Site-frequency spectrum for Chromosome 3.


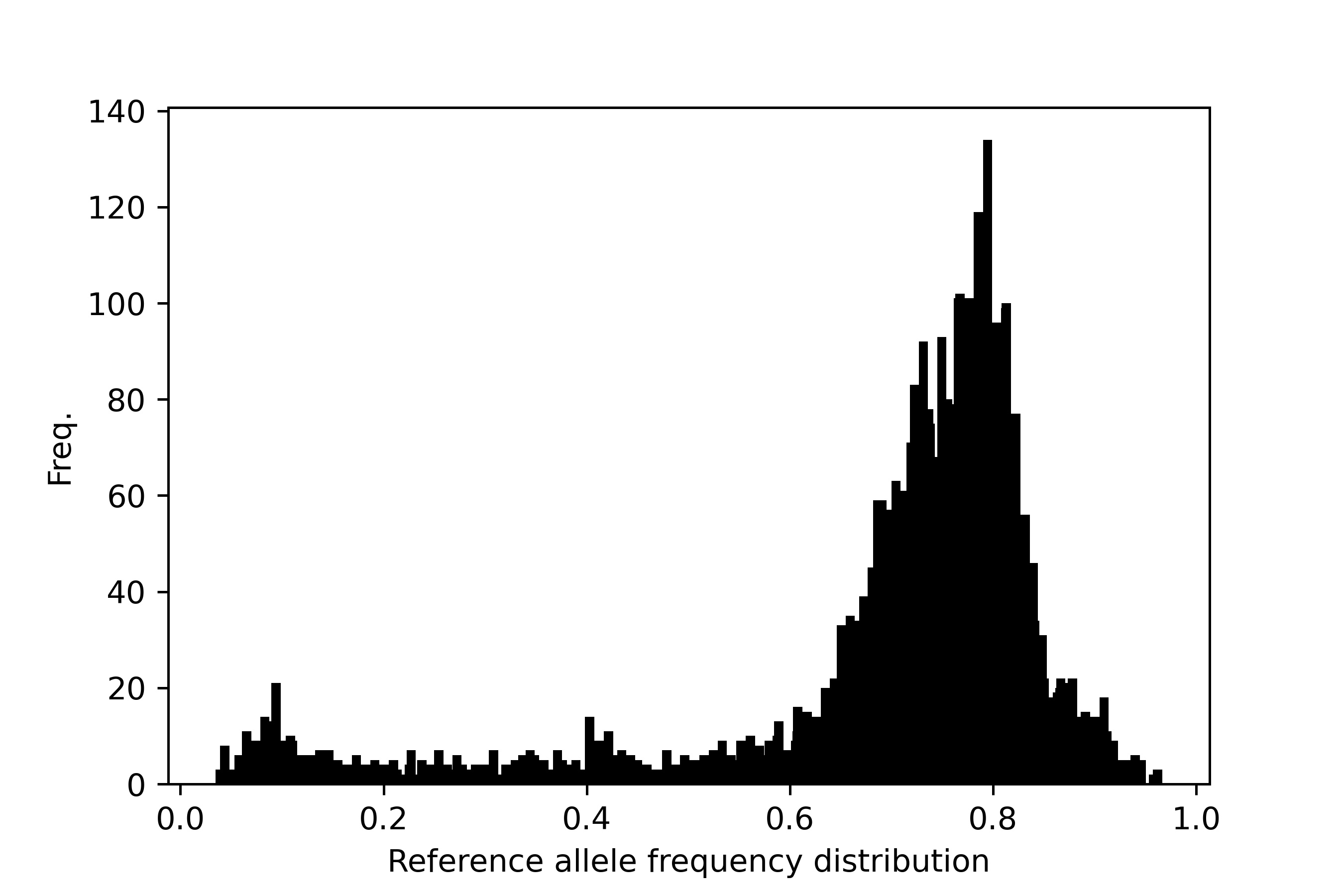
 Figure S6g. Site-frequency spectrum for Chromosome 4.


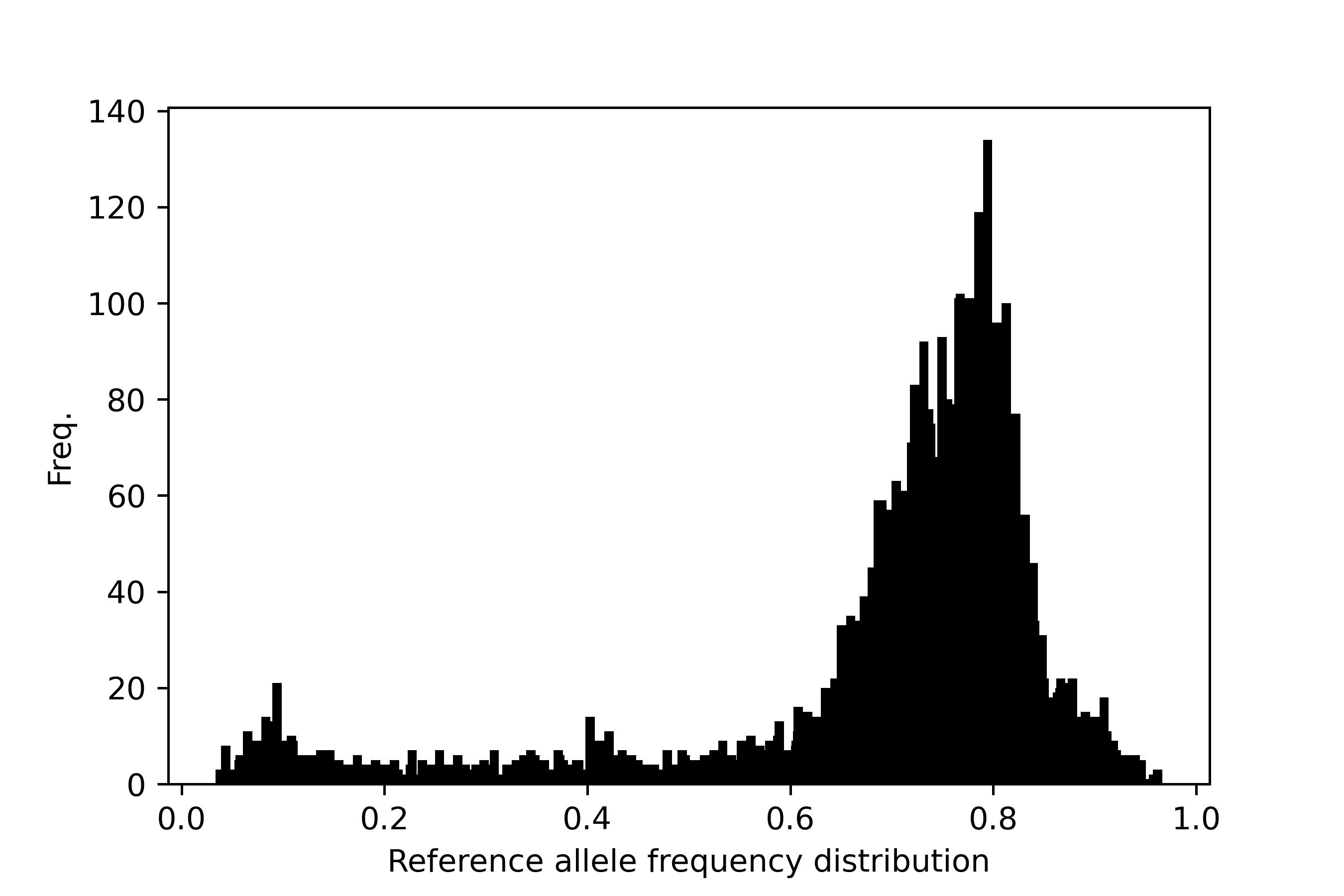
 Figure S6h. Site-frequency spectrum for Chromosome 5.


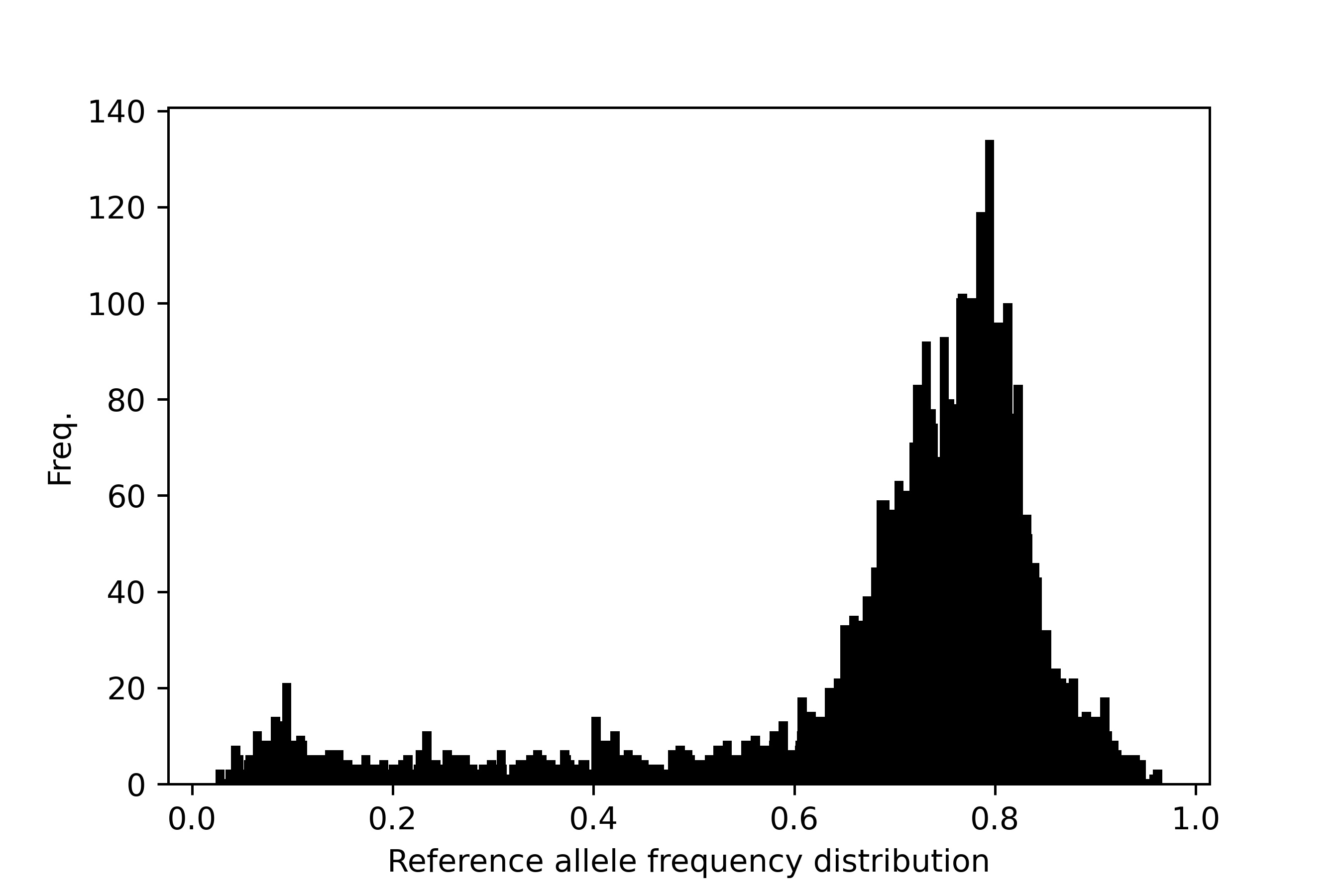
 Figure S6i. Site-frequency spectrum for Chromosome 6.


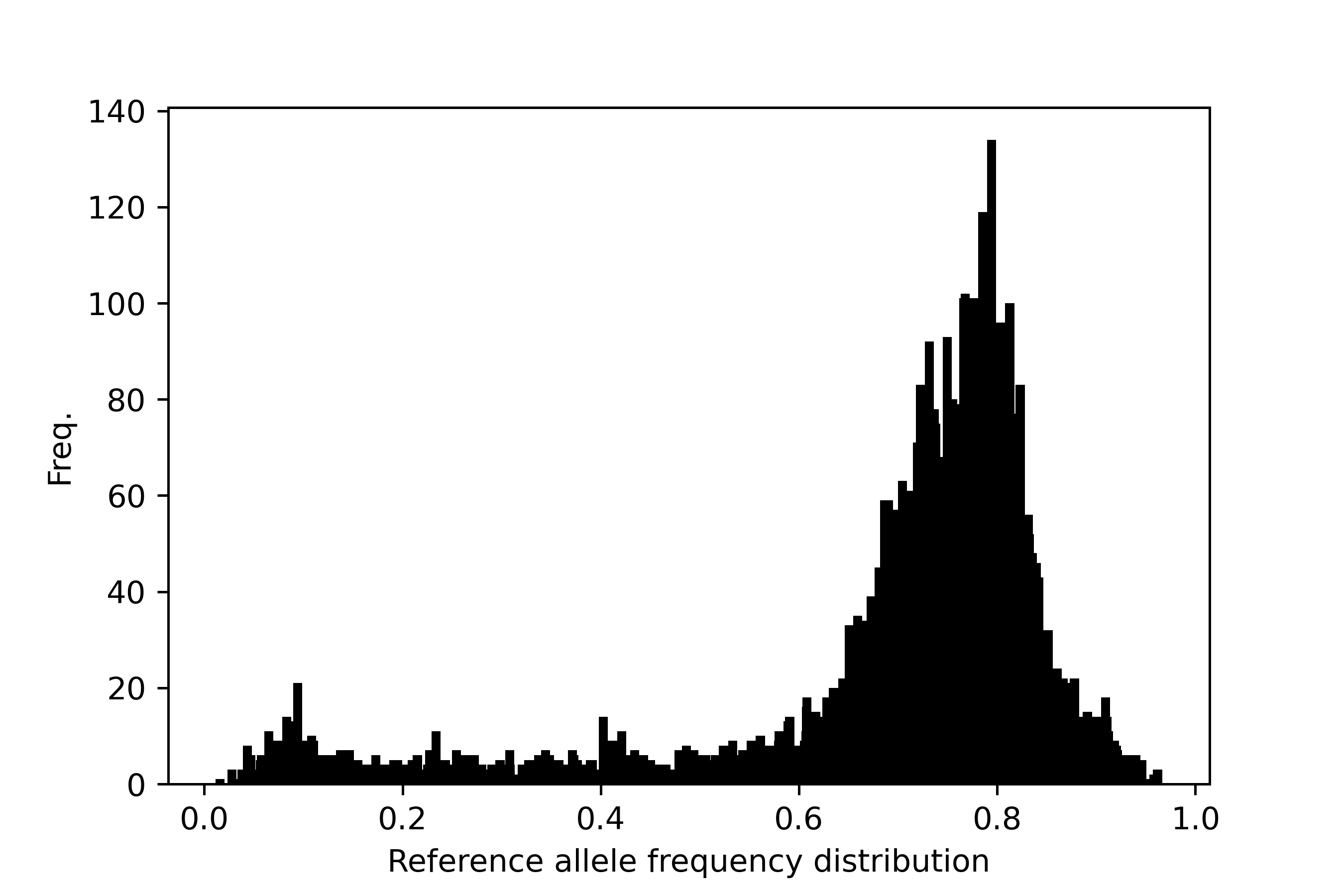
 Figure S6j. Site-frequency spectrum for Chromosome 7.


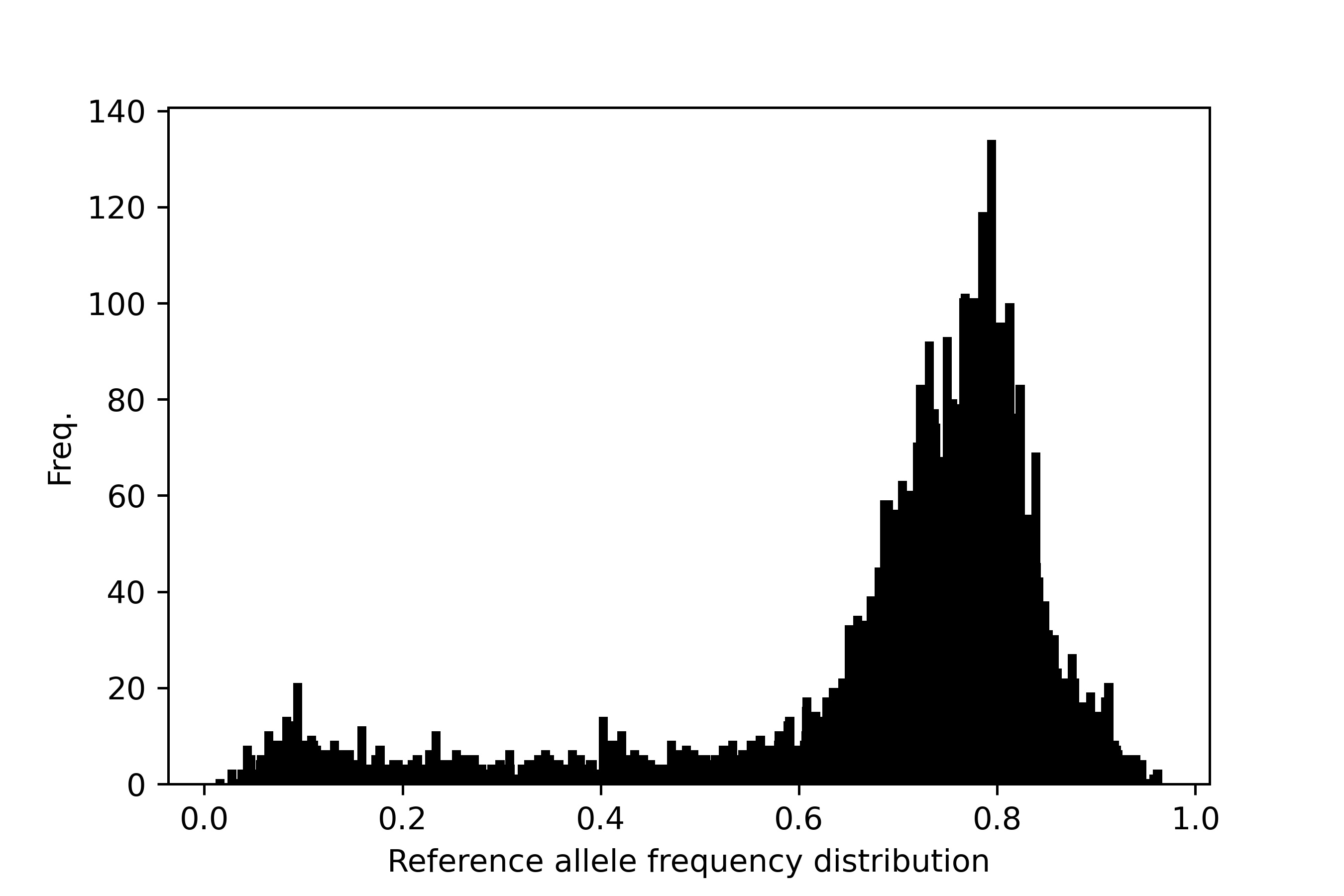
 Figure S6k. Site-frequency spectrum for Chromosome 8.


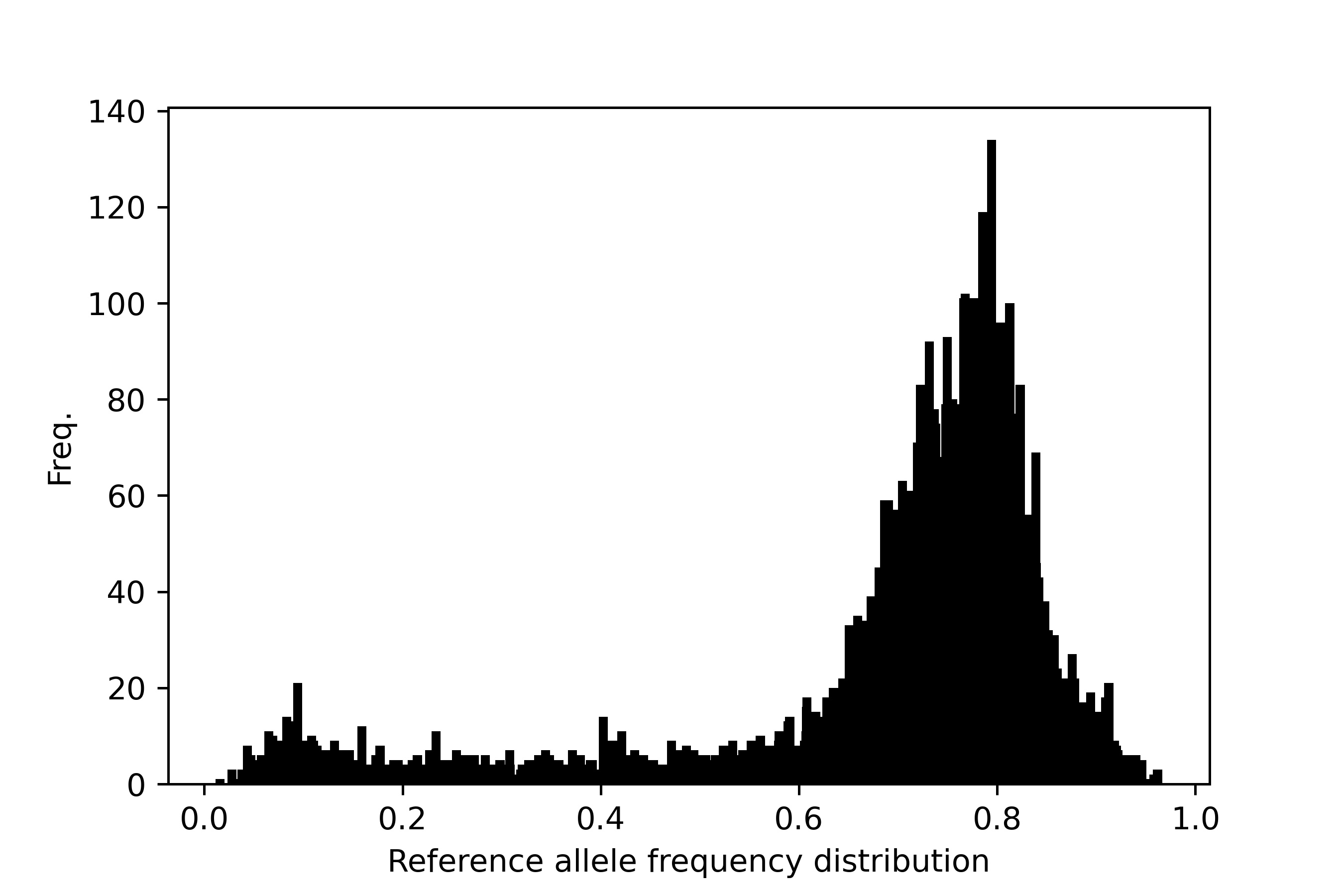
 Figure S6l. Site-frequency spectrum for Chromosome 9.

Table S1. Thai Rice Resource (TRR) whole-genome sequences (n=279) used in this study.

| Accession | Variety | Name | GS No. | Type |
| --- | --- | --- | --- | --- |
| W00120 | Aus | DHARIA | IRRI | Foreign donor |
| W00122 | Aus | FR13A | - | Foreign donor |
| W00154 | Aus | Kalubala Vee | - | Foreign donor |
| W00170 | Aus | Dular (GS.20018) | 20018 | Foreign donor |
| W00177 | Aus | CT9993 | - | Foreign donor |
| W00178 | Aus | DH103 | - | Foreign donor |
| W00180 | Aus | DV85 | - | Foreign donor |
| W00083 | Japonica | Knga Kyein Thee | - | Maekhong variety |
| W00088 | Japonica | Kauk Kyi Taung Pyan | - | Maekhong variety |
| W00132 | Japonica | Jao Khao Chiang Mai | 24601 | RD variety |
| W00133 | Japonica | Niaw Daw Chaw Mai Pai 49 | 24612 | RD variety |
| W00167 | Japonica | Hoi Mueng | 14142 | Landrace |
| W00176 | Japonica | Azucena | - | Foreign donor |
| W00195 | Japonica | Pawsan hmwe | - | Maekhong variety |
| W00221 | Japonica | Gai Noi Leuang | - | Maekhong variety |
| W00223 | Japonica | Ai-Tai | 8100 | Landrace |
| W00237 | Japonica | Khao Niaw Leum Pua | 24613 | Landrace |
| W00252 | Japonica | Dawk Pa-yawm GS.23856 | 23856 | Landrace |
| W00258 | Japonica | Nam Roo | 23725 | Landrace |
| W00269 | Japonica | Payah Luem Gaeng | 24841 | Landrace |
| W00306 | Japonica | Hawm Puang | 8113 | Landrace |
| W00319 | Japonica | Sai Rai | 14331 | Landrace |
| W00025 | Indica | Jao Hawm Nin | - | RGD improved line |
| W00039 | Indica | KD105 UBN | - | RD variety |
| W00040 | Indica | Pokkali | - | Foreign donor |
| W00052 | Indica | Puang Tawng (GS.18442) | 18442 | Landrace |
| W00053 | Indica | PSL85063-9-1-2 | 20663 | Misc |
| W00054 | Indica | IRRIC-BPH-WRI-67-111 | - | Misc |
| W00055 | Indica | Nahng Cha-lawng | 2023 | Landrace |
| W00056 | Indica | RD69 (Thabthim Chumphae) | RD | RD variety |
| W00057 | Indica | Khi Tom Gamnan | 7627 | Landrace |
| W00058 | Indica | T6-4 | - | RGD improved line |
| W00059 | Indica | Khao Nok | 4478 | Landrace |
| W00060 | Indica | Man Ped 215-33-65 | 616 | Landrace |
| W00061 | Indica | BENARA | 18580 | Foreign donor |
| W00062 | Indica | Pinkaset 4 | - | RGD improved line |
| W00063 | Indica | Khao Jao Hawm Phitsanulok 1 | 23409 | RD variety |
| W00065 | Indica | Daw Muey | 13773 | Landrace |
| W00066 | Indica | RGD16054(4)-MAS-228 | - | RGD improved line |
| W00067 | Indica | CHEMPAN | 18145 | Foreign donor |
| W00068 | Indica | Khao Jao Hawm Supanburi | 23062 | RD variety |
| W00069 | Indica | RD51 | - | RD variety |
| W00070 | Indica | RD57 | RD | RD variety |
| W00071 | Indica | T6-6 | - | RGD improved line |
| W00072 | Indica | IR60 | 15963 | IRRI |
| W00073 | Indica | Rice Berry | - | RGD improved line |
| W00074 | Indica | Khao Gaw Diaw 35 | 24607 | RD variety |
| W00075 | Indica | Surin1 | 2705 | RD variety |
| W00076 | Indica | CSSL-Chr1 (11) | - | RGD improved line |
| W00077 | Indica | Chaw Lung 97 | 24610 | RD variety |
| W00078 | Indica | MeeKauk | - | Maekhong variety |
| W00079 | Indica | HomMali841 | - | RGD improved line |
| W00080 | Indica | Khao Jao Hawm Klongluang 1 | 23061 | RD variety |
| W00081 | Indica | RD33 | - | RD variety |
| W00082 | Indica | Kra dook Ngoo | 23179 | Landrace |
| W00084 | Indica | Khao Bahn Pong 132 | 867 | RD variety |
| W00085 | Indica | RD15 | 19326 | RD variety |
| W00086 | Indica | Khem Tawng Phatthalung | 17758 | RD variety |
| W00087 | Indica | Supan Buri 3 | 13747 | RD variety |
| W00089 | Indica | Plai Ngahm Prachinburi | 20846 | RD variety |
| W00090 | Indica | IR58 | 18714 | IRRI |
| W00091 | Indica | CSSL-Chr1 (16) | - | RGD improved line |
| W00092 | Indica | Improved Sinthulette-SalTol | - | Maekhong variety |
| W00093 | Indica | Thunyasirin | - | RGD improved line |
| W00094 | Indica | RD29 | 24534 | RD variety |
| W00095 | Indica | RD45 | 24608 | RD variety |
| W00096 | Indica | Phitsanulok 80 | 24547 | RD variety |
| W00097 | Indica | IR40 | - | IRRI |
| W00098 | Indica | Beu Saw Mi | 23719 | Landrace |
| W00099 | Indica | RD17 | 3999 | RD variety |
| W00101 | Indica | Hawm Cholasit | - | RGD improved line |
| W00102 | Indica | RD-MAEJO2 | RD | RD variety |
| W00103 | Indica | CNTLR82006-KSR-2-7 | 20412 | Misc |
| W00104 | Indica | RD43 | 24606 | RD variety |
| W00105 | Indica | Di ngoo-leum | 19117 | Landrace |
| W00106 | Indica | Mu9962 | - | RGD improved line |
| W00108 | Indica | RD7 | 18432 | RD variety |
| W00109 | Indica | Supan Buri 2 | 20776 | RD variety |
| W00110 | Indica | RD41 | 24605 | RD variety |
| W00112 | Indica | Supan Buri 1 | 13745 | RD variety |
| W00113 | Indica | RD21 | 4791 | RD variety |
| W00114 | Indica | Pathum Thani 1 | 23898 | RD variety |
| W00116 | Indica | 1094-61 | - | Misc |
| W00117 | Indica | BA7 | - | Foreign donor |
| W00119 | Indica | BRJ1-13-B-55 | 3022 | Misc |
| W00121 | Indica | FL496 | - | Foreign donor |
| W00123 | Indica | HomMali803-1 | - | RGD improved line |
| W00124 | Indica | HomMali803-2 | - | RGD improved line |
| W00125 | Indica | HomMali821 | - | RGD improved line |
| W00126 | Indica | IR1188 | - | Foreign donor |
| W00127 | Indica | IR64 | - | IRRI |
| W00128 | Indica | IR72 | - | IRRI |
| W00129 | Indica | Nanobokra | - | Foreign donor |
| W00130 | Indica | No.51-Pijit | - | Misc |
| W00131 | Indica | PK1-PY-BLB-2 | - | RGD improved line |
| W00134 | Indica | Niaw Ubon1 | 23407 | RD variety |
| W00136 | Indica | RD14 | 24604 | RD variety |
| W00137 | Indica | RD23 | 19325 | RD variety |
| W00138 | Indica | Khao Pong Kai | 21857 | Landrace |
| W00139 | Indica | Jampah Sawn | 4925 | Landrace |
| W00140 | Indica | Daw Sahm Deuan | 19609 | Landrace |
| W00141 | Indica | Nahng Khiaw | 9359 | Landrace |
| W00143 | Indica | Phitsanulok 2 | 24591 | RD variety |
| W00144 | Indica | Supan Buri 4 | - | Misc |
| W00145 | Indica | Hawm Poo Khiaw | 24616 | Landrace |
| W00146 | Indica | Hawm Supan | 22048 | Landrace |
| W00147 | Indica | Bio.S-No.12 | - | RGD improved line |
| W00148 | Indica | Bio.S-No.38 | - | RGD improved line |
| W00149 | Indica | PTTH09010-14-1-7-4-1No.11 | - | Misc |
| W00150 | Indica | CSSL chula | - | RGD improved line |
| W00151 | Indica | Mu1145 | - | RGD improved line |
| W00152 | Indica | Mu2168 | - | RGD improved line |
| W00153 | Indica | Niaw Gaen Jan (GS.4229) | - | Landrace |
| W00155 | Indica | Khao Ah-Kaht | 22358 | Landrace |
| W00156 | Indica | Koo Mueang | 21795 | Landrace |
| W00157 | Indica | Chaw Ma-kawk | 2042 | Landrace |
| W00159 | Indica | Ta Pow Lum | - | Landrace |
| W00160 | Indica | Tah-bahn | - | Maekhong variety |
| W00161 | Indica | Tawng Rahk Sai | 10917 | Landrace |
| W00162 | Indica | Pratahn Banbung | 20375 | Landrace |
| W00163 | Indica | Puang Nahk | 21167 | Landrace |
| W00164 | Indica | Meuang Ngah | 22785 | Maekhong variety |
| W00165 | Indica | Ruang Diaw | | Landrace |
| W00166 | Indica | Lao Taeng | 21782 | Landrace |
| W00168 | Indica | Leuang Chum Phae | - | Landrace |
| W00169 | Indica | Leuang Dong | - | Landrace |
| W00171 | Indica | Jao Hawm Nin | - | RGD improved line |
| W00172 | Indica | Hawm Sutabut | - | Landrace |
| W00174 | Indica | 716 54R | - | Misc |
| W00175 | Indica | Abhaya | - | Foreign donor |
| W00179 | Indica | DH212 | - | Foreign donor |
| W00181 | Indica | FL530 | - | Foreign donor |
| W00183 | Indica | HomMali805 | - | RGD improved line |
| W00184 | Indica | IR29 | - | IRRI |
| W00186 | Indica | IR53936 | - | IRRI |
| W00187 | Indica | IR57514 | - | IRRI |
| W00188 | Indica | IR62266 | - | IRRI |
| W00189 | Indica | IWS10 | - | RGD improved line |
| W00190 | Indica | Jasmine IR57514 (BC4F7) | - | RGD improved line |
| W00191 | Indica | KDML105-PlusIII | - | RGD improved line |
| W00192 | Indica | MNTK 75 | - | Maekhong variety |
| W00193 | Indica | Mudgo | 18126 | Foreign donor |
| W00194 | Indica | No.51-PSL | - | Misc |
| W00196 | Indica | PTB33 | - | Foreign donor |
| W00197 | Indica | Rathu heenati | - | Foreign donor |
| W00198 | Indica | TDK1_Sub1 | - | Maekhong variety |
| W00199 | Indica | Gao Rueng 88 | 972 | RD variety |
| W00201 | Indica | Khiaw Leu | 3133 | Landrace |
| W00204 | Indica | Chiang Pattalung | 21964 | RD variety |
| W00205 | Indica | Leb Nok Pattani | 21962 | RD variety |
| W00206 | Indica | Satti | 5678 | Landrace |
| W00207 | Indica | Sian Oon | 12719 | Landrace |
| W00208 | Indica | Niaw Khiaw Ngoo (GS.7545) | 7545 | Landrace |
| W00209 | Indica | Niaw Dam | 18014 | Landrace |
| W00210 | Indica | Niaw San-pah Tawng | 1370 | RD variety |
| W00211 | Indica | Niaw Hawm (GS.4915) | 4915 | Landrace |
| W00212 | Indica | Niaw Ubon2 | 23408 | RD variety |
| W00213 | Indica | Muey Nawng 62 M | 19709 | RD variety |
| W00214 | Indica | Leuang Yai | 15906 | Landrace |
| W00215 | Indica | Leuang Yai 148 | 1705 | RD variety |
| W00216 | Indica | Leuang Kha-min | 1312 | Landrace |
| W00217 | Indica | Luang Pratew 123 | 6754 | RD variety |
| W00218 | Indica | Leuang Plai Lah | 6434 | Landrace |
| W00219 | Indica | Leuang Hawm | 6465 | Landrace |
| W00220 | Indica | Phare | 14003 | Landrace |
| W00222 | Indica | Khai Mod Rin 3 | 24611 | RD variety |
| W00224 | Indica | RD10 | 21643 | RD variety |
| W00225 | Indica | RD11 | 13014 | RD variety |
| W00226 | Indica | RD19 | - | RD variety |
| W00227 | Indica | RD27 | 7125 | RD variety |
| W00228 | Indica | RD31 | 24533 | RD variety |
| W00229 | Indica | RD37 | 24602 | RD variety |
| W00231 | Indica | RD5 | 1650 | RD variety |
| W00232 | Indica | RD6 | 22788 | RD variety |
| W00233 | Indica | RD8 | 22051 | RD variety |
| W00234 | Indica | RD9 | 2601 | RD variety |
| W00235 | Indica | Gon Gaew (GS.1698) | 1698 | Landrace |
| W00236 | Indica | Goo Meuang Luang | 4002 | RD variety |
| W00238 | Indica | Khao Gon Jud | 3226 | Landrace |
| W00239 | Indica | Khao Ngaw Chahng | 7292 | Landrace |
| W00240 | Indica | Khao Dawk Mali | 4914 | Landrace |
| W00241 | Indica | Khao Tah Haeng 17 | 828 | RD variety |
| W00242 | Indica | Khao Niaw Nah 432 | 24614 | RD variety |
| W00243 | Indica | Khao Bun Mah | 17965 | Landrace |
| W00244 | Indica | Khao Pahn Luang | 10847 | Landrace |
| W00245 | Indica | Khi Tom Glahng | 23232 | Landrace |
| W00246 | Indica | Khi Tom Pan | 22783 | Landrace |
| W00247 | Indica | Ngah Chang | 15548 | Landrace |
| W00248 | Indica | Jampah Chin | 2056 | Landrace |
| W00250 | Indica | Chai Nat 2 | 24393 | RD variety |
| W00251 | Indica | Chum Phae 60 | 16235 | RD variety |
| W00253 | Indica | Daw Khao GS.5584 | 5584 | Landrace |
| W00254 | Indica | Di Si | 15982 | Landrace |
| W00255 | Indica | Ta Pow Gaew 161 | 16580 | RD variety |
| W00256 | Indica | Tab Mei Dam | 3238 | Landrace |
| W00257 | Indica | Nahng Mon S-4 | 1289 | RD variety |
| W00259 | Indica | Nam Sagui 19 | 15833 | RD variety |
| W00260 | Indica | Bak Muay | 3257 | Landrace |
| W00263 | Indica | Pathum Thani 60 | 17770 | RD variety |
| W00264 | Indica | Prachin Buri1 | 23406 | RD variety |
| W00265 | Indica | Prachin Buri2 | 24592 | RD variety |
| W00266 | Indica | Plawng Aew (GS.17711) | 17711 | Landrace |
| W00267 | Indica | Pinkaset 3 | - | RGD improved line |
| W00268 | Indica | Pin Gaew 56 | 1284 | RD variety |
| W00270 | Indica | Pamah Haek Kuk (GS.19319) | 19319 | Landrace |
| W00271 | Indica | Pamah Dam | 12519 | Landrace |
| W00273 | Indica | Pahn Tawng | 7560 | Landrace |
| W00274 | Indica | Phitsanulok 3 | 24546 | RD variety |
| W00275 | Indica | Phitsanulok 60-1 | 16233 | RD variety |
| W00277 | Indica | Mali Daeng | 10292 | Landrace |
| W00278 | Indica | Mali Gomen | 24617 | RD variety |
| W00279 | Indica | Mah Bang | 5801 | Landrace |
| W00280 | Indica | Yi Tae Sah Ming | 15789 | Landrace |
| W00281 | Indica | Look Leuang | 15657 | Landrace |
| W00282 | Indica | Look Daeng Pattani | 21963 | RD variety |
| W00283 | Indica | Sangyod | 15101 | Landrace |
| W00284 | Indica | Sangyod Phatthalung | 24625 | RD variety |
| W00285 | Indica | San Pah-tawng 1 | 24590 | RD variety |
| W00286 | Indica | Sin Lek | - | RGD improved line |
| W00287 | Indica | Supanburi 60 | 16240 | RD variety |
| W00288 | Indica | Supanburi 90 | 19869 | RD variety |
| W00289 | Indica | Mahk Nam | 22835 | Landrace |
| W00290 | Indica | Luang Pratan (GS.6440) | 6440 | Landrace |
| W00292 | Indica | Hawm Jan(GS.3008) | 3008 | Landrace |
| W00293 | Indica | Hawm Dong | 5787 | Landrace |
| W00294 | Indica | Hawm Nai Pon | 6836 | Landrace |
| W00295 | Indica | Hawm Pamah (GS.5333) | 5333 | Landrace |
| W00296 | Indica | Hawm Lao | 18992 | Landrace |
| W00297 | Indica | Han Trah 60 | 16579 | RD variety |
| W00298 | Indica | Hahng Yi 71 | 7613 | RD variety |
| W00299 | Indica | Hah Ruang Bow | 15974 | Landrace |
| W00300 | Indica | Ayutthaya 1 | 24595 | RD variety |
| W00301 | Indica | Niaw Hawm (GS.9194) | 9194 | Landrace |
| W00302 | Indica | Khao Dawk Mali 105 | 13743 | Landrace |
| W00303 | Indica | Khao Tah Haeng | 12275 | Landrace |
| W00304 | Indica | Pin Gaew | 14148 | Landrace |
| W00305 | Indica | Pueng Tawng (GS.574) | 574 | Landrace |
| W00307 | Indica | Hahng Nahk | 5583 | Landrace |
| W00309 | Indica | Kon Jud | 21389 | Landrace |
| W00310 | Indica | Kao Gam | 15722 | Landrace |
| W00311 | Indica | Jod Mawn | 1693 | Landrace |
| W00312 | Indica | Jao Khao | 3330 | Landrace |
| W00314 | Indica | Dawk Khah | 12160 | Landrace |
| W00315 | Indica | Daw Dawk Mai | 22817 | Landrace |
| W00316 | Indica | Daeng Nah | - | Landrace |
| W00317 | Indica | Tom Meuang Luang | - | Landrace |
| W00318 | Indica | Tah-khiad | - | Maekhong variety |
| W00321 | Indica | Puang Sung | - | Landrace |
| W00322 | Indica | Puang Hahng Nahk | - | Landrace |
| W00323 | Indica | Rahk Haeng | 12616 | Landrace |
| W00324 | Indica | Hawm Tawng | 20575 | Landrace |
| W00325 | Indica | Hawm Nahng Naun | - | Landrace |
| W00326 | Indica | Niaw Mali | - | Landrace |
| W00327 | Indica | Leuang Kai Lah (GS.5555) | 5555 | Landrace |
| W00328 | Indica | Leuang Ngahm | - | Landrace |
| W00329 | Indica | Leuang Tawng (GS.5556) | 5556 | Landrace |
| W00331 | Indica | CNT1-Qbph6-12(fromAB) | - | RGD improved line |
| W00332 | Indica | CNTBR82040-259-1-1-1 | 20472 | Misc |
| W00333 | Indica | CSSL-Chr8 (106) | - | RGD improved line |
| W00334 | Indica | HomMali802 | - | RGD improved line |
| W00335 | Indica | HomMali823 | - | RGD improved line |
| W00336 | Indica | IR4563-52-1-3-6 | 7793 | IRRI |
| W00337 | Indica | KD20 | - | RGD improved line |
| W00338 | Indica | KDML105-Bph3-1 | - | RGD improved line |
| W00339 | Indica | TDK1_Aroma | - | Maekhong variety |
| W00340 | Indica | Chiang Dao | 19103 | Landrace |
| W00341 | Indica | Gaen Jan | 3247 | Landrace |
| W00342 | Indica | Mae Lahd | 15968 | Landrace |
| W00343 | Indica | RD13 | 16410 | RD variety |
| W00344 | Indica | RD35 | 24600 | RD variety |
| W00346 | Indica | Gon Gaew (GS.6158) | 6158 | Landrace |
| W00347 | Indica | Khao Glahng | 3639 | Landrace |
| W00348 | Indica | Pinkaset 1 | - | RGD improved line |
| W00349 | Indica | Pueng Nak | 1691 | Landrace |
| W00350 | Indica | Sinuan | 19838 | Landrace |
| W00351 | Indica | Hawm Daeng Noi | 6728 | Landrace |
| W00352 | Indica | E-pid | 12484 | Landrace |
| W00354 | Indica | Daw Khao GS.12155 | 12155 | Landrace |
| W00356 | Indica | Hawm Pamah (GS.19843) | 19843 | Landrace |

Table S3. Rice accession whole-genome sequences (n=200) downloaded from IRRI.

| Accession no. | Sub-population | Variety | Country | Region | Grain length (mm) |
| --- | --- | --- | --- | --- | --- |
| IRIS_313-11258 | aro | aro | India | subcont | 5.5 |
| IRIS_313-8326 | aro | aro | India | subcont | 6.2 |
| IRIS_313-11270 | aro | aro | India | subcont | 8 |
| IRIS_313-11289 | aro | aro | India | subcont | 8.6 |
| IRIS_313-11052 | aus | aus | Bangladesh | subcont | 7.5 |
| IRIS_313-11058 | aus | aus | Bangladesh | subcont | 8.6 |
| IRIS_313-11737 | aus | aus | India | subcont | 6.7 |
| IRIS_313-11298 | aus | aus | India | subcont | 7.1 |
| IRIS_313-10852 | aus | aus | India | subcont | 7.2 |
| IRIS_313-11809 | aus | aus | Kenya | africa | 10.5 |
| IRIS_313-11796 | ind1A | indica | China | china | 8.3 |
| IRIS_313-11668 | ind1A | indica | China | china | 8.5 |
| IRIS_313-11802 | ind1A | indica | China | china | 8.6 |
| IRIS_313-11805 | ind1A | indica | China | china | 8.8 |
| IRIS_313-11665 | ind1A | indica | China | china | 9 |
| IRIS_313-11798 | ind1B | indica | China | china | 9.1 |
| IRIS_313-11643 | ind1B | indica | India | subcont | 9.7 |
| IRIS_313-11251 | ind1B | indica | Philippines | indo | 8.4 |
| IRIS_313-11723 | ind2 | indica | Guinea | africa | 10.8 |
| IRIS_313-11645 | ind2 | indica | India | subcont | 8.8 |
| IRIS_313-11646 | ind2 | indica | India | subcont | 9.7 |
| IRIS_313-11794 | ind2 | indica | Madagascar | africa | 10.1 |
| IRIS_313-11728 | ind3 | indica | China | china | 10.1 |
| IRIS_313-8493 | ind3 | indica | Indonesia | indo | 7.5 |
| IRIS_313-11812 | ind3 | indica | Kenya | africa | 10.2 |
| IRIS_313-8312 | ind3 | indica | Malaysia | indo | 8 |
| IRIS_313-9409 | ind3 | indica | Malaysia | indo | 9.6 |
| IRIS_313-11819 | ind3 | indica | Myanmar | se.asia | 9.5 |
| IRIS_313-10177 | indx | indica | China | china | 7.6 |
| IRIS_313-11748 | indx | indica | China | china | 8.7 |
| IRIS_313-11744 | indx | indica | China | china | 8.9 |
| IRIS_313-11807 | indx | indica | Colombia | lat.am | 9.6 |
| IRIS_313-11740 | indx | indica | Ghana | africa | 8 |
| IRIS_313-10337 | indx | indica | Indonesia | indo | 8.4 |
| IRIS_313-11717 | indx | indica | Indonesia | indo | 8.8 |
| IRIS_313-11817 | indx | indica | Myanmar | se.asia | 9.7 |
| IRIS_313-10870 | subtrop | japonica | India | subcont | 5.7 |
| IRIS_313-12349 | subtrop | japonica | Lao People's Democratic Republic | se.asia | 8.8 |
| IRIS_313-11094 | subtrop | japonica | Lao People's Democratic Republic | se.asia | 11.1 |
| IRIS_313-11661 | temp | japonica | Bhutan | subcont | 8.9 |
| IRIS_313-11651 | temp | japonica | China | china | 7.5 |
| IRIS_313-11652 | temp | japonica | China | china | 7.6 |
| IRIS_313-11725 | temp | japonica | Japan | china | 7.7 |
| IRIS_313-11689 | temp | japonica | Korea, Republic of | china | 7.1 |
| IRIS_313-11759 | trop | japonica | Cote d'Ivoire | africa | 8.8 |
| IRIS_313-11755 | trop | japonica | Liberia | africa | 9.4 |
| IRIS_313-11790 | trop | japonica | Madagascar | africa | 8.2 |
| IRIS_313-11673 | trop | japonica | Philippines | indo | 7.4 |
| IRIS_313-11736 | trop | japonica | Philippines | indo | 7.7 |
| IRIS_313-11297 | admix | admix | India | subcont | 7 |
| IRIS_313-10883 | admix | admix | India | subcont | 10.8 |
| IRIS_313-12094 | aro | aro | Bangladesh | subcont | 4.7 |
| IRIS_313-11026 | aro | aro | Pakistan | subcont | 10.2 |
| IRIS_313-12141 | aus | aus | Bangladesh | subcont | 6 |
| IRIS_313-8390 | aus | aus | Pakistan | subcont | 10.9 |
| IRIS_313-9039 | ind2 | indica | Sri Lanka | subcont | 5.7 |
| IRIS_313-11280 | indx | indica | India | subcont | 11 |
| IRIS_313-10888 | subtrop | japonica | India | subcont | 10.1 |
| IRIS_313-9778 | admix | admix | Argentina | lat.am | 8 |
| IRIS_313-9464 | admix | admix | Surinam | lat.am | 11.8 |
| IRIS_313-10969 | aus | aus | Brazil | lat.am | 8.5 |
| IRIS_313-11411 | ind2 | indica | Brazil | lat.am | 7.6 |
| IRIS_313-9841 | indx | indica | Surinam | lat.am | 11.4 |
| IRIS_313-9771 | temp | japonica | Austria | lat.am | 7.1 |
| IRIS_313-11428 | trop | japonica | Brazil | lat.am | 10.6 |
| IRIS_313-10025 | admix | admix | Madagascar | africa | 7.7 |
| IRIS_313-11767 | admix | admix | Madagascar | africa | 11.3 |
| IRIS_313-11765 | aro | aro | Liberia | africa | 6.5 |
| IRIS_313-10032 | aro | aro | Madagascar | africa | 8.7 |
| IRIS_313-12118 | ind2 | indica | Madagascar | africa | 6 |
| IRIS_313-11814 | ind3 | indica | Kenya | africa | 11.2 |
| IRIS_313-11121 | trop | japonica | Liberia | africa | 7.9 |
| IRIS_313-9742 | trop | japonica | Madagascar | africa | 10.7 |
| IRIS_313-10738 | admix | admix | Indonesia | indo | 7.4 |
| IRIS_313-9101 | admix | admix | Malaysia | indo | 9.9 |
| IRIS_313-10380 | aus | aus | Philippines | indo | 7 |
| IRIS_313-10955 | ind3 | indica | Indonesia | indo | 5.9 |
| IRIS_313-11235 | indx | indica | Philippines | indo | 11.4 |
| IRIS_313-9616 | trop | japonica | Philippines | indo | 5.7 |
| IRIS_313-11971 | trop | japonica | Indonesia | indo | 10.9 |
| IRIS_313-8873 | admix | admix | Taiwan | china | 7.3 |
| IRIS_313-10585 | admix | admix | Japan | china | 7.5 |
| IRIS_313-11698 | aro | aro | Taiwan | china | 7.1 |
| IRIS_313-11157 | ind1A | indica | Taiwan | china | 6.1 |
| IRIS_313-12061 | temp | japonica | China | china | 6.2 |
| IRIS_313-10079 | trop | japonica | Japan | china | 9.4 |
| IRIS_313-11136 | admix | admix | Myanmar | se.asia | 7.6 |
| IRIS_313-11092 | admix | admix | Lao People's Democratic Republic | se.asia | 9.9 |
| IRIS_313-12074 | aro | aro | Myanmar | se.asia | 6.9 |
| IRIS_313-12148 | ind3 | indica | Cambodia | se.asia | 5.8 |
| IRIS_313-10654 | ind3 | indica | Lao People's Democratic Republic | se.asia | 10.8 |
| IRIS_313-12164 | japx | japonica | Cambodia | se.asia | 6.2 |
| IRIS_313-12341 | subtrop | japonica | Lao People's Democratic Republic | se.asia | 12.4 |
| IRIS_313-11189 | admix | admix | Union of Soviet Socialist Republics (Former) | europe | 9.8 |
| IRIS_313-8747 | aro | aro | Iran | europe | 10 |
| IRIS_313-12016 | indx | indica | Egypt | europe | 9 |
| IRIS_313-9817 | ind2 | indica | Netherlands | europe | 12 |
| IRIS_313-9813 | temp | japonica | Hungary | europe | 7.2 |
| IRIS_313-10061 | japx | japonica | Portugal | europe | 12.7 |
| IRIS_313-9795 | ind2 | indica | Australia | australia | 7.8 |
| IRIS_313-10010 | ind2 | indica | Fiji | indo | 8 |
| IRIS_313-10014 | temp | japonica | Italy | europe | 9.1 |
| IRIS_313-10020 | aus | aus | Sri Lanka | subcont | 7.9 |
| IRIS_313-10034 | indx | indica | Niger | africa | 10.7 |
| IRIS_313-10046 | ind1B | indica | Zambia | africa | 8.5 |
| IRIS_313-10047 | ind1A | indica | Nigeria | africa | 8.1 |
| IRIS_313-10054 | indx | indica | Panama | lat.am | 10.4 |
| IRIS_313-10113 | indx | indica | Mozambique | africa | 8.9 |
| IRIS_313-10289 | ind1B | indica | Venezuela | lat.am | 9.4 |
| IRIS_313-10301 | indx | indica | Brazil | lat.am | 10 |
| IRIS_313-10397 | ind1B | indica | Colombia | lat.am | 10 |
| IRIS_313-10484 | ind1A | indica | Philippines | indo | 8.2 |
| IRIS_313-10516 | indx | indica | United States of America | lat.am | 8.6 |
| IRIS_313-10572 | indx | indica | Nigeria | africa | 7.8 |
| IRIS_313-10573 | ind2 | indica | Mali | africa | 6.3 |
| IRIS_313-10614 | indx | indica | Hong Kong | china | 8.5 |
| IRIS_313-10640 | ind2 | indica | India | subcont | 11 |
| IRIS_313-10705 | indx | indica | Malaysia | indo | 10.1 |
| IRIS_313-10712 | trop | japonica | Cote d'Ivoire | africa | 9.6 |
| IRIS_313-10722 | trop | japonica | Sri Lanka | subcont | 6.8 |
| IRIS_313-10723 | ind2 | indica | Senegal | africa | 8.1 |
| IRIS_313-10725 | indx | indica | Senegal | africa | 8.7 |
| IRIS_313-10726 | ind3 | indica | Senegal | africa | 9.2 |
| IRIS_313-10733 | indx | indica | Nepal | subcont | 9.6 |
| IRIS_313-11056 | aus | aus | Bangladesh | subcont | 9.6 |
| IRIS_313-11075 | japx | japonica | Lao People's Democratic Republic | se.asia | 8.8 |
| IRIS_313-11120 | ind2 | indica | Bhutan | subcont | 6.5 |
| IRIS_313-11141 | indx | indica | Myanmar | se.asia | 7.7 |
| IRIS_313-11149 | ind3 | indica | Myanmar | se.asia | 10.3 |
| IRIS_313-11153 | temp | japonica | India | subcont | 7.1 |
| IRIS_313-11162 | ind1B | indica | Indonesia | indo | 10.6 |
| IRIS_313-11335 | ind2 | indica | Philippines | indo | 8 |
| IRIS_313-11336 | temp | japonica | Philippines | indo | 8 |
| IRIS_313-11404 | ind2 | indica | Bangladesh | subcont | 6.1 |
| IRIS_313-11425 | admix | admix | Brazil | lat.am | 9.7 |
| IRIS_313-11426 | trop | japonica | Brazil | lat.am | 7.7 |
| IRIS_313-11437 | ind1B | indica | Mexico | lat.am | 10.1 |
| IRIS_313-11438 | indx | indica | Mexico | lat.am | 9.9 |
| IRIS_313-11439 | trop | japonica | Cuba | lat.am | 8.9 |
| IRIS_313-11510 | indx | indica | Sri Lanka | subcont | 10 |
| IRIS_313-11565 | ind2 | indica | Nepal | subcont | 8.4 |
| IRIS_313-11574 | temp | japonica | China | china | 8.6 |
| IRIS_313-11591 | admix | admix | Malaysia | indo | 8.6 |
| IRIS_313-11623 | subtrop | japonica | China | china | 8.7 |
| IRIS_313-11716 | ind3 | indica | Guinea | africa | 9.5 |
| IRIS_313-11758 | ind2 | indica | Cote d'Ivoire | africa | 8 |
| IRIS_313-11779 | ind3 | indica | Tanzania | africa | 9.2 |
| IRIS_313-11783 | indx | indica | Gambia | africa | 9.2 |
| IRIS_313-11787 | ind2 | indica | Gambia | africa | 8.9 |
| IRIS_313-11797 | ind1A | indica | China | china | 10 |
| IRIS_313-11808 | indx | indica | Colombia | lat.am | 10.2 |
| IRIS_313-11830 | ind3 | indica | Gambia | africa | 7.7 |
| IRIS_313-11932 | ind2 | indica | Kenya | africa | 9.9 |
| IRIS_313-11938 | indx | indica | Burkina Faso | africa | 7.9 |
| IRIS_313-11940 | indx | indica | Burkina Faso | africa | 8.5 |
| IRIS_313-11956 | admix | admix | Nepal | subcont | 7.8 |
| IRIS_313-11976 | ind3 | indica | Sierra Leone | africa | 7.6 |
| IRIS_313-11977 | indx | indica | Madagascar | africa | 7.6 |
| IRIS_313-11988 | indx | indica | Sierra Leone | africa | 7.5 |
| IRIS_313-11989 | ind3 | indica | Brunei Darussalam | indo | 7.4 |
| IRIS_313-12015 | ind2 | indica | Nigeria | africa | 9.3 |
| IRIS_313-12017 | indx | indica | Ghana | africa | 7.9 |
| IRIS_313-12018 | trop | japonica | Sierra Leone | africa | 9.2 |
| IRIS_313-12060 | japx | japonica | China | china | 7 |
| IRIS_313-12130 | ind3 | indica | Lao People's Democratic Republic | se.asia | 6.5 |
| IRIS_313-12138 | indx | indica | Bhutan | subcont | 6.5 |
| IRIS_313-12220 | indx | indica | Lao People's Democratic Republic | se.asia | 7.8 |
| IRIS_313-12289 | subtrop | japonica | Myanmar | se.asia | 9.8 |
| IRIS_313-12325 | ind2 | indica | Cameroon | africa | 7.4 |
| IRIS_313-8324 | admix | admix | Indonesia | indo | 8.9 |
| IRIS_313-8342 | aus | aus | Sri Lanka | subcont | 6.1 |
| IRIS_313-8399 | temp | japonica | France | europe | 8 |
| IRIS_313-8571 | ind3 | indica | Tanzania | africa | 10.8 |
| IRIS_313-8669 | japx | japonica | United States of America | lat.am | 8.4 |
| IRIS_313-8690 | japx | japonica | Viet Nam | se.asia | 7.9 |
| IRIS_313-8745 | trop | japonica | Haiti | lat.am | 9.4 |
| IRIS_313-8765 | aro | aro | Bhutan | subcont | 6.8 |
| IRIS_313-8890 | temp | japonica | Belgium | europe | 8.1 |
| IRIS_313-8927 | subtrop | japonica | Taiwan | china | 8.6 |
| IRIS_313-8930 | ind1B | indica | Bangladesh | subcont | 7.9 |
| IRIS_313-8996 | ind3 | indica | Viet Nam | se.asia | 10.1 |
| IRIS_313-9176 | japx | japonica | India | subcont | 7.3 |
| IRIS_313-9193 | japx | japonica | Brazil | lat.am | 9.1 |
| IRIS_313-9404 | admix | admix | Bhutan | subcont | 8.4 |
| IRIS_313-9519 | trop | japonica | Surinam | lat.am | 9.1 |
| IRIS_313-9523 | japx | japonica | Japan | china | 7.6 |
| IRIS_313-9557 | ind2 | indica | India | subcont | 5.9 |
| IRIS_313-9648 | admix | admix | United States of America | lat.am | 9.6 |
| IRIS_313-9702 | temp | japonica | Taiwan | china | 8.1 |
| IRIS_313-9774 | japx | japonica | Turkey | europe | 7.5 |
| IRIS_313-9782 | temp | japonica | Peru | lat.am | 7.3 |
| IRIS_313-9783 | admix | admix | Afghanistan | subcont | 9.1 |
| IRIS_313-9898 | ind3 | indica | Portugal | europe | 10.2 |
| IRIS_313-9924 | indx | indica | Korea Republic of | china | 8.5 |
| IRIS_313-9935 | ind2 | indica | Guyana | lat.am | 8 |
| IRIS_313-9939 | admix | admix | Surinam | lat.am | 9.1 |
| IRIS_313-9940 | ind1B | indica | Guatemala | lat.am | 10.2 |
| IRIS_313-9949 | trop | japonica | Sri Lanka | subcont | 9 |
| IRIS_313-9961 | temp | japonica | Norway | europe | 8.9 |
| IRIS_313-9989 | ind3 | indica | Cote d'Ivoire | africa | 9.3 |
